# Drp1 activates ROS/HIF-1α/EZH2 and triggers mitochondrial fragmentation to deteriorate hypercalcemia-associated neuronal injury in mouse model of chronic kidney disease

**DOI:** 10.1186/s12974-022-02542-7

**Published:** 2022-09-01

**Authors:** Hongming Sun, Xitong Li, Xin Chen, Yingquan Xiong, Yaochen Cao, Ziqiang Wang

**Affiliations:** 1 The First Affiliated Hospital of Hainan Medical University, No. 31 Longhua Road, Haikou, 570102 Hannan China; 2 Department of Neurology and Neuroscience, Okayama University School of Medicine, Okayama, 700-8558 Japan; 3 Department of Nephrology, Charité-Universitätsmedizin Berlin, Campus Mitte, 10117 Berlin, Germany

**Keywords:** Drp1, Chronic kidney disease, Hypercalcemia, Neuronal damage, Mitochondrial fragmentation, HIF-1α, EZH2, ROS production

## Abstract

**Background:**

Chronic kidney disease (CKD), characterized as renal dysfunction, is regarded as a major public health problem which carries a high risk of cardiovascular diseases. The purpose of this study is to evaluate the functional significance of Drp1 in hypercalcemia-associated neuronal damage following CKD and the associated mechanism.

**Methods:**

Initially, the CKD mouse models were established. Next, RT-qPCR and Western blot analysis were performed to measure expression of Fis1 and Drp1 in CKD. Chromatin immunoprecipitation (ChIP) assay and dual-luciferase reporter gene assay were utilized to explore the relationship among Drp1, HIF-1α, EZH2, and ROS with primary cortical neurons isolated from neonatal mice. Next, CKD mice were subjected to calcitonin treatment or manipulation with adenovirus expressing sh-Drp1, so as to explore the effects of Drp1 on hypercalcemia-induced neuronal injury in CKD. TUNEL assay and immunofluorescence staining were performed to detect apoptosis and NeuN-positive cells (neurons) in prefrontal cortical tissues of CKD mice.

**Results:**

It was found that hypercalcemia could induce neuronal injury in CKD mice. An increase of Fis1 and Drp1 expression in cerebral cortex of CKD mice correlated with mitochondrial fragmentation. Calcitonin suppressed Drp1/Fis1-mediated mitochondrial fragmentation to attenuate hypercalcemia-induced neuronal injury after CKD. Additionally, Drp1 could increase EZH2 expression through the binding of HIF-1α to EZH2 promoter via elevating ROS generation. Furthermore, Drp1 knockdown inhibited hypercalcemia-induced neuronal injury in CKD while overexpression of EZH2 could reverse this effect in vivo.

**Conclusion:**

Taken together, the key findings of the current study demonstrate the promotive role of Drp1 in mitochondrial fragmentation which contributes to hypercalcemia-induced neuronal injury in CKD.

**Supplementary Information:**

The online version contains supplementary material available at 10.1186/s12974-022-02542-7.

## Background

Chronic kidney disease (CKD) is considered as a persistent injury of renal parenchyma, resulting in chronic worsening of renal function and gradually developing into end-stage renal disease (ESRD) [1, 2]. CKD affects about 10–15% of the adult population and accounts for around 1.5% of mortality around the world [3, 4]. CKD is reported to be associated with a risk of incident total hypercalcemia in cat [5]. Phosphate-restricted clinical renal diet which is an effective management strategy for CKD may lead to the onset of hypercalcemia [6]. Interestingly, a prior study has demonstrated that low serum calcium correlates with rapid renal function progression in CKD patients [7]. Calcium (Ca^2+^) is a divalent cation which exerts essential role in many metabolic processes including skeletal mineralization, nerve conduction, and blood clotting [8]. Moreover, calcium overload has been reported to drive neuronal cell death during ischemia [9], yet the mechanism remains largely undefined. A better understanding for the mechanism related to hypercalcemia-associated neuronal injury following CKD is potentially conducive to developing novel therapeutics for improving the outcomes of patients with CKD.

Mitochondria are regarded as a highly complex interconnected network of organelles that meet the renal metabolic needs and respond to oxidative stress and inflammation following renal injury [10]. Mitochondrial impairment is regarded a driver of CKD progression [11]. Mitochondrial fragmentation is an early key process leading to mitochondrial membrane leakage and cell death [12] Dynamin-related protein 1 (Drp1) is a key protein recruited by Fis1, Mff, MiD49, and MiD51 for the promotion of mitochondrial fission [13, 14]. It is interesting to note that Drp1-induced mitochondrial fission promotes mitochondrial fragmentation, leading to mitochondrial impairment and neuronal injury in the neurodegenerative diseases [15]. However, whether Drp1-mediated mitochondrial fragmentation affects hypercalcemia-associated neuronal injury following CKD remains unknown. In this study, we developed a mouse model of CKD induced by adenine-containing diet and performed a series of experiments to explore the association of mitochondrial dynamics mediated by Drp1 with neuronal functions after induction of CKD. Further, molecular mechanisms underlying this effect of Drp1 were analyzed to provide a theoretical basis for developing therapeutic neuroprotective targets.

## Methods

### Establishment of CKD mouse models

C57BL/6J male mice (aged 8 weeks; weighing 25 g) were individually caged in a laboratory with controlled temperature (22–25 ℃), humidity (45–50%), and light (12-h light/12-h dark). All mice were given a normal diet 1 week prior to CKD induction with free access to food and water. CKD was induced by feeding the mice an adenine-containing diet, while a normal diet was given for the mice used as control.

Before adenine-containing or normal diet, a small amount of blood was separated from the cheek by microtubule puncture, which were centrifuged at 1500*g* at 4 °C for 10 min to collect the serum. Mice weighing 25*g* were then given a diet containing 0.2% w/w adenine (A2786, Sigma-Aldrich, St. Louis, MO, USA) or normal diet. After 6 weeks, animals were anaesthetized with isoflurane and then euthanized by a cardiac puncture to collect blood samples, which were centrifuged to obtain serum. The contents of blood urea nitrogen (BUN), creatinine, calcium, and phosphorus in serum samples were measured before and after adenine-containing diet. The kidneys were isolated and stained to determine the success of CKD model construction. In addition, the cerebral cortex was isolated and sequenced.

Adeno-associated virus (AAV2, 1.5 × 10^9^/mL) was purchased from Genechem (Shanghai, China). The sequence was designed according to the mouse gene and completed by Genechem. The experimental steps were carried out according to the instructions. In brief, AAV2 (2 nL/s, 2 μL) was stereotactically injected into the cortex of mice (+ 1.000 ML; + 1.800 AP; -1.250 DV). The injection needle remained for 5 min after injection to ensure uniform virus distribution [16].

CKD model mice were injected with normal saline (Vehicle) + Vector, Calcitonin (0.2 μg/g BW) [17] + Vector, Calcitonin (0.2 μg/g BW) + AAV2 expressing Drp1, AAV2 expressing sh-NC + Vector, AAV2 expressing sh-Drp1 + Vector, or AAV2 expressing sh-Drp1 + AAV2 expressing EZH2.

### RNA-sequencing (RNA-Seq)

The cerebral cortex tissues were obtained from 3 CKD-induced mice and 3 normal mice. HiSeqLibrary of 950 ng mRNA was generated using Illumina truseq Stranded Total RNA with Ribo-Zero protocol (Illumina, San Diego, CA). RNA-Seq (Illumina HiSeq) was performed at center for genomics of UW cystic fibrosis, and then image analysis and base calling were conducted using Illumina’s RTA (v1.18) software. Multiplex decomposition and file generation and alignment in FASTQ format were performed using HiSeq analysis software (TopHat v2.1.1) and UCSC mouse genome version mm10. RNA-SeQC v1.1.8 was then utilized for quality control and HTSeq-counts v0.7.2 for generation of counts. Significantly upregulated genes in CKD mice versus normal mice were screened using the limma package in R language (https://bioconductor.org/packages/release/bioc/html/limma.html) with log_2_FC > 0.4 and *p*.value < 0.05 as cut-off values.

### Bioinformatics analysis

Renal failure-related genes were obtained through GeneCards and DisGeNET databases. The Protein by name of online tool STRING was employed to predict the genes interacted with FIS1 and determined the gene with the highest interaction score. The interaction diagram was plotted using Cytoscape.

### Morris water maze test

A circular pool (150 cm diameter, walls 60 cm depth) was used for Morris water maze (escape latency), with water temperature maintained at 20 ~ 25 °C. The pool was divided into four quadrants (Lower right, upper right, lower left, and upper left). The platform was placed in the middle of the lower right quadrant.

After 7 days of training, the mice were put into the water, and the time of looking for the platform (escape latency) within 2 min was recorded. If the mice found the platform within 2 min, the actual escape latency within 2 min was recorded. If the platform was not found within 2 min, the experimenter would lead mice to the platform and stay for 10 s. The escape latency was recorded as 2 min. On the 5th day, the underwater platform was withdrawn to conduct spatial probe test. A water entry point was fixed, and the period for the mouse to stay in the original platform quadrant (the destination quadrant) within 60 s and the times to swim through the original platform position (the times of looping) were recorded.

### Histopathological staining

After 6 weeks, animals were anaesthetized with isoflurane and euthanized by cardiac puncture. Mouse kidney was isolated after perfusion with cold salt solution at a pressure of 100 mmHg. The kidney tissues were fixed in the 10% formaldehyde, dehydrated, immersed in wax, embedded and sliced into sections (2–4 µm).

Histopathological staining of kidney sections was performed with hematoxylin–eosin (H&E) kit (C0105, Beyotime Institute of Biotechnology, Shanghai, China), Masson’s trichrome stain kit (1004850001, Sigma), and periodic acid–Schiff (PAS) reagent (ab150680, Abcam, Cambridge, UK) according to the manufacturer’s instructions. After sealing with neutral gum, the sections were observed and photographed under an inverted microscope (IX73, Olympus, Tokyo, Japan). All morphological analyses were performed by two individual experienced pathologists in a double-blind manner.

### Immunohistochemical staining

The mouse cerebral cortex tissues were embedded in paraffin and sliced by ultra-thin microtome. The sections were dewaxed by xylene, rehydrated by graded alcohol and incubated with 3% hydrogen peroxide to block endogenous peroxidase activity. The slides were boiled in 10 mM sodium citrate (pH 6.0) for 30 min. Next, the sidles were blocked in the 10% normal goat serum for 15 min. The sections were incubated with antibody Fis1 (ab229969, 1:500, Abcam) and Drp1 (ab184247, 1:500, Abcam) at 4 ℃ in a wet room. The next day, the slides were washed with phosphate buffered saline (PBS), followed by incubation with the secondary goat anti-rabbit antibody (ab6721, 1:1000, Abcam) for 1 h. The immune reactivity was detected with diaminobenzidine (DAB) Kit (Invitrogen, Carlsbad, CA). Five different areas were photographed under the inverted microscope (IX73, Olympus) and quantified by Image J software.

### Assessment of kidney function

Glomerular filtration rate (GFR) was measured using FITC-inulin clearance. Briefly, FITC-inulin (F3272, Sigma) was dissolved in 0.9% NaCl (5% w/v) and dialyzed with a 1000-kDa dialysis membrane (Spectrum Laboratories) in 0.9% NaCl in the dark for 24 h at room temperature. Next, the dialyzed products were filtered under sterile conditions through a 0.22 μm filter (ThermoFisher). Then the mice were retro-orbitally injected with FITC-inulin (2 μL/g body weight) after anesthesia by isoflurane. Blood was collected in heparin-coated capillary tubes using a ~ 1-mm tail snip at 3rd, 5th, 7th, 10th, 15th, 35th, 56th, and 75th min following the injection. The blood was centrifuged and the plasma was diluted at a ratio of 1:20. Finally, the fluorescence was detected using a BioTek Synergy II plate reader and GFR was calculated using a two-phase exponential decay equation in GraphPad Prism.

### Biochemical analysis

Biochemical analysis was performed on blood samples collected from the mice fasted for 8–12 h at week 0 and 6. The blood was centrifuged at 1500*g* to isolate serum, which was preserved at -80. The contents of BUN (EIABUN, Invitrogen), creatinine (ab65340, Abcam), calcium (ab102505, Abcam) and phosphorus (C006, NanJing JianCheng Bioengineering Institute, Nanjing, China) were measured according to the corresponding instructions.

### Immunofluorescence

The mouse brain tissues were fixed in 4% paraformaldehyde, and were sliced into sections (40 μm) using the oscillating knife (VT1200s, Leica, Wetzlar, Germany). After antigen repair with sodium citrate buffer, the sections were cultured with 0.1% Triton X-100 for 10 min and sealed with 10% normal donkey serum. Subsequently, the sections were incubated with the primary antibody rabbit anti-NeuN (ab104225, 1:100, Abcam) at 4 ℃ overnight. The next day, the sections were washed with PBS and incubated with the secondary antibody Alexa fluor 488-coupled goat anti-rabbit IgG (ab150077, 1:200, Abcam) for 1 h at room temperature. After PBS washes, the nuclei were stained with 4′, 6-diamidino-2-phenylindole (DAPI; C1006, Beyotime). Five different visual fields were photographed under the FV-1000/ES confocal microscope and qualified by Image J software.

### Isolation, culture, and transduction of primary mouse cortical neurons

The primary mouse cortical neurons were isolated from C57BL/6J male mice (aged 1 day). The cerebral cortex was separated and the meninges and blood vessels were carefully removed. The cerebral cortex tissues were chopped and digested with 0.25% trypsin (25-200–072, Gibco, Carlsbad, CA) at 37 °C for 20 min, followed by gentle grinding. Subsequently, cells were seeded onto the 6-well plates coated with polylysine (100 μg/mL) and cultured with the neural basic medium (21103–049, Gibco) in the incubator with 5% CO_2_ at 37 °C. After 8 h, the medium was renewed every other day and a half. NeuN immunofluorescence staining was performed to ensure over 90% purity of the cultured neurons. Neurons were treated with 100 μM antioxidant butylhydroxyanisole (BHA; 78943, Sigma) and cultured in the medium at 37 °C for 2 h.

For transduction of primary mouse cortical neurons, pLKD-Ubc-eGFP-U6 vector was used for gene silencing and pLenti-Ubc-EGFP used for gene overexpression, both of which were purchased from OBiO Technology (Shanghai) Corp., Ltd. (Shanghai, China). According to the mouse gene sequence, the silencing and overexpression sequences were cloned into the corresponding vectors, which were co-transfected with the helper plasmids (psPAX2 and Vsvg) into 293T cells to package the lentivirus. The lentiviruses were harvested, filtered, and concentrated. The primary mouse cortical neurons were transduced with vector or lentivirus overexpressing Drp1. Neurons harvested after 48 h transduction for 48 h were used for the following experiments.

### Dihydroethidium (DHE) staining

The reactive oxygen species (ROS) levels in mouse cortical tissues and primary cortical neurons were detected using DHE kit (S0063, Beyotime) according to the instructions. The 2 μM DHE was incubated with frozen mouse cortical sections or primary cortical neurons at 37 °C for 30 min for fluorescent probe loading, followed by washing. At last, five different visual fields were photographed under the FV-1000/ES confocal microscope and qualified by Image J software.

### Terminal deoxyribonucleotidyl transferase (TdT)-mediated biotin-16-dUTP nick-end labeling (TUNEL) assay

The apoptosis in mouse cortical tissues was detected according to the instructions of the Kit (C1088, Beyotime). Frozen mouse cortical sections were fixed with 4% paraformaldehyde for 30–60 min and washed with PBS three times. The sections were incubated with 0.5% Triton X-100 in PBS at room temperature for 5 min and washed with PBS two times. Subsequently, the sections were incubated with 50 μL TUNEL solution at 37 °C avoiding light for 60 min and washed with PBS three times. The sections were sealed with anti-fluorescence quenching liquid. At last, five different visual fields were photographed under the FV-1000/ES confocal microscope and qualified by Image J software.

### Measurement of superoxide dismutase (SOD) activity and contents of malondaldehyde (MDA) and adenosine triphosphate (ATP)

The SOD activity and contents of MDA and ATP were measured using the Superoxide Dismutase Activity Assay Kit (ab65354, Abcam), Lipid Peroxidation (MDA) Assay Kit (ab233471, Abcam, UK), and ATP Assay Kit (ab83355, Abcam), respectively.

### Transmission electron microscope (TEM)

Mouse cerebral cortical tissue sections were fixed with 2.5% glutaraldehyde at 4 °C for 24 h, rinsed with PBS three times, fixed with 1% osmium tetroxide at 4 °C for 2 h, and rinsed with PBS three times again. Next, the tissues were dehydrated in 30%, 50%, 70%, 90%, and 100% ethanol for 30 min, respectively. After embedding in Epon-812 mixture overnight, the tissues were sliced into sections (50–60 nm). The sections were photographed under the TEM (H-7650, Hitachi, Tokyo, Japan). The ultrastructure of mitochondria in each section was analyzed using the Image J software.

### Reverse transcription quantitative polymerase chain reaction (RT-qPCR)

The total RNA was isolated from tissues or cells using TRIzol (16096020, Invitrogen). The mRNA was reversely transcribed into cDNA by using PrimeScript reverse transcriptase kit (RR047A, Takara Bio Inc., Otsu, Shiga, Japan). RT-qPCR was conducted according to the instructions of TaqMan Gene Expression Assays protocol (Applied Biosystems, Foster City, CA, USA). Three replicates were set for RT-qPCR. The primer sequences (Additional file 2: Table S1) were designed based on the NCBI (https://www.ncbi.nlm.nih.gov/tools/primer-blast/). The 2^−ΔΔCt^ method was used to quantify the relative expression of target genes.

### Western blot analysis

The protein was extracted from mouse cerebral cortical tissues and primary cortical neurons using protease inhibitor-containing radio immunoprecipitation assay (RIPA) lysis buffer (Beyotime). The protein concentration was detected by bicinchoninic acid (BCA) kit (20201ES76, Yeasen Company, Shanghai, China). The protein was qualified according to different concentrations. The protein was separated by sodium dodecyl sulfate–polyacrylamide gel electrophoresis (SDS-PAGE), electrotransferred onto a polyvinylidene fluoride (PVDF) membrane (IPVH85R, Millipore, Germany). The membrane was blocked with 5% bovine serum albumin (BSA) for 1 h, and then incubated with the prepared primary antibodies Drp1 (ab184247, 1:1000, Abcam), EZH2(ab186006, 1:1000, Abcam), HIF-1ɑ (36169, 1:1000, Cell Signaling Technologies, Beverly, MA, USA), and GAPDH (ab8245, 1:5000, Abcam) at 4 °C overnight. Subsequently, the membrane was washed by Tris-buffered saline with Tween (TBST) three times, 5 min per time, followed by 1-h incubation with the horseradish peroxidase (HRP)-marked secondary antibody goat anti-rabbit IgG (ab6721, Abcam) or goat anti-mouse IgG (ab6789, Abcam) diluted at 1:2000. After TBST rinses, the membrane was developed with luminescent liquid. The results were analyzed by the ImageJ software (National Institutes of Health, Bethesda, Maryland, USA). The relative protein expression was expressed as the ratio of the gray value of protein to be tested to that of internal reference (GAPDH).

### Chromatin immunoprecipitation (ChIP) assay

An EZ-Magna ChIP kit (Millipore) was used for the ChIP assay. Briefly, primary mouse cortical neurons were cross-linked and cultured with 1% formaldehyde for 10 min. The cross-linking was terminated with 125 mM glycine at room temperature for 5 min. After washing with prechilled PBS, the neurons were centrifuged at 2000*g* for 5 min, and suspended in cell lysis buffer to make final concentration of 2 × 10^6^ cells per 200 μL. The neurons were added with protease inhibitor mixture, centrifuged at 5000*g* for 5 min, and resuspended in nuclear separation buffer. After an ice bath for 10 min, the neurons were ultrasonically lysed to obtain 200 ~ 1000 bp chromatin fragments, followed by centrifugation at 14,000*g* at 4 °C for 10 min. The collected supernatant (100 μL, DNA fragment) was added with 900 μL ChIP Dilution Buffer, 20 μL 50 × PIC, and 60 μL Protein A agarose/salmon sperm DNA, rotated transversely, and mixed evenly at 4℃ for 1 h. After standing at 4 °C for 10 min, the neurons were centrifuged at 700*g* for 1 min. The supernatant (20 μL) was used as Input. The supernatant in the experiment group was incubated with 1 μL rabbit HIF-1α antibody (36169, CST), and that in the NC group was incubated with 1 μL rabbit anti-IgG (ab172730, Abcam). Each tube was added with 60 μL Protein A agarose/salmon sperm DNA and rotated transversely at 4℃ for 2 h. After standing for 10 min, the cells were centrifuged at 700*g* for 1 min to remove supernatant. The precipitate was washed with 1 mL low-salt buffer, high-salt buffer, LiCl solution, and TE (twice). Each tube was washed with 250 μL ChIP Wash Buffer two times. The cross-linking was reversed with 20 μL 5 M NaCl. The enriched chromatin fragments in the harvested were detected by RT-qPCR. The primers for EZH2 promoter are shown as F: 5′-CTGGAGCTCGCTCCGAAGG-3′, R: 5′-CAACCCAATCGCCATCGCT-3′.

### Dual-luciferase reporter gene assay

The dual-luciferase reporter gene plasmids containing EZH2 promoter WT and promoter Mut (mutation of binding site with HIF-1ɑ) were constructed, respectively. The reporter plasmids and other related plasmids were co-transfected into mouse primary cortical neurons, which were lysed 48 h after transfection. The cells were centrifuged at 12,000*g* for 1 min to collect supernatant. Dual-Luciferase® Reporter Assay System (E1910, Promega, Madison, WI, USA) was utilized to detect luciferase activity.

### Statistical analysis

All data were analyzed by GraphPad Prism. Measurement data were expressed as mean ± standard deviation. Data comparison between two groups was carried out by unpaired *t-*test, and that among multiple groups was conducted by one-way analysis of variance (ANOVA). Data at different time points were compared using repeated measures ANOVA. A value of *p* < 0.05 was regarded statistically significant.

## Results

### Calcium content elevation in the prefrontal cortex and neuronal damage in CKD mice

**Fig. 1:**
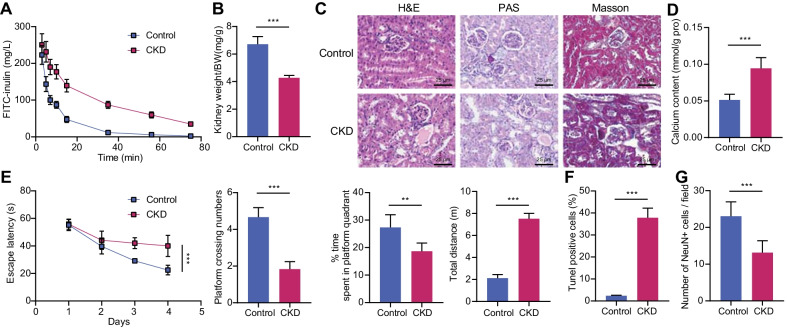
The increased calcium content in prefrontal cortical tissues of CKD mice leads to nerve injury. **A** FITC inulin clearance curve. **B** Mean mouse kidney weight. **C** Representative H&E, PAS, and Masson images of mouse kidney sections (scale bar = 50 μm). **D** The calcium content in mouse prefrontal cortical tissues. **E** The escape latency, the frequency of times entering the target quadrant, the time spent in the platform quadrant, and the total movement distance. **F** TUNEL-positive cells (apoptosis) in mouse prefrontal cortical tissues. G, Proportion of NeuN-positive cells in mouse prefrontal cortical tissues. *N* = 6. *p < 0.05; **p < 0.001

A CKD mouse model was established using an adenine-containing diet. After 6 weeks of adenine-containing diet, the mice developed with weight loss, poor mental state, polydipsia, and polyuria, corresponding to elevated levels of BUN, Creatinine, Ca^2+^, and Pi despite no death (Additional file 3: Table S2). In addition, the GFR decreased by about 70% in the mice fed an adenine-containing diet (Fig. 1A), and kidney volume and weight are significantly reduced, with uneven surface and pale color, forming an obvious “big white kidney” (Fig. 1B). The mice fed a normal diet showed normal renal tissue structure, intact renal tubular epithelial cells, and no significant pathological changes in glomerulus or renal interstitium, while the mice fed an adenine-containing diet displayed dilation of renal lumen and glomerular hypertrophy, accompanied by obvious fibrosis, edema of renal tubular epithelial cells, and interstitial inflammatory infiltration (Fig. 1C). These findings suggested the successful construction of a CKD mouse model.

Moreover, it was found that the content of calcium in prefrontal cortical tissues increased (Fig. 1D). Also, prolonged latency of escape, reduced frequency of entering the target quadrant, shortened time of staying in the platform quadrant, and increased total movement distance were detected in the mice with CKD (Fig. 1E). TUNEL and immunofluorescence assays displayed increased apoptotic cells (Fig. 1F, Additional file 1: Fig. S1A) and reduced NeuN-positive cells (neurons) in the prefrontal cortical tissues of mice with CKD (Fig. 1G, Additional file 1: Fig. S1B).

These data suggested increased calcium levels in prefrontal cortex and neurological impairment of mice with CKD.

### Significance of Drp1/Fis1 axis in mitochondrial fragmentation and dysfunction in the cerebral cortex of CKD mice

**Fig. 2:**
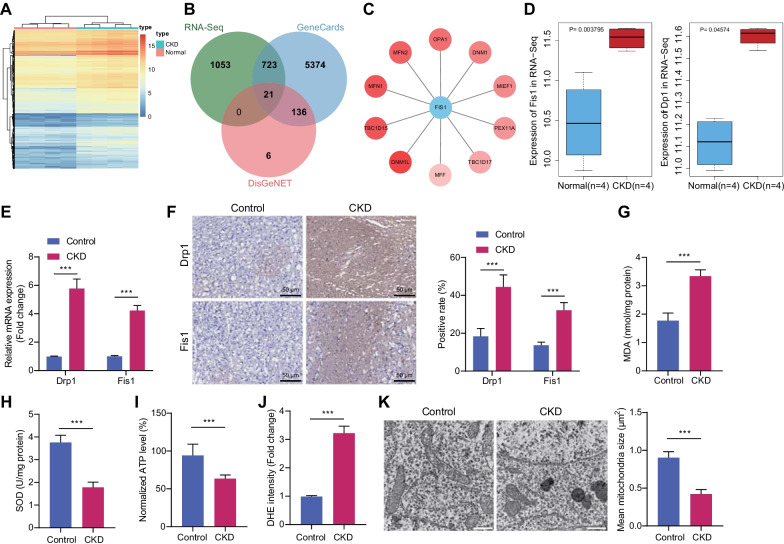
Drp1/Fis1 axis accelerates mitochondrial dysfunction in the cerebral cortex of CKD mice. **A** Heat map of expression of upregulated genes in RNA-Seq. **B** Venn map of intersection between upregulated genes in RNA-Seq and “Renal Failure”-related genes in GeneCards and DisGeNET. **C** The genes interacted with Fis1 predicted by STRING. The redder the circle where the gene located indicates the higher combined_score. Drp1 (DNM1L) has the highest combined_score. **D** FIS1 and Drp1 expression in RNA-Seq cerebral cortex sample of normal mice (blue, *n* = 3) and CKD mice (red, *n* = 3). **E** mRNA levels of Fis1 and Drp1 in the cerebral cortex of CKD mice determined by RT-qPCR. **F** Expression of Fis1 and Drp1 proteins in the cerebral cortex of CKD mice detected by immunohistochemical staining. **G**–**J** MDA level (**G**), SOD activity (**H**), ATP level (**I**), and ROS level (**J**) in the cerebral cortex of CKD mice. **K** The morphology of mitochondria in cerebral cortex of CKD mice observed under the TEM (scale bar = 1 μm) and mean mitochondria size. *N* = 6. *p < 0.05

In order to explore the molecular mechanism of neuronal damage in mice with CKD, the cerebral cortex of mice was isolated for RNA-Seq analysis, and 1797 upregulated genes were obtained (Fig. 2A). Subsequently, 6254 and 163 “Renal Failure”-related genes were obtained from GeneCards and DisGeNET, respectively, and 21 candidate genes were detected in the intersection of these two sets (Fig. 2B). Among them, Fis1 exhibited the largest fold change (Additional file 4: Table S3).

Upstream genes interacted with Fis1 were then predicted through STRING tool. Drp1 showed the highest interaction score (0.985) among the top 10 genes interacted with Fis1 (Fig. 2C). According to the sequencing results, both Fis1 and drp1 were upregulated genes in the cerebral cortex of CKD mice (Fig. 2D).

RT-qPCR and immunohistochemical staining consistently presented increased expression of Drp1 and Fis1 in the cerebral cortex of CKD mice (Fig. 2E, F). Additionally, levels of MDA and ROS increased, and SOD activity and ATP level decreased in the cerebral cortex of CKD mice (Fig. 2G–J, Additional file 1: Fig. S1C). TEM displayed that mitochondria in the cerebral cortex of normal mice were significantly prolonged and arranged neatly, while mitochondrial fragmentation was visualized in the cerebral cortex of CKD mice (Fig. 2K).

It was thus concluded that the Drp1/Fis1 axis correlated with mitochondrial fragmentation and consequent mitochondrial dysfunction in the cerebral cortex of CKD mice.

### Contribution of hypercalcemia to neuronal damage in CKD through Drp1/Fis1-mediated mitochondrial fragmentations

**Fig. 3:**
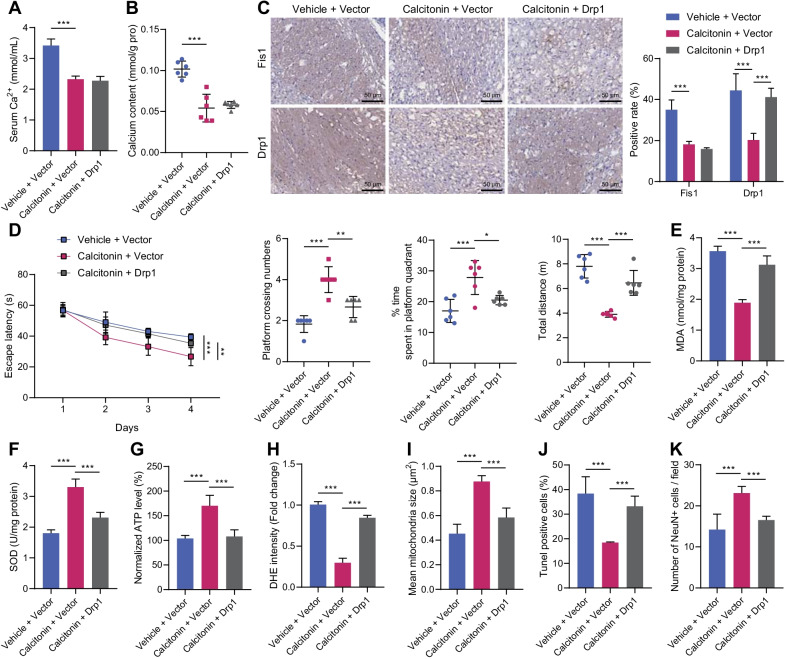
CKD-induced hypercalcemia mediates Drp1/Fis1 to promote mitochondrial fragmentation and aggravate nerve damage. CKD mice were treated with calcitonin alone or combined with overexpressed Drp1 (*n* = 6). **A** Ca^2+^ content in serum of CKD mice. **B** Ca^2+^ content in prefrontal cortical tissues of CKD mice. **C** Expression of Drp1 and Fis1 proteins in prefrontal cortical tissues of CKD mice detected by immunohistochemical staining. **D** The escape latency, the number of times entering the target quadrant, the time spent in the platform quadrant, and the total distance of action of CKD mice by Morris water maze test. **E**–**H** MDA level (**E**), SOD activity (**F**), ATP level (**G**), and ROS level (**H**) in the prefrontal cortical tissues of CKD mice. **I** The mean mitochondria size in cerebral cortex of CKD mice. **J** Apoptosis in prefrontal cortical tissues of CKD mice detected by TUNEL staining. **K** Proportion of NeuN-positive cells in prefrontal cortical tissues of CKD mice detected by immunofluorescence staining. *N* = 6. *p < 0.05

CKD mice were treated with Calcitonin to alleviate hypercalcemia to verify whether Drp1/Fis1-mediated mitochondrial fragmentation involved in neuronal damage caused by CKD-induced hypercalcemia. Calcitonin treatment effectively reduced the Ca^2+^ content in the serum samples and prefrontal cortical tissues of CKD mice, while overexpression of Drp1 did not affect the suppressive effect of calcitonin on the Ca^2+^ content in serum and cortical tissues (Fig. 3A, B). However, calcitonin downregulated the levels of Drp1 and Fis1 in the prefrontal cortical tissues of CKD mice, while the Drp1 expression was rescued following infection with AAV expressing Drp1 without affecting that of Fis1 (Fig. 3C).

Next, we found that calcitonin treatment could shorten the escape latency, increase the frequency of entering the target quadrant and time of staying in the platform quadrant, and reduce the total movement distance in the mice with CKD; whereas, overexpression of Drp1 significantly counteracted the promotive effect of calcitonin on learning and memory functions in CKD mice (Fig. 3D). In addition, calcitonin reduced MDA and ROS levels, but elevated SOD and ATP activities in the cerebral cortex of CKD mice, while upregulated Drp1 significantly reversed the effect of calcitonin on mitochondria in cerebral cortex of CKD mice (Fig. 3E–I). TUNEL assay and immunofluorescence staining displayed that after calcitonin treatment, apoptosis was inhibited and NeuN-positive cells (neurons) elevated in the prefrontal cortical tissues of CKD mice, while restoration of Drp1 expression reversed these changes (Fig. 3J, K).

The obtained data indicated that hypercalcemia aggravated neuronal damage via Drp1/Fis1-mediated mitochondrial fragmentation in CKD.

### Drp1 upregulates EZH2 by enhancing its promoter activity

**Fig. 4:**
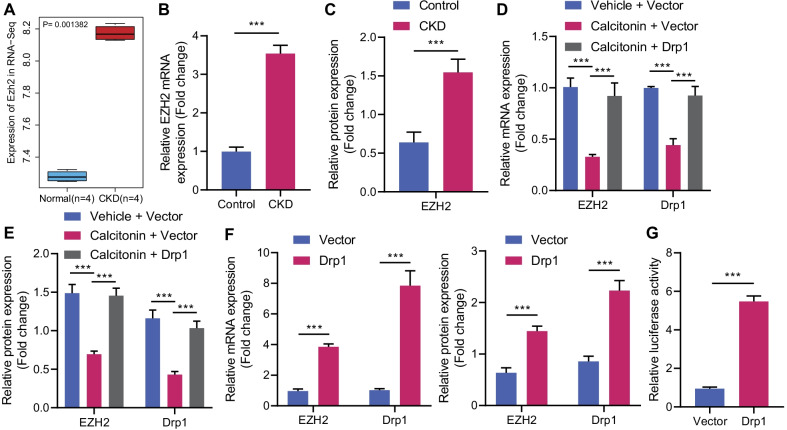
Promotive effect of Drp1 on EZH2 promoter activity and expression. **A** EZH2 expression in RNA-Seq cerebral cortex sample of normal mice (blue, *n* = 3) and CKD mice (red, *n* = 3). **B** EZH2 mRNA level in prefrontal cortical tissues of CKD mice determined by RT-qPCR. **C** EZH2 protein level in prefrontal cortical tissues of CKD mice measured by Western blot analysis. CKD mice were treated with calcitonin alone or combined with overexpressed Drp1 (*n* = 6). **D** EZH2 mRNA level in prefrontal cortical tissues of CKD mice determined by RT-qPCR. **E** Protein levels of Drp1 and EZH2 prefrontal cortical tissues of CKD mice measured by Western blot analysis. Mouse primary cortical neurons were transduced with lentivirus overexpressing Drp1. **F** mRNA levels of Drp1 and EZH2 in mouse primary cortical neurons determined by RT-qPCR. **G** EZH2 promoter activity detected by dual-luciferase reporter gene assay. *N* = 6 for mice, and *n* = 3 for cells. *p < 0.05

It has been reported that inhibition of EZH2 could relieve acute kidney injury [18]. In this study, EZH2 existed in the renal failure-related candidate genes (Fig. 2B). Meanwhile, RNA-seq displayed elevated EZH2 expression in the cerebral cortex of CKD mice (Fig. 4A). We experimentally determined significant increased mRNA and protein levels of EZH2 in the cerebral cortex of CKD mice (Fig. 4B, C). However, calcitonin treatment reduced the EZH2 expression in cerebral cortex of CKD mice, which effect was reversed by overexpressed Drp1 (Fig. 4D, E).

In addition, mouse primary cortical neurons were transduced with lentivirus overexpressing Drp1 and its corresponding control. Accordingly, upregulation of Drp1 elevated the EZH2 mRNA and protein levels (Fig. 4F) and augmented the EZH2 promoter activity (Fig. 4G). Hence, Drp1 could promote EZH2 promoter activity to increase its expression.

### Drp1 promotes the binding of HIF-1α to EZH2 promoter by inducing ROS production

**Fig. 5:**
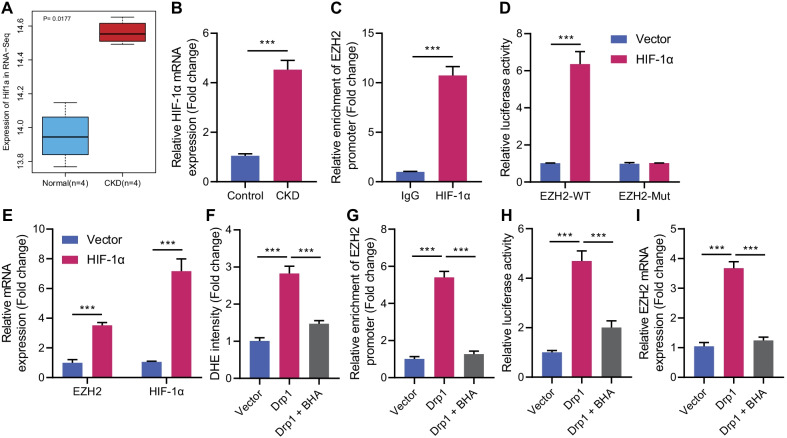
Drp1 affects EZH2 via ROS/HIF-1α. **A** HIF-1α expression in RNA-Seq cerebral cortex sample of normal mice (blue, *n* = 3) and CKD mice (red, *n* = 3). **B** HIF-1α level in prefrontal cortical tissues of CKD mice determined by RT-qPCR. Mouse primary cortical neurons were treated with HIF-1α. **C** Enrichment of HIF-1α on EZH2 promoter in mouse primary cortical neurons detected by ChIP assay. **D** The relationship between HIF-1α and EZH2 verified by dual-luciferase reporter gene assay. **E** Levels of HIF-1α and EZH2 in mouse primary cortical neurons determined by RT-qPCR. Mouse primary cortical neurons were transduced with lentivirus overexpressing Drp1 and treated with antioxidant BHA. **F** ROS level in mouse primary cortical neurons measured by DHE staining. **G** Enrichment of HIF-1α on EZH2 promoter in mouse primary cortical neurons detected by ChIP assay. **H** The relationship between HIF-1α and EZH2 verified by dual-luciferase reporter gene assay. **I** EZH2 mRNA levels in mouse primary cortical neurons determined by RT-qPCR. *N* = 6 for mice, and *n* = 3 for cells. *p < 0.05

Drp1-induced mitochondrial fission highly correlates with mitochondrial ROS generation [19], while the mitochondrial production of ROS contributes to HIF-1 activation [20]. A recent study has identified that ROS can facilitate the binding of HIF-1α to EZH2 promoter [21]. RNA-seq and RT-qPCR exhibited an elevation in HIF-1α expression in the cerebral cortex of CKD mice (Fig. 5A, sB), corresponding to increased ROS generation (Fig. 2G–J). It was speculated that Drp1 might promote the binding of HIF-1α to EZH2 promoter to increase EZH2 expression via regulating ROS production.

To validate this conjecture, the binding site between HIF-1α and EZH2 promoter was obtained from JASPAR website, and site with the highest score was (5′-GGACGGGC-3′) (Additional file 5: Table S4). Next, results of ChIP assay in this study revealed that transcription factor HIF-1α was abundantly enriched in EZH2 promoter region in mouse primary cortical neurons (Fig. 5C), and the luciferase assay verified that luciferase activity of EZH2-promoter WT and EZH2 mRNA level were enhanced by overexpressed HIF-1α, while no evident difference was found in EZH2-promoter Mut (Fig. 5D, E). These findings supported the conclusion that HIF-1α could bind to EZH2 promoter to promote EZH2 expression.

As shown in Fig. 5F–H and Additional file 1: Fig. S1D, treatment with antioxidant BHA inhibited the increase of ROS, enrichment of HIF-1α on EZH2 promoter, and luciferase activity of EZH2 promoter caused by overexpression of Drp1. In addition, BHA also significantly reduced the expression of EZH2 induced by Drp1 (Fig. 5I). These data collectively demonstrated that Drp1 promoted the binding of HIF-1α and EZH2 to upregulate EZH2 by inducing ROS generation.

### Silencing of Drp1 alleviates neuronal damage via ROS/HIF-1α/EZH2 axis following CKD in vivo

**Fig. 6:**
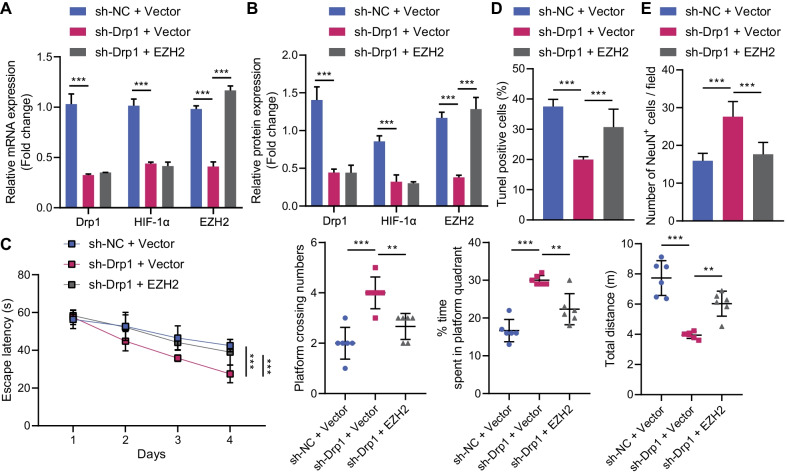
Drp1 aggravates hypercalcemia-induced nerve injury by regulating ROS/HIF-1α/EZH2 in CKD mice. CKD mice were injected with the corresponding adeno-associated viruses (sh-NC + Vector, sh-Drp1 + Vector, and sh-Drp1 + EZH2). **A**, **B** mRNA levels and protein levels of Drp1, EZH2, and HIF-1α in prefrontal cortical tissues of CKD mice. **C** The escape latency, the frequency of times entering the target quadrant, the time spent in the platform quadrant, and the total movement distance of CKD mice by Morris water maze test. **D** Apoptosis in prefrontal cortical tissues of CKD mice. E, Proportion of NeuN-positive cells in prefrontal cortical tissues of CKD mice detected by immunofluorescence staining. *N* = 6. *p < 0.05

At last, CKD mice were injected with the corresponding AAV (sh-NC + Vector, sh-Drp1 + Vector, and sh-Drp1 + EZH2) to explore whether Drp1 could regulate the ROS/HIF-1α/EZH2 axis to alleviate neurological impairment induced by hypercalcemia in CKD. Drp1 knockdown reduced the expression of EZH2 in the cerebral cortex of CKD mice, which could be rescued by manipulation with AAV2 expressing EZH2; whereas, HIF-1ɑ protein level remained unaffected by restoration of EZH2 in the presence of Drp1 (Fig. 6A, B).

Moreover, silencing of Drp1 enhanced learning and memory abilities of CKD mice, while EZH2 overexpression counteracted this effect (Fig. 6C). TUNEL assay and immunofluorescence staining displayed that downregulated Drp1 inhibited apoptosis and increased NeuN-positive cells (neurons) in the prefrontal cortical tissues of CKD mice, while upregulated EZH2 reversed these effects (Fig. 6D, E, Additional file 1: Fig. S1E, F).

These findings demonstrated that Drp1 knockdown could attenuate neuronal damage via the ROS/HIF-1α/EZH2 in CKD mice.

## Discussion

CKD is a main cause of mortality and morbidity worldwide, which causes a great burden on quality of life and economy [22]. Disordered mineral homeostasis is one of the prominent hallmarks of CKD, including Ca^2+^ and phosphorus (P_i_) deregulation [23]. Another study has also demonstrated that hypercalcemia is associated with the pathogenesis of CKD [5]. It has been suggested that hypercalcemia may cause neurological symptomatology in the setting of posterior reversible encephalopathy syndrome [24], and that hypercalcemia can also deteriorate physical and cognitive function in the setting of Huntington's disease [24]. The current study also exhibited increased calcium in the brain tissues of CKD mice and the contribution of which to neurological impairment following CKD. Data obtained in our study demonstrated that silencing of Drp1 exerted inhibitory properties in mitochondrial fragmentation to attenuate hypercalcemia-induced neurological impairment in CKD through the inactivation of ROS/HIF-1α/EZH2 axis.

The present study demonstrated that Drp1 and Fis1 were upregulated in the cerebral cortex of CKD mice. Consistently, Drp1 expression is found to be elevated in the mice rendered with chronic renal failure [25]. A recent study has revealed that downregulation of Drp1 could maintain mitochondrial structure and inhibit oxidative stress to protect against progressive kidney injury [26]. Drp1/Fis1 axis was indicated by experimental data in this study to promote mitochondrial fragmentation to induce mitochondrial dysfunction in the cerebral cortex of CKD mice by increasing levels of MDA and ROS and reducing the activities of SOD and ATP. It is known that mitochondrion homeostasis plays a vital role in renal function and mitochondria promote the ATP production in the kidney [11]. SOD, as a well-known antioxidant enzyme, is an indicator of impaired ROS production, which exerts a crucial function in the pathogenesis of CKD and acute kidney injury [27]. Fis1 could bind to the mitochondrial outer membrane through a transmembrane region located in its C-terminal region, and elevated expression of Fis1 enhances mitochondrial fragmentation in the kidney [28]. Also, Drp1 constitutively modulates mitochondrial dynamics and further affects the growth of cortical neurons [29]. It has attracted great attention that inhibition of Drp1/Fis1-mediated pathological mitochondrial fission attenuates the neuronal injury through inhibiting neuronal cell death and supporting neuronal cell survival [30]. In a transgenic mouse model of Parkinson's disease, Drp1 function alterations and mitochondrial morphological changes are correlated with ɑ-synuclein-associated pathology; it is also suggested that Drp1 loss-of-function in the mitochondria can lead to enlargement of neuronal mitochondria [29]. Our findings were consistent with these findings and additionally added evidence suggesting that suppression of Drp1/Fis1-mediated mitochondrial fragmentation attenuated hypercalcemia-induced neuronal injury following CKD.

Moreover, the obtained data in the present study identified that Drp1 could promote HIF-1α binding to EZH2 promoter by inducing ROS production, which might be one of mechanisms responsible for aggravated neuronal injury induced by hypercalcemia in CKD mice. Excessive mitochondrial ROS production highly correlates with mitochondrial fragmentation and mitochondrial dysfunction [31, 32]. Multiple evidences have proved that Drp1-induced mitochondrial fragmentation results in mitochondrial ROS generation [33, 34]. Existing literature also reveals that increased ROS level is involved in the pathogenesis of CKD [35, 36]. Additionally, a prior study has verified that the generation of ROS leads to HIF-1 activation [20]. A prior study has reported correlations of HIF-1α activation with mitochondrial dysfunction and oxidative stress [37]. Furthermore, it is interesting to note that ROS generation contributes to activation of HIF-1α by promoting binding of HIF-1α to the EZH2 promoter, which resulted in increased EZH2 expression [21], which is consistent with our findings. EZH2, as a histone-lysine N-methyltransferase enzyme that catalyzes the addition of methyl groups to histone H3 at lysine 27, is an inducer of gene silencing. An existing has revealed the protective effect induced by pharmacological and/or genetic loss of EZH2 against the AKI [38]. A recent study has also proved that EZH2 inhibition could relieve hyperuricemia-induced renal injury and fibrosis, suggesting its promise as a potential strategy for the treatment of hyperuricemia-induced CKD [39]. Additionally, an inhibitor of GSK-126 exerts protective effect on CA1 neurons from H3K27me3-mediated apoptosis after cerebral ischemia [40]. These findings partially support the notion that Drp1 aggravates hypercalcemia-induced neuronal injury following CKD by triggering mitochondrial fragmentation via activation of ROS/HIF-1α/EZH2 axis, yet whether GSK-126 and other EZH2 inhibitors have neuroprotective effects on CKD requires additional investigation. Furthermore, CaMKII is a key effector that controls acetaldehyde-evoked Drp1 phosphorylation and mitochondrial impairment in cerebral cortical neurons [41]. Mitochondrial-localized protein phosphatase 2A (PP2A) regulatory subunit Bβ2, which shows neuroprotective activity against stroke, is regarded as a neuron-specific activator of Drp1 [42]. Additionally, CDK5-mediated phosphorylation of Drp1 regulates Abeta(1-42)-induced mitochondrial fission and neuronal apoptosis [43]. Ca2+/calcineurin also mediates the dephosphorylation of Drp1 at serine 637, thereby exacerbating endoplasmic reticulum stress and endoplasmic reticulum-mediated mitochondrial fission [44]. It is possible that hypercalcemia may induce the expression of Drp1 and Fis1 by regulating key factors such as CaMKII, PP2A regulatory subunit Bβ2, CDK5, and calcineurin, which shall be excavated in the following experiments.

## Conclusions

**Fig. 7:**
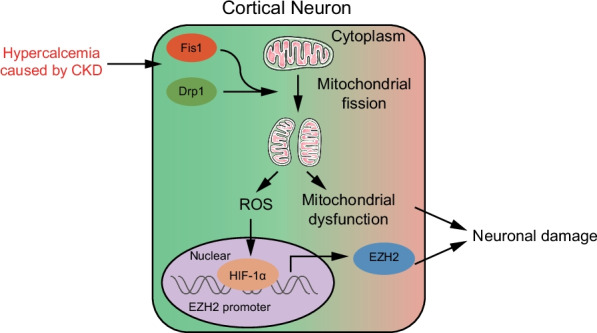
Molecular mechanism of Drp1 in hypercalcemia-induced nerve function damage following renal failure

To sum up, our study demonstrated that silencing of Drp1 inhibited ROS/HIF-1α/EZH2 axis, all of which leads to the alleviation of hypercalcemia-induced neuronal injury following CKD (Fig. 7). Our findings pave way for the development of neuroprotective targets for the treatment of CKD. However, the interactions among Drp1, ROS, HIF-1α, EZH2, as well as their involvement in mitochondrial fragmentation and hypercalcemia-induced neuronal injury in the progression of CKD need to be explored more deeply.

## Supplementary Information

**Additional file 1: Figure S1.** Representative TUNEL, immunofluorescence staining, and DHE staining images. A, Representative images showing TUNEL-positive cells in prefrontal cortical tissues of control and CKD mice; B, Representative immunofluorescence staining images showing NeuN-positive cells in prefrontal cortical tissues of control and CKD mice; C, Representative DHE staining images showing ROS level in prefrontal cortical tissues of control and CKD mice; D, Representative DHE staining images showing ROS level in mouse primary cortical neurons; E, Representative images showing TUNEL-positive cells in prefrontal cortical tissues of CKD mice following treatments; F, Representative immunofluorescence staining images showing NeuN-positive cells in prefrontal cortical tissues of CKD mice following treatments.

**Additional file 2: Table S1.** Primer sequences of RT-qPCR (mouse).

**Additional file 3: Table S2.** Physiological parameters of control mice and mice with induced CKD.

**Additional file 4: Table S3.** The results of RNA-Seq analysis.

**Additional file 5: Table S4.** Binding sites between HIF1A and EZH2_Promoter.

## Data Availability

The data that support the findings of this study are available on request from the corresponding author.

## Abbreviations

CKD: Chronic kidney disease
ChIP: Chromatin immunoprecipitation
ESRD: End-stage renal disease
Drp1: Dynamin-related protein 1
BUN: Blood urea nitrogen
RNA-Seq: RNA-sequencing
H&E: Hematoxylin–eosin
PAS: Periodic acid–Schiff
PBS: Phosphate buffered saline
DAB: Diaminobenzidine
GFR: Glomerular filtration rate
DHE: Dihydroethidium
ROS: Reactive oxygen species
TdT: Terminal deoxyribonucleotidyl transferase
TUNEL: TdT-mediated biotin-16-dUTP nick-end labeling
SOD: Superoxide dismutase
MDA: Malonaldehyde
ATP: Adenosine triphosphate
TEM: Transmission electron microscope
RT-qPCR: Reverse transcription quantitative polymerase chain reaction
RIPA: Radio immunoprecipitation assay
BCA: Bicinchoninic acid
SDS-PAGE: Sodium dodecyl sulfate–polyacrylamide gel electrophoresis
PVDF: Polyvinylidene fluoride
BSA: Bovine serum albumin
TBST: Tris-buffered saline with Tween
HRP: Horseradish peroxidase
ChIP: Chromatin immunoprecipitation
ANOVA: Analysis of variance
PP2A: Protein phosphatase 2A
