# Supplementary material for: Drp1 activates ROS/HIF-1α/EZH2 and triggers mitochondrial fragmentation to deteriorate hypercalcemia-associated neuronal injury in mouse model of chronic kidney disease
Source: J Neuroinflammation. 2022 Sep 1;19:213. doi: 10.1186/s12974-022-02542-7 (PMC9438241; doi:10.1186/s12974-022-02542-7)
Supplement: Supplementary file 1 — Additional file 1: Figure S1. Representative TUNEL, immunofluorescence staining, and DHE staining images. A, Representative images showing TUNEL-positive cells in prefrontal cortical tissues of control and CKD mice; B, Representative immunofluorescence staining images showing NeuN-positive cells in prefrontal cortical tissues of control and CKD mice; C, Representative DHE staining images showing ROS level in prefrontal cortical tissues of control and CKD mice; D, Representative DHE staining images showing ROS level in mouse primary cortical neurons; E, Representative images showing TUNEL-positive cells in prefrontal cortical tissues of CKD mice following treatments; F, Representative immunofluorescence staining images showing NeuN-positive cells in prefrontal cortical tissues of CKD mice following treatments. [file 12974_2022_2542_MOESM1_ESM.docx]

**
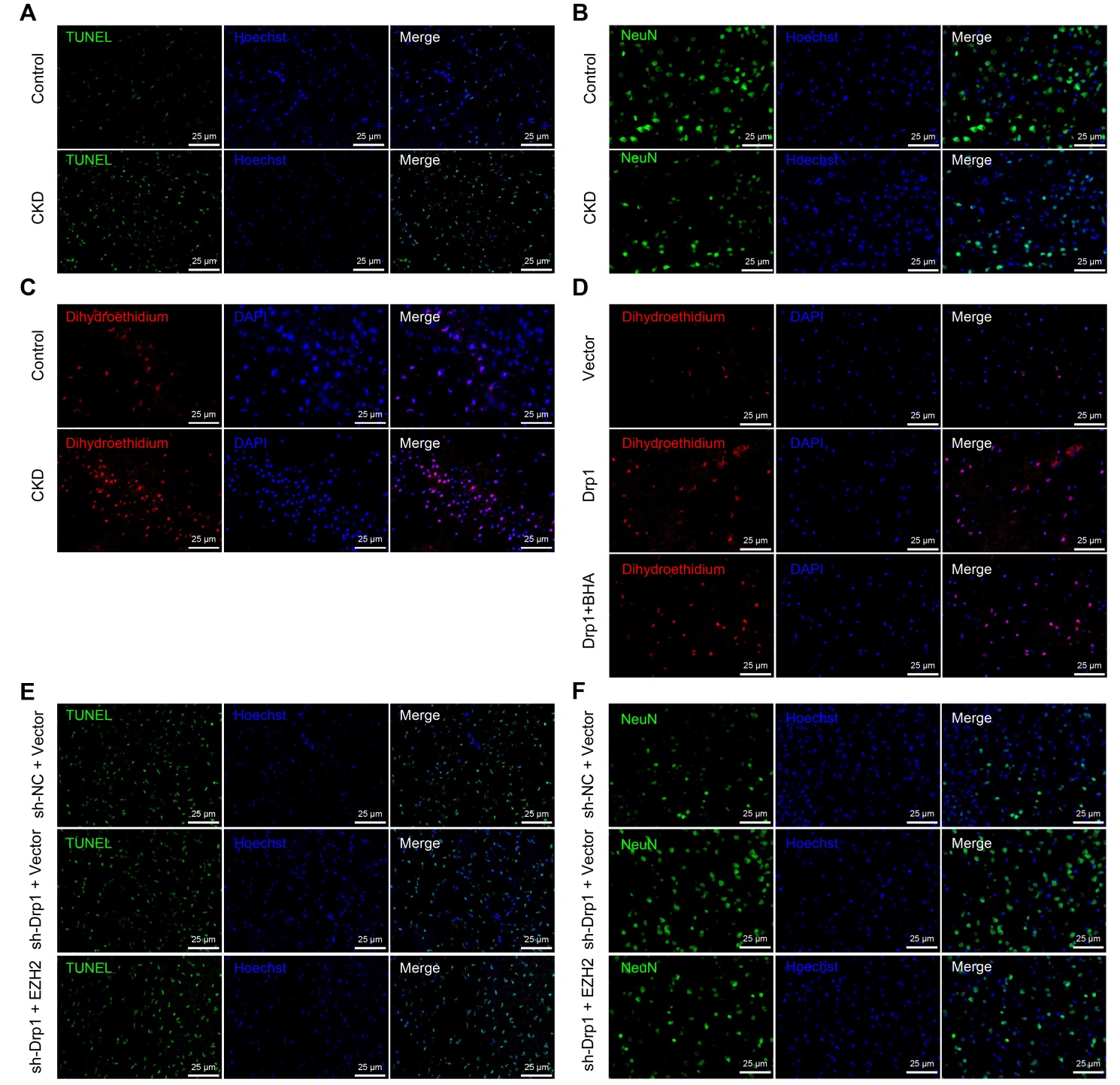
**

**Figure S1** Representative TUNEL, immunofluorescence staining, and DHE staining images. A, Representative images showing TUNEL-positive cells in prefrontal cortical tissues of control and CKD mice; B, Representative immunofluorescence staining images showing NeuN-positive cells in prefrontal cortical tissues of control and CKD mice; C, Representative DHE staining images showing ROS level in prefrontal cortical tissues of control and CKD mice; D, Representative DHE staining images showing ROS level in mouse primary cortical neurons; E, Representative images showing TUNEL-positive cells in prefrontal cortical tissues of CKD mice following treatments; F, Representative immunofluorescence staining images showing NeuN-positive cells in prefrontal cortical tissues of CKD mice following treatments.
