# Supplementary material for: Drp1 activates ROS/HIF-1α/EZH2 and triggers mitochondrial fragmentation to deteriorate hypercalcemia-associated neuronal injury in mouse model of chronic kidney disease
Source: J Neuroinflammation. 2022 Sep 1;19:213. doi: 10.1186/s12974-022-02542-7 (PMC9438241; doi:10.1186/s12974-022-02542-7)
Supplement: Supplementary file 2 — Additional file 2: Table S1. Primer sequences of RT-qPCR (mouse). [file 12974_2022_2542_MOESM2_ESM.docx]

**Table S1** Primer sequences of RT-qPCR (mouse)

| Genes | Sequences (5'-3') |
| --- | --- |
| Drp1 | F: GGACCCACTAGGTGGCCTTA |
|  | R: ACGCTTAATCTGACGTTTGACC |
| Fis1 | F: AGCTGGAACGCCTGATTGAT |
|  | R: TGGAGACAGCCAGTCCAATG |
| EZH2 | F: GTGACCACAGGATAGGCATCT |
|  | R: TCGTTCGATGCCCACATACT |
| GAPDH | F: AGGTCGGTGTGAACGGATTTG |
|  | R: GGGGTCGTTGATGGCAACA |
| HIF-1ɑ | F: AGCATACAGTGGCACTCACAG |
|  | R: AGGCTCCTTGGATGAGCTTTG |

Note: RT-qPCR, reverse transcription quantitative polymerase chain reaction; F, forward; R, reverse.
