# Supplementary material for: Drp1 activates ROS/HIF-1α/EZH2 and triggers mitochondrial fragmentation to deteriorate hypercalcemia-associated neuronal injury in mouse model of chronic kidney disease
Source: J Neuroinflammation. 2022 Sep 1;19:213. doi: 10.1186/s12974-022-02542-7 (PMC9438241; doi:10.1186/s12974-022-02542-7)
Supplement: Supplementary file 3 — Additional file 3: Table S2. Physiological parameters of control mice and mice with induced CKD. [file 12974_2022_2542_MOESM3_ESM.docx]

**Table S2** Physiological parameters of control mice and mice with induced CKD

| Parameters | Control | CKD |
| --- | --- | --- |
|  | (N = 6) | (N = 6) |
| Body weight (g) |  |  |
| 0 week | 25.8 ± 0.3 | 26.2 ± 0.4 |
| 6 weeks | 29.1 ± 0.5 | 20.1 ± 0.6* |
| Food intake (g/day) |  |  |
| 0 week | 5.1 ± 0.2 | 4.7 ± 0.2 |
| 6 weeks | 5.0 ± 0.2 | 3.6 ± 0.5* |
| Urinary volume (μL/24 h) |  |  |
| 0 week | 774 ± 33 | 589 ± 34 |
| 6 weeks | 673 ± 56 | 1605 ± 151* |
| Water intake (mL) |  |  |
| 0 week | 4.2 ± 0.2 | 4.3 ±  0.3 |
| 6 weeks | 4.1 ± 0.3 | 11.4 ± 0.6* |
| BUN (mg/dL) |  |  |
| 0 week | 23.4 ± 0.5 | 25.6 ± 0.6 |
| 6 weeks | 25.7 ± 0.2 | 62.6 ± 2.5* |
| Creatinine (mg/mL) |  |  |
| 0 week | 0.38 ± 0.03 | 0.36 ± 0.05 |
| 6 weeks | 0.34 ± 0.02 | 0.89 ± 0.04* |
| Serum Ca2+ (mmol/L) |  |  |
| 0 week | 2.2 ± 0.2 | 2.2 ± 0.3 |
| 6 weeks | 2.1 ± 0.1 | 2.9 ± 0.4* |
| Serum Pi (mmol/L) |  |  |
| 0 week | 1.8 ± 0.1 | 2.0± 0.4 |
| 6 weeks | 2.1 ± 0.3 | 2.8 ± 0.5* |

Note: Physiological measurements of mice subjected to adenine-enriched dietary (ADE) treatment for 6 weeks or control diet. Data indicated as mean ± standard deviation were collected after 24 h housing in individual metabolic cages. * *p* < 0.05 *vs.* control diet, Control, or CKD. N = 6.
