# Supplementary material for: Drp1 activates ROS/HIF-1α/EZH2 and triggers mitochondrial fragmentation to deteriorate hypercalcemia-associated neuronal injury in mouse model of chronic kidney disease
Source: J Neuroinflammation. 2022 Sep 1;19:213. doi: 10.1186/s12974-022-02542-7 (PMC9438241; doi:10.1186/s12974-022-02542-7)
Supplement: Supplementary file 4 — Additional file 4: Table S3. The results of RNA-Seq analysis. [file 12974_2022_2542_MOESM4_ESM.docx]

**Table S3** The results of RNA-Seq analysis

|  | logFC | logCPM | PValue | FDR |
| --- | --- | --- | --- | --- |
| Tfap2b | 3.728502 | 5.496941 | 4.56E-17 | 3.89E-14 |
| Ivl | 3.745071 | 4.975195 | 3.32E-15 | 1.60E-12 |
| Fcgbp | 4.793825 | 5.614949 | 1.71E-12 | 4.42E-10 |
| Pck1 | 3.695776 | 4.526374 | 4.33E-12 | 9.23E-10 |
| Dapl1 | 4.797286 | 4.087704 | 1.07E-11 | 2.08E-09 |
| Enpp2 | 2.917163 | 4.428484 | 8.75E-11 | 1.33E-08 |
| Smoc2 | 2.551361 | 5.872884 | 1.82E-10 | 2.53E-08 |
| Adgrf4 | 2.896352 | 4.729008 | 3.62E-10 | 4.72E-08 |
| Fgfr2 | 2.504733 | 6.330204 | 4.25E-10 | 5.45E-08 |
| Mill1 | 3.989036 | 3.395866 | 4.73E-10 | 5.76E-08 |
| Cfd | 2.391523 | 5.658971 | 4.78E-10 | 5.77E-08 |
| Gata3 | 3.419519 | 6.126363 | 4.96E-10 | 5.94E-08 |
| AABR07019383.1 | 9.52748 | 5.114641 | 5.13E-10 | 6.09E-08 |
| Crym | 5.26351 | 4.408983 | 5.32E-10 | 6.26E-08 |
| RGD1563334 | 5.428111 | 2.972318 | 7.65E-10 | 8.59E-08 |
| Scin | 2.606184 | 3.929245 | 1.38E-09 | 1.42E-07 |
| Mmp27 | 4.027843 | 3.154577 | 1.42E-09 | 1.44E-07 |
| Pik3c2g | 3.282896 | 3.753095 | 1.62E-09 | 1.63E-07 |
| AABR07010747.1 | 5.134876 | 2.319928 | 1.65E-09 | 1.63E-07 |
| Adh6a | 3.562485 | 2.624837 | 2.51E-09 | 2.41E-07 |
| Tmem229a | 3.811737 | 4.424989 | 3.65E-09 | 3.22E-07 |
| Fam25a | 2.766586 | 5.826216 | 3.93E-09 | 3.43E-07 |
| Sec14l4 | 3.522609 | 4.438481 | 4.33E-09 | 3.73E-07 |
| Fis1 | 9.849314 | 5.432415 | 4.77E-09 | 4.07E-07 |
| Gria2 | 3.311262 | 3.434007 | 4.80E-09 | 4.07E-07 |
| Mgst3 | 2.339475 | 4.77785 | 4.85E-09 | 4.09E-07 |
| Ace2 | 2.918864 | 4.322391 | 7.92E-09 | 6.34E-07 |
| LOC100302465 | 3.930163 | 2.689524 | 8.04E-09 | 6.40E-07 |
| Hrnr | 4.00173 | 5.611137 | 9.91E-09 | 7.55E-07 |
| RGD1565323 | 3.780012 | 3.505909 | 9.96E-09 | 7.56E-07 |
| Pof1b | 2.675414 | 6.50413 | 1.04E-08 | 7.81E-07 |
| Myoc | 2.327378 | 5.397937 | 1.28E-08 | 9.43E-07 |
| Calml3 | 3.562439 | 6.617101 | 1.43E-08 | 1.03E-06 |
| Perp | 2.951263 | 8.155152 | 1.59E-08 | 1.13E-06 |
| Nlrp10 | 3.284457 | 5.084033 | 1.72E-08 | 1.21E-06 |
| Bbox1 | 4.075712 | 2.201285 | 2.15E-08 | 1.47E-06 |
| Clca5 | 4.037509 | 7.257843 | 2.26E-08 | 1.54E-06 |
| LOC103695128 | 5.008252 | 5.059246 | 2.45E-08 | 1.66E-06 |
| Tyr | 8.187099 | 4.124987 | 2.89E-08 | 1.89E-06 |
| Casp14 | 3.375075 | 5.649404 | 3.14E-08 | 2.03E-06 |
| Krt23 | 2.898679 | 4.395364 | 3.51E-08 | 2.24E-06 |
| Pcdh20 | 3.64183 | 2.523793 | 3.58E-08 | 2.25E-06 |
| Dsg1 | 2.955529 | 8.334273 | 4.00E-08 | 2.44E-06 |
| Elovl3 | 3.913382 | 4.417815 | 4.72E-08 | 2.80E-06 |
| Ret | 2.631446 | 4.421788 | 5.32E-08 | 3.07E-06 |
| Cidec | 2.853696 | 3.034468 | 6.41E-08 | 3.59E-06 |
| Muc15 | 3.055446 | 3.142361 | 6.66E-08 | 3.69E-06 |
| Pou3f1 | 3.105717 | 4.44837 | 7.09E-08 | 3.88E-06 |
| PCOLCE2 | 2.789884 | 4.420307 | 7.13E-08 | 3.88E-06 |
| Serpinb5 | 2.597751 | 7.220709 | 7.54E-08 | 4.06E-06 |
| Lgals7 | 3.127073 | 7.267536 | 8.17E-08 | 4.32E-06 |
| Ces1d | 2.659732 | 4.442865 | 8.47E-08 | 4.43E-06 |
| Frem1 | 2.383686 | 4.265298 | 9.51E-08 | 4.94E-06 |
| Dhcr24 | 1.971522 | 7.511021 | 1.02E-07 | 5.30E-06 |
| Angptl1 | 2.555012 | 5.148638 | 1.21E-07 | 6.18E-06 |
| Cldn1 | 2.435645 | 6.118389 | 1.24E-07 | 6.30E-06 |
| Sema3d | 2.995073 | 4.962147 | 1.30E-07 | 6.50E-06 |
| Tacstd2 | 2.486676 | 5.741318 | 1.50E-07 | 7.38E-06 |
| Nrep | 2.145171 | 5.323943 | 1.60E-07 | 7.81E-06 |
| Tp63 | 2.453822 | 7.200268 | 2.00E-07 | 9.67E-06 |
| Fam83b | 1.982024 | 5.627052 | 2.59E-07 | 1.22E-05 |
| Aadacl4 | 5.723577 | 1.824191 | 2.72E-07 | 1.27E-05 |
| Camsap3 | 2.383469 | 5.135381 | 2.91E-07 | 1.34E-05 |
| RGD1561998 | 3.894004 | 3.271905 | 3.04E-07 | 1.38E-05 |
| Cd109 | 2.29651 | 4.699182 | 3.03E-07 | 1.38E-05 |
| Acaa2 | 1.858743 | 5.741256 | 3.78E-07 | 1.68E-05 |
| Lipm | 2.100112 | 5.875775 | 3.83E-07 | 1.69E-05 |
| Mcpt1l1 | 2.473121 | 6.399776 | 4.14E-07 | 1.81E-05 |
| Col4a6 | 3.105538 | 2.862377 | 4.18E-07 | 1.81E-05 |
| Ehhadh | 2.625065 | 3.399018 | 4.26E-07 | 1.83E-05 |
| Acpp | 2.332323 | 6.157227 | 4.54E-07 | 1.93E-05 |
| Itga11 | 2.268063 | 5.881055 | 4.59E-07 | 1.94E-05 |
| Col8a2 | 2.25838 | 3.5918 | 4.60E-07 | 1.94E-05 |
| Clca2 | 2.119163 | 5.412342 | 4.68E-07 | 1.97E-05 |
| Thrsp | 2.974071 | 3.127898 | 4.80E-07 | 2.01E-05 |
| Them5 | 3.567062 | 5.072631 | 5.03E-07 | 2.08E-05 |
| Tgm3 | 2.441023 | 6.171229 | 5.10E-07 | 2.11E-05 |
| Aox4 | 3.349113 | 3.386797 | 5.17E-07 | 2.13E-05 |
| Slc15a2 | 4.444841 | 2.040492 | 5.23E-07 | 2.15E-05 |
| Axin2 | 1.916972 | 5.197986 | 5.31E-07 | 2.17E-05 |
| Pfkfb1 | 2.613041 | 4.136199 | 5.43E-07 | 2.21E-05 |
| Timp3 | 1.939166 | 7.475247 | 6.05E-07 | 2.44E-05 |
| Adtrp | 2.79023 | 3.228763 | 6.10E-07 | 2.45E-05 |
| Islr | 2.818572 | 4.413065 | 6.20E-07 | 2.48E-05 |
| Fabp4 | 2.057927 | 6.495684 | 6.75E-07 | 2.65E-05 |
| Fzd10 | 3.922103 | 4.511554 | 7.48E-07 | 2.90E-05 |
| Prlr | 3.927808 | 1.897468 | 8.97E-07 | 3.41E-05 |
| Paqr5 | 2.233987 | 3.844237 | 9.34E-07 | 3.52E-05 |
| Ckmt1 | 2.511132 | 3.879787 | 9.66E-07 | 3.64E-05 |
| Slc38a3 | 2.522374 | 4.387821 | 1.14E-06 | 4.22E-05 |
| Fxyd3 | 2.794338 | 3.53022 | 1.18E-06 | 4.34E-05 |
| Lratd1 | 3.477188 | 5.742571 | 1.22E-06 | 4.45E-05 |
| Emp2 | 2.537915 | 6.985649 | 1.28E-06 | 4.67E-05 |
| Lao1 | 4.259208 | 1.567067 | 1.41E-06 | 5.08E-05 |
| Sptbn2 | 2.323384 | 6.757319 | 1.59E-06 | 5.59E-05 |
| Robo1 | 2.621462 | 5.869229 | 1.65E-06 | 5.77E-05 |
| Xkrx | 2.64153 | 3.464821 | 1.70E-06 | 5.91E-05 |
| Ldhb | 1.933031 | 4.459712 | 1.70E-06 | 5.91E-05 |
| LOC688778 | 2.886927 | 3.402569 | 1.72E-06 | 5.97E-05 |
| Serpinb7 | 3.789844 | 3.820575 | 1.78E-06 | 6.08E-05 |
| Wls | 1.974137 | 7.034836 | 1.79E-06 | 6.10E-05 |
| Tmem54 | 3.849015 | 2.223098 | 2.01E-06 | 6.81E-05 |
| Oxct1 | 1.684956 | 6.963367 | 2.09E-06 | 7.04E-05 |
| Lancl1 | 1.519007 | 5.981611 | 2.15E-06 | 7.24E-05 |
| Col4a5 | 1.85028 | 5.749414 | 2.36E-06 | 7.77E-05 |
| Tnik | 1.616959 | 6.123949 | 2.42E-06 | 7.92E-05 |
| Rps6ka6 | 3.413345 | 2.731454 | 2.45E-06 | 8.00E-05 |
| Aqp4 | 2.721538 | 7.216581 | 2.46E-06 | 8.01E-05 |
| Adipoq | 2.607194 | 4.367474 | 2.53E-06 | 8.21E-05 |
| Fmo2 | 2.736185 | 2.809621 | 2.59E-06 | 8.35E-05 |
| Scel | 1.85204 | 6.623172 | 2.60E-06 | 8.38E-05 |
| Mfsd2a | 3.215508 | 4.366439 | 2.64E-06 | 8.43E-05 |
| Lypd6b | 2.807245 | 3.184229 | 2.64E-06 | 8.43E-05 |
| Rbp7 | 2.742467 | 4.791602 | 2.65E-06 | 8.43E-05 |
| Col6a6 | 2.229491 | 5.814268 | 2.64E-06 | 8.43E-05 |
| Dhcr7 | 1.8302 | 4.80718 | 2.65E-06 | 8.43E-05 |
| Plin1 | 2.820754 | 5.898535 | 2.74E-06 | 8.67E-05 |
| Fap | 1.798009 | 5.056155 | 2.76E-06 | 8.72E-05 |
| Psapl1 | 2.154916 | 4.902732 | 2.93E-06 | 9.18E-05 |
| Sostdc1 | 2.448999 | 2.984517 | 3.09E-06 | 9.64E-05 |
| C7 | 1.978824 | 6.057453 | 3.12E-06 | 9.71E-05 |
| Celsr1 | 2.77923 | 6.425001 | 3.14E-06 | 9.73E-05 |
| Dbi | 1.982281 | 6.404784 | 3.17E-06 | 9.81E-05 |
| Efhd1 | 2.871328 | 4.784613 | 3.20E-06 | 9.89E-05 |
| Col4a3 | 2.494373 | 4.128038 | 3.21E-06 | 9.89E-05 |
| Cd9 | 1.962925 | 6.058782 | 3.36E-06 | 0.000103 |
| Acvr1c | 4.19466 | 1.074652 | 3.42E-06 | 0.000105 |
| Lgr4 | 1.79238 | 5.730708 | 3.44E-06 | 0.000105 |
| Bmp7 | 2.968627 | 4.359744 | 3.50E-06 | 0.000107 |
| Mdh1 | 1.72368 | 6.795574 | 3.54E-06 | 0.000108 |
| Cttnbp2 | 1.750054 | 5.098182 | 3.62E-06 | 0.00011 |
| Cst3 | 1.600269 | 6.524618 | 3.65E-06 | 0.00011 |
| Adrb3 | 3.258333 | 1.590373 | 3.68E-06 | 0.000111 |
| Slc6a19 | 3.129089 | 2.413064 | 3.71E-06 | 0.000111 |
| Ephb6 | 2.003821 | 5.753446 | 3.71E-06 | 0.000111 |
| Cbln1 | 2.761948 | 3.265012 | 3.77E-06 | 0.000112 |
| Rps9 | 4.184686 | 4.248382 | 3.82E-06 | 0.000113 |
| Krt2 | 3.835029 | 2.541618 | 3.82E-06 | 0.000113 |
| Trpv6 | 2.214917 | 3.976027 | 3.92E-06 | 0.000116 |
| Sbk2 | 3.443002 | 2.412494 | 4.01E-06 | 0.000118 |
| Elovl4 | 2.824565 | 6.711727 | 4.07E-06 | 0.00012 |
| Abca8a | 2.325817 | 7.644254 | 4.11E-06 | 0.00012 |
| Gjb5 | 3.012252 | 2.199433 | 4.17E-06 | 0.000121 |
| Efna3 | 3.006196 | 2.974515 | 4.18E-06 | 0.000122 |
| LOC103689947 | 6.861006 | 4.062473 | 4.27E-06 | 0.000124 |
| Lhx2 | 3.271667 | 3.787329 | 4.28E-06 | 0.000124 |
| AABR07031526.1 | 2.986149 | 2.580338 | 4.47E-06 | 0.000128 |
| Bnipl | 2.322577 | 3.976022 | 4.48E-06 | 0.000129 |
| Tenm2 | 3.16594 | 5.812887 | 4.88E-06 | 0.000139 |
| Cers4 | 1.662775 | 5.014174 | 4.89E-06 | 0.000139 |
| Hhip | 5.336505 | 2.14499 | 4.91E-06 | 0.000139 |
| AABR07044375.1 | 1.569569 | 5.025569 | 4.95E-06 | 0.00014 |
| Slc6a4 | 2.304751 | 4.048873 | 4.98E-06 | 0.00014 |
| Dpep1 | 2.082991 | 3.658844 | 5.20E-06 | 0.000146 |
| Grhl2 | 2.480453 | 4.791832 | 5.32E-06 | 0.000148 |
| Galnt5 | 2.018316 | 3.742184 | 5.36E-06 | 0.000149 |
| Dsc1 | 2.777 | 7.202589 | 5.41E-06 | 0.00015 |
| Ankef1 | 2.608059 | 3.749725 | 5.49E-06 | 0.000152 |
| Pmel | 5.086071 | 5.123382 | 5.84E-06 | 0.000161 |
| Vdr | 2.570308 | 5.6898 | 5.85E-06 | 0.000161 |
| Clec3b | 1.900856 | 6.13033 | 5.96E-06 | 0.000164 |
| Lamc3 | 2.64785 | 3.202689 | 6.37E-06 | 0.000173 |
| Sema7a | 1.678504 | 4.988748 | 6.49E-06 | 0.000176 |
| Esrp1 | 2.281961 | 6.953051 | 6.54E-06 | 0.000177 |
| Endou | 2.120274 | 5.186568 | 6.63E-06 | 0.000179 |
| Ly6d | 2.444684 | 5.400603 | 6.79E-06 | 0.000182 |
| Sptlc3 | 2.647852 | 3.883832 | 6.88E-06 | 0.000184 |
| Cdh1 | 2.290208 | 7.393757 | 6.90E-06 | 0.000185 |
| Vangl2 | 3.633577 | 5.152148 | 6.99E-06 | 0.000187 |
| Fndc1 | 2.393472 | 8.111965 | 7.16E-06 | 0.000191 |
| Moxd1 | 2.488542 | 3.975632 | 7.50E-06 | 0.000198 |
| Mboat2 | 2.152307 | 3.09644 | 8.68E-06 | 0.000226 |
| Fgfr3 | 2.177366 | 5.302767 | 9.47E-06 | 0.000243 |
| Rapgefl1 | 2.070133 | 4.385604 | 9.47E-06 | 0.000243 |
| Echdc1 | 1.625282 | 5.303656 | 9.49E-06 | 0.000243 |
| Akr1c2 | 3.758197 | 2.651696 | 9.54E-06 | 0.000243 |
| Celsr2 | 2.872841 | 7.29314 | 9.52E-06 | 0.000243 |
| Ivd | 1.632552 | 6.280737 | 9.54E-06 | 0.000243 |
| Sytl1 | 1.984681 | 4.17474 | 9.70E-06 | 0.000245 |
| Dact2 | 2.288426 | 3.007696 | 9.86E-06 | 0.000248 |
| Msx1 | 1.851401 | 3.945917 | 9.85E-06 | 0.000248 |
| Gjb3 | 2.61703 | 2.499564 | 9.90E-06 | 0.000248 |
| Calhm5 | 3.691834 | 2.793554 | 9.97E-06 | 0.000249 |
| Pnpla3 | 2.52524 | 3.150384 | 9.99E-06 | 0.000249 |
| Irf6 | 2.343985 | 6.436091 | 1.00E-05 | 0.00025 |
| Sdr16c6 | 2.916427 | 4.357688 | 1.04E-05 | 0.000258 |
| Cldn8 | 6.093967 | 2.150193 | 1.05E-05 | 0.000259 |
| Hadh | 1.586785 | 4.709256 | 1.05E-05 | 0.00026 |
| Adamtsl2 | 2.471026 | 2.972984 | 1.06E-05 | 0.00026 |
| Serpinb13 | 3.459848 | 2.943152 | 1.10E-05 | 0.000269 |
| Rab25 | 2.383046 | 4.641562 | 1.11E-05 | 0.000271 |
| Neu3 | 2.482932 | 2.656419 | 1.13E-05 | 0.000275 |
| AABR07072112.1 | 4.167599 | 1.053899 | 1.16E-05 | 0.000281 |
| Wfdc21 | 4.83337 | 4.070864 | 1.17E-05 | 0.000282 |
| Trim29 | 2.581203 | 7.860082 | 1.19E-05 | 0.000285 |
| Tril | 1.64664 | 5.057324 | 1.23E-05 | 0.000295 |
| Scd | 2.13244 | 7.572091 | 1.25E-05 | 0.000299 |
| Gjb4 | 2.612857 | 3.443725 | 1.31E-05 | 0.000309 |
| Ppl | 1.691599 | 7.970382 | 1.33E-05 | 0.000312 |
| Tmem179 | 3.440251 | 1.266501 | 1.37E-05 | 0.00032 |
| Mpped2 | 2.554466 | 2.393351 | 1.37E-05 | 0.00032 |
| Smtnl1 | 2.241239 | 6.736053 | 1.40E-05 | 0.000328 |
| Znf750 | 2.671442 | 5.656474 | 1.45E-05 | 0.000337 |
| Fabp3 | 2.22291 | 4.913588 | 1.51E-05 | 0.00035 |
| Cst6 | 1.597726 | 4.654747 | 1.58E-05 | 0.000366 |
| Gpr87 | 2.787881 | 3.883403 | 1.62E-05 | 0.000373 |
| Acsbg1 | 2.116759 | 6.20618 | 1.63E-05 | 0.000375 |
| Cacna1g | 1.807426 | 4.553106 | 1.63E-05 | 0.000375 |
| Dlx3 | 5.314563 | 6.643691 | 1.64E-05 | 0.000375 |
| Tmem45a | 2.293588 | 2.916409 | 1.63E-05 | 0.000375 |
| Rgs6 | 2.626822 | 3.328535 | 1.64E-05 | 0.000375 |
| Perm1 | 2.217431 | 6.114714 | 1.67E-05 | 0.000379 |
| Ca13 | 2.019723 | 4.157602 | 1.72E-05 | 0.000391 |
| Mb | 1.823823 | 8.167233 | 1.78E-05 | 0.000405 |
| LOC681458 | 3.825388 | 6.861994 | 1.80E-05 | 0.000407 |
| Enpp1 | 1.802573 | 4.114448 | 1.82E-05 | 0.000412 |
| Mycn | 4.52626 | 3.902014 | 1.85E-05 | 0.000415 |
| Trpm1 | 3.647742 | 4.199189 | 1.86E-05 | 0.000418 |
| Ces4a | 4.028152 | 0.909494 | 1.88E-05 | 0.00042 |
| Spint1 | 3.09445 | 5.01862 | 1.88E-05 | 0.00042 |
| LOC688553 | 3.05659 | 2.750466 | 1.94E-05 | 0.00043 |
| Arhgef19 | 2.873853 | 3.403058 | 1.97E-05 | 0.000436 |
| Bicdl2 | 3.371428 | 4.674467 | 2.00E-05 | 0.000439 |
| Fitm2 | 1.538983 | 5.437689 | 2.03E-05 | 0.000446 |
| Cntn1 | 2.184489 | 2.896447 | 2.05E-05 | 0.000449 |
| Sema5a | 1.672249 | 6.348732 | 2.05E-05 | 0.000449 |
| AC139642.1 | 1.845183 | 6.372644 | 2.07E-05 | 0.000452 |
| Pnpla5 | 3.310622 | 2.412547 | 2.12E-05 | 0.000462 |
| Cited4 | 2.755853 | 3.544799 | 2.13E-05 | 0.000463 |
| Krt77 | 4.417905 | 8.422666 | 2.16E-05 | 0.000468 |
| Ocln | 2.248383 | 3.627098 | 2.19E-05 | 0.000473 |
| Lrig3 | 2.018988 | 5.338186 | 2.19E-05 | 0.000473 |
| Prss12 | 2.000238 | 3.49887 | 2.25E-05 | 0.000485 |
| Fads6 | 2.371382 | 2.441623 | 2.28E-05 | 0.000489 |
| Edaradd | 2.842769 | 3.314286 | 2.29E-05 | 0.000489 |
| Kit | 1.624826 | 5.492204 | 2.38E-05 | 0.000507 |
| LOC686143 | 2.905587 | 4.625063 | 2.41E-05 | 0.00051 |
| Ccdc152 | 1.724719 | 4.456901 | 2.44E-05 | 0.000516 |
| Cdc42bpg | 1.85011 | 6.518884 | 2.44E-05 | 0.000516 |
| Dlx2 | 5.608073 | 2.260314 | 2.47E-05 | 0.000521 |
| Awat1 | 3.515603 | 2.726026 | 2.50E-05 | 0.000525 |
| Cilp | 2.314278 | 6.99411 | 2.51E-05 | 0.000526 |
| Lep | 2.64307 | 2.095502 | 2.61E-05 | 0.000544 |
| Slc7a10 | 2.367257 | 2.997043 | 2.61E-05 | 0.000544 |
| Kazald1 | 2.745266 | 2.403208 | 2.68E-05 | 0.000558 |
| Col4a4 | 1.933521 | 6.660371 | 2.69E-05 | 0.000558 |
| Epcam | 2.401517 | 4.669809 | 2.70E-05 | 0.000559 |
| Robo2 | 1.688304 | 4.264583 | 2.88E-05 | 0.000592 |
| Cpt2 | 1.372045 | 5.35898 | 2.90E-05 | 0.000595 |
| Hbb | 1.642518 | 6.177891 | 2.95E-05 | 0.000601 |
| Padi2 | 1.891691 | 5.356233 | 3.05E-05 | 0.000622 |
| Lrp4 | 1.553836 | 6.599829 | 3.07E-05 | 0.000624 |
| Lhfpl1 | 3.584595 | 2.780666 | 3.08E-05 | 0.000625 |
| Golga7b | 4.933489 | 4.096941 | 3.12E-05 | 0.000631 |
| Esrrg | 2.535004 | 3.402619 | 3.11E-05 | 0.000631 |
| Tfap2c | 2.363854 | 4.761234 | 3.16E-05 | 0.000636 |
| Agt | 2.113718 | 2.829214 | 3.17E-05 | 0.000638 |
| Il1f10 | 3.970371 | 2.592799 | 3.26E-05 | 0.000654 |
| Foxq1 | 3.085468 | 2.855241 | 3.33E-05 | 0.000667 |
| Ckmt2 | 2.25373 | 6.73725 | 3.37E-05 | 0.000671 |
| Prom2 | 1.859106 | 6.234064 | 3.37E-05 | 0.000672 |
| Lgr6 | 2.382706 | 2.627122 | 3.41E-05 | 0.000676 |
| Cox7b | 1.4561 | 5.97069 | 3.53E-05 | 0.000698 |
| Fat2 | 2.14079 | 6.926456 | 3.55E-05 | 0.000699 |
| Terc | 1.807217 | 3.65846 | 3.65E-05 | 0.000718 |
| Cyp2b21 | 3.312658 | 2.206735 | 3.65E-05 | 0.000718 |
| Fads2l1 | 2.23981 | 3.885175 | 3.66E-05 | 0.000719 |
| Bmp4 | 1.891076 | 4.177876 | 3.74E-05 | 0.000734 |
| Loxl2 | 1.608789 | 6.301122 | 3.80E-05 | 0.000742 |
| Sec14l2 | 1.843312 | 4.433163 | 3.81E-05 | 0.000743 |
| Rassf6 | 4.003341 | 2.099763 | 3.84E-05 | 0.000748 |
| Map2k6 | 1.861084 | 4.359816 | 3.91E-05 | 0.000759 |
| RGD1559622 | 3.470218 | 4.637899 | 3.95E-05 | 0.000766 |
| Plbd1 | 1.486409 | 5.223097 | 3.98E-05 | 0.00077 |
| Wnt10b | 4.39684 | 1.794494 | 4.00E-05 | 0.000772 |
| Lgr5 | 4.328279 | 3.879047 | 4.06E-05 | 0.000782 |
| Serpini1 | 2.838629 | 1.818291 | 4.09E-05 | 0.000785 |
| Phgdh | 1.903564 | 4.048491 | 4.14E-05 | 0.000793 |
| Hs3st6 | 2.827449 | 1.55153 | 4.23E-05 | 0.000805 |
| Barx2 | 1.717519 | 4.684527 | 4.38E-05 | 0.000829 |
| AABR07030143.1 | 4.561619 | 1.656311 | 4.60E-05 | 0.00086 |
| Adh6 | 2.950469 | 1.99835 | 4.66E-05 | 0.000869 |
| Serpinb12 | 3.275714 | 4.5815 | 4.67E-05 | 0.000871 |
| Wfdc1 | 2.187756 | 3.184269 | 4.67E-05 | 0.000871 |
| LOC102548472 | 3.438958 | 0.932555 | 4.72E-05 | 0.000876 |
| Hes1 | 2.047687 | 4.522425 | 4.79E-05 | 0.000888 |
| Dsp | 2.485521 | 10.63704 | 4.83E-05 | 0.000892 |
| Deptor | 1.400394 | 5.273107 | 5.09E-05 | 0.00094 |
| Sema3e | 2.573869 | 4.573003 | 5.14E-05 | 0.000948 |
| Abcd2 | 2.236826 | 4.191767 | 5.17E-05 | 0.000952 |
| AABR07028446.1 | 2.607955 | 6.281556 | 5.26E-05 | 0.000967 |
| Lce1m | 3.702711 | 4.016698 | 5.36E-05 | 0.000982 |
| Msx2 | 5.01691 | 5.301732 | 5.39E-05 | 0.000984 |
| Dsc3 | 2.370162 | 9.280865 | 5.40E-05 | 0.000985 |
| Ly6g6c | 1.79244 | 3.876672 | 5.42E-05 | 0.000988 |
| Clstn2 | 3.621496 | 1.035935 | 5.45E-05 | 0.00099 |
| Slc34a2 | 2.107891 | 2.919865 | 5.48E-05 | 0.000994 |
| Adgra3 | 2.031831 | 6.123358 | 5.51E-05 | 0.000999 |
| Foxred2 | 1.789053 | 3.948046 | 5.72E-05 | 0.001034 |
| Pdzd2 | 1.904823 | 6.797313 | 5.78E-05 | 0.001041 |
| Mfsd6 | 1.529466 | 5.669519 | 5.83E-05 | 0.001048 |
| Mamdc2 | 2.701649 | 3.03783 | 5.90E-05 | 0.001056 |
| Cyp2b12 | 3.448856 | 3.571018 | 5.98E-05 | 0.001067 |
| Il20ra | 3.384781 | 1.994714 | 6.14E-05 | 0.001093 |
| Gstt3 | 2.345544 | 2.627459 | 6.17E-05 | 0.001097 |
| Esrp2 | 2.022091 | 5.999499 | 6.35E-05 | 0.001123 |
| Zfp185 | 2.412248 | 4.834205 | 6.38E-05 | 0.001127 |
| Pla2g2f | 2.220138 | 4.570549 | 6.39E-05 | 0.001127 |
| Kif26a | 2.130438 | 5.16821 | 6.47E-05 | 0.00114 |
| Dsg2 | 3.029561 | 5.167777 | 6.52E-05 | 0.001148 |
| AABR07072759.1 | 7.650688 | 3.604442 | 6.63E-05 | 0.001164 |
| Sp6 | 4.28866 | 5.081854 | 6.66E-05 | 0.001166 |
| Slc39a10 | 3.704177 | 8.266635 | 6.66E-05 | 0.001166 |
| Upk1b | 4.595461 | 3.025157 | 6.86E-05 | 0.001194 |
| Tmprss7 | 4.010655 | 0.896184 | 7.19E-05 | 0.001245 |
| Arl13b | 1.671278 | 4.892855 | 7.19E-05 | 0.001245 |
| LOC103694397 | 7.256245 | 3.226969 | 7.22E-05 | 0.001248 |
| Trim2 | 2.201765 | 4.312777 | 7.29E-05 | 0.001255 |
| Creb5 | 2.012552 | 5.067137 | 7.35E-05 | 0.001263 |
| Prss8 | 1.950233 | 3.521718 | 7.45E-05 | 0.00128 |
| Myl2 | 3.547817 | 3.809849 | 7.54E-05 | 0.001293 |
| Ntsr1 | 2.93199 | 2.485304 | 7.82E-05 | 0.001334 |
| Arhgef5 | 1.445931 | 6.143401 | 7.84E-05 | 0.001336 |
| Ppp2r2b | 2.850918 | 1.789269 | 8.25E-05 | 0.001402 |
| Nectin4 | 2.189267 | 5.992535 | 8.29E-05 | 0.001407 |
| Krt72 | 6.63749 | 4.394422 | 8.46E-05 | 0.001433 |
| Hoxc13 | 5.481959 | 3.957376 | 8.47E-05 | 0.001433 |
| Efnb2 | 2.091995 | 6.415268 | 8.63E-05 | 0.001457 |
| Myh7 | 3.414332 | 7.922943 | 8.75E-05 | 0.001475 |
| Pxmp4 | 1.615096 | 4.495473 | 9.37E-05 | 0.001572 |
| Heph | 1.421694 | 4.756372 | 9.51E-05 | 0.001586 |
| Sult1a1 | 1.807861 | 4.098941 | 9.71E-05 | 0.001611 |
| Fzd7 | 1.412328 | 6.160919 | 9.86E-05 | 0.001635 |
| Ano9 | 2.699026 | 4.397846 | 0.000102 | 0.001677 |
| AABR07044583.2 | 7.726715 | 3.357244 | 0.000103 | 0.001692 |
| Apobec2 | 2.151727 | 9.021913 | 0.000103 | 0.001699 |
| Cidea | 2.616399 | 1.948945 | 0.000104 | 0.00171 |
| Cd34 | 1.588787 | 6.614984 | 0.000107 | 0.001737 |
| Gabrp | 4.992603 | 2.795311 | 0.000107 | 0.001743 |
| Padi1 | 4.940218 | 2.525997 | 0.000108 | 0.001745 |
| Cd207 | 3.073512 | 2.904587 | 0.000109 | 0.001759 |
| Acsm2 | 2.882349 | 2.270704 | 0.00011 | 0.001773 |
| Wnt11 | 3.360441 | 3.264493 | 0.000112 | 0.001806 |
| Lef1 | 4.445305 | 5.364868 | 0.000113 | 0.001815 |
| Aqp7 | 3.070618 | 1.503514 | 0.000114 | 0.001826 |
| Tdo2 | 3.690133 | 0.705212 | 0.000114 | 0.001831 |
| NEWGENE_620180 | 2.194951 | 2.846793 | 0.000115 | 0.001847 |
| Adhfe1 | 2.524838 | 3.482736 | 0.000116 | 0.001847 |
| Tfap2e | 2.408443 | 1.826146 | 0.000118 | 0.001875 |
| RGD1560324 | 2.908127 | 2.613197 | 0.000119 | 0.001882 |
| Wnt5a | 2.594252 | 5.356631 | 0.000119 | 0.001882 |
| Zfp93 | 3.078397 | 1.387959 | 0.000119 | 0.001892 |
| Irx4 | 3.767462 | 2.340524 | 0.00012 | 0.001893 |
| Flrt1 | 1.601461 | 4.136732 | 0.000121 | 0.001913 |
| Krt36 | 5.827267 | 3.076364 | 0.000123 | 0.00193 |
| RGD1309350 | 2.658062 | 2.119614 | 0.000125 | 0.001962 |
| Cadm1 | 1.757248 | 3.736576 | 0.000126 | 0.001971 |
| Rdh12 | 2.1995 | 2.860945 | 0.000128 | 0.001994 |
| LOC690478 | 6.579504 | 2.59182 | 0.000128 | 0.002001 |
| Lce1l | 2.904422 | 4.192671 | 0.000132 | 0.002053 |
| Csta | 2.154788 | 2.548969 | 0.000134 | 0.002079 |
| Adamts19 | 2.73731 | 1.877573 | 0.000137 | 0.002115 |
| RGD1563601 | 1.408393 | 5.746208 | 0.000139 | 0.002151 |
| Btbd3 | 1.572419 | 5.039903 | 0.000142 | 0.002175 |
| S100a3 | 6.377311 | 6.897842 | 0.000143 | 0.002185 |
| Crct1 | 1.536509 | 4.497319 | 0.000143 | 0.002187 |
| Fzd6 | 1.776958 | 5.836734 | 0.000148 | 0.002252 |
| Elovl7 | 2.408211 | 2.822368 | 0.000149 | 0.00226 |
| Acan | 5.696149 | 1.509902 | 0.000149 | 0.002267 |
| Myoz2 | 2.021 | 4.913294 | 0.000151 | 0.002287 |
| Selenop | 1.644874 | 8.241933 | 0.000152 | 0.002299 |
| Ptpn3 | 1.806231 | 7.106382 | 0.000155 | 0.002339 |
| Pecr | 1.703996 | 3.358424 | 0.000157 | 0.002372 |
| Ddit4l2 | 2.201016 | 5.954702 | 0.000158 | 0.002376 |
| Acsl6 | 2.138741 | 4.822173 | 0.00016 | 0.002399 |
| Evpl | 1.736629 | 6.737015 | 0.00016 | 0.0024 |
| Alox12 | 1.725978 | 3.694109 | 0.00016 | 0.0024 |
| Rab11fip4 | 2.527009 | 4.40672 | 0.000161 | 0.002414 |
| Kcnab3 | 2.768951 | 1.221724 | 0.000165 | 0.002466 |
| Fbp1 | 6.26107 | 2.300266 | 0.000166 | 0.002471 |
| Ephb3 | 2.121386 | 4.90769 | 0.000166 | 0.002471 |
| LOC100361702 | 6.566841 | 3.278909 | 0.000169 | 0.002508 |
| Slc45a2 | 5.52156 | 1.364414 | 0.00017 | 0.002525 |
| Zic1 | 2.453901 | 3.176474 | 0.00017 | 0.002528 |
| Cib2 | 1.697516 | 3.688188 | 0.000173 | 0.002551 |
| Entpd3 | 5.984731 | 2.732956 | 0.000176 | 0.002589 |
| Krt28 | 5.897827 | 4.974336 | 0.000178 | 0.002624 |
| Tceal7 | 1.978864 | 5.498253 | 0.000179 | 0.00263 |
| Serpina12 | 3.258686 | 3.238847 | 0.00018 | 0.002636 |
| Slitrk6 | 2.661017 | 1.665364 | 0.000186 | 0.002718 |
| Serpina1 | 4.769586 | 3.01345 | 0.000188 | 0.002741 |
| Lpar3 | 3.047304 | 2.293668 | 0.000189 | 0.002754 |
| Lrrc15 | 5.395388 | 4.511041 | 0.00019 | 0.00276 |
| Myl9 | 1.404209 | 5.272735 | 0.000192 | 0.002784 |
| Prr9 | 5.283091 | 4.34468 | 0.000195 | 0.002821 |
| Gpt | 1.558798 | 4.92285 | 0.000195 | 0.002822 |
| Igfbp6 | 1.786021 | 5.543144 | 0.000196 | 0.002826 |
| Aldh1a3 | 4.431356 | 5.051288 | 0.000199 | 0.00287 |
| AABR07034648.1 | 2.21377 | 2.674223 | 0.000203 | 0.002923 |
| Plek2 | 1.808457 | 3.932318 | 0.000206 | 0.002954 |
| Hsd17b14 | 3.852697 | 4.418605 | 0.000207 | 0.002964 |
| RGD1561557 | 5.96782 | 3.480503 | 0.000209 | 0.002979 |
| Pank1 | 1.510196 | 4.050988 | 0.000209 | 0.002979 |
| Kprp | 2.890535 | 5.855235 | 0.000217 | 0.003081 |
| Srcin1 | 2.071605 | 4.96022 | 0.000219 | 0.003104 |
| Tns4 | 1.6242 | 4.651889 | 0.00022 | 0.003109 |
| Sdr16c5 | 2.580922 | 2.018826 | 0.000222 | 0.003136 |
| Tfap2a | 2.032 | 5.217462 | 0.000223 | 0.003156 |
| Bche | 1.975555 | 2.988819 | 0.000224 | 0.003167 |
| Cdh4 | 2.995417 | 0.941474 | 0.000226 | 0.003178 |
| Il22ra1 | 1.838446 | 3.308342 | 0.000225 | 0.003178 |
| Rmdn1 | 1.479063 | 5.010033 | 0.000233 | 0.003267 |
| Itm2b | 1.379674 | 8.318784 | 0.000242 | 0.003389 |
| Cntfr | 3.297129 | 4.168314 | 0.000244 | 0.003403 |
| Igsf9 | 2.068165 | 4.508318 | 0.000243 | 0.003403 |
| Qpct | 2.043465 | 2.294965 | 0.000245 | 0.003418 |
| LOC100361739 | 6.081155 | 3.588358 | 0.000245 | 0.003418 |
| Ptpn13 | 1.630529 | 7.069162 | 0.000249 | 0.003461 |
| RGD1561161 | 1.591089 | 6.121116 | 0.000252 | 0.003487 |
| Pcsk6 | 1.432267 | 5.493444 | 0.000253 | 0.003502 |
| Prr32 | 2.43271 | 3.419395 | 0.000256 | 0.003537 |
| AABR07044302.1 | 2.517234 | 2.729098 | 0.000265 | 0.003642 |
| Epb41l4b | 1.650943 | 4.70038 | 0.000268 | 0.003675 |
| Foxn1 | 3.539466 | 4.433814 | 0.00027 | 0.003698 |
| AABR07038886.1 | 3.851977 | 1.241556 | 0.000274 | 0.003749 |
| Btc | 2.659096 | 1.779144 | 0.000275 | 0.003756 |
| Lmod1 | 2.094371 | 6.668973 | 0.000275 | 0.003756 |
| Gli2 | 1.949069 | 4.462367 | 0.000279 | 0.003805 |
| Rhou | 2.015751 | 2.523625 | 0.000281 | 0.003825 |
| Sod3 | 1.543472 | 6.307277 | 0.000281 | 0.003831 |
| LOC686720 | 6.772524 | 2.770823 | 0.000285 | 0.003873 |
| Far2 | 2.330899 | 3.224936 | 0.000288 | 0.003895 |
| Arhgef26 | 1.580168 | 3.977633 | 0.00029 | 0.003911 |
| Nkd1 | 1.73383 | 3.398422 | 0.000302 | 0.004069 |
| Fut1 | 2.429812 | 3.800461 | 0.000303 | 0.004077 |
| Mycl | 2.150049 | 4.90202 | 0.000309 | 0.004144 |
| Mbnl3 | 2.64615 | 2.422772 | 0.00031 | 0.004145 |
| LOC102547817 | 3.027266 | 0.963985 | 0.00031 | 0.004146 |
| S100a16 | 1.420265 | 5.575446 | 0.000327 | 0.004344 |
| Dsg4 | 5.847631 | 6.353693 | 0.00033 | 0.004374 |
| Rerg | 1.963624 | 3.709568 | 0.00033 | 0.004374 |
| Ptpru | 2.29463 | 1.887206 | 0.000345 | 0.004558 |
| Slc25a27 | 1.667332 | 3.168772 | 0.000345 | 0.004561 |
| RT1-Ba | 1.551646 | 5.332901 | 0.000346 | 0.004566 |
| Smoc1 | 1.818065 | 2.795204 | 0.000353 | 0.004642 |
| Mal2 | 2.080916 | 3.244019 | 0.000356 | 0.004672 |
| LOC687631 | 6.681777 | 3.254798 | 0.000359 | 0.004692 |
| Capn8 | 3.475627 | 2.315438 | 0.00036 | 0.004701 |
| Krt40 | 6.816032 | 3.927156 | 0.000362 | 0.004724 |
| Mgst1 | 1.523347 | 4.830388 | 0.000363 | 0.004729 |
| AABR07060560.3 | 2.046226 | 2.515317 | 0.000366 | 0.004757 |
| Cbs | 3.560508 | 4.316335 | 0.000368 | 0.004761 |
| Ech1 | 1.404284 | 5.832355 | 0.000372 | 0.004802 |
| Krt71 | 6.519546 | 9.908932 | 0.000381 | 0.004907 |
| Slc27a3 | 1.415533 | 4.744313 | 0.000381 | 0.004907 |
| Tnni1 | 2.295063 | 5.974586 | 0.000384 | 0.004922 |
| Krtdap | 2.198608 | 5.417863 | 0.000383 | 0.004922 |
| Ntrk2 | 1.464596 | 6.61044 | 0.000383 | 0.004922 |
| Cacna1e | 2.081307 | 2.194233 | 0.000387 | 0.00495 |
| Myh3 | 1.730258 | 5.348592 | 0.000388 | 0.00495 |
| LOC100365958 | 1.859254 | 2.767121 | 0.000397 | 0.005034 |
| Pdgfrl | 2.091075 | 2.992971 | 0.000399 | 0.005049 |
| Wif1 | 2.868796 | 2.255208 | 0.000399 | 0.005054 |
| Ankrd22 | 1.81203 | 2.861505 | 0.000402 | 0.005084 |
| Cytl1 | 2.067137 | 4.145949 | 0.00042 | 0.005276 |
| Alox12e | 2.420913 | 1.637648 | 0.000426 | 0.005345 |
| Psors1c2 | 5.928292 | 5.333443 | 0.000428 | 0.00536 |
| AABR07044583.1 | 6.21404 | 3.317232 | 0.000436 | 0.005407 |
| Il36rn | 1.718201 | 3.512872 | 0.000438 | 0.005424 |
| Shroom3 | 1.958541 | 5.157312 | 0.000439 | 0.00543 |
| Hmgcs2 | 2.560352 | 3.037346 | 0.00044 | 0.005442 |
| Mlph | 2.506085 | 1.972289 | 0.000442 | 0.005454 |
| Mpp7 | 2.093758 | 3.864542 | 0.000442 | 0.005455 |
| Dpyd | 2.186815 | 2.520646 | 0.000447 | 0.005506 |
| Cfap57 | 2.656914 | 1.91735 | 0.000448 | 0.005519 |
| Krtap22-2 | 6.58879 | 3.708377 | 0.000449 | 0.005527 |
| LOC690460 | 5.299073 | 1.940202 | 0.00045 | 0.005532 |
| Ly6g6g | 6.391169 | 2.114407 | 0.000451 | 0.005543 |
| Ccdc80 | 1.467397 | 8.214913 | 0.000453 | 0.005551 |
| Etv4 | 2.33679 | 4.822046 | 0.000454 | 0.005556 |
| Stard10 | 1.481595 | 4.571929 | 0.000454 | 0.005556 |
| Fabp5 | 2.26074 | 7.480481 | 0.000457 | 0.005579 |
| Rab15 | 2.1886 | 4.459073 | 0.000468 | 0.005698 |
| Lsr | 1.712631 | 5.34099 | 0.000471 | 0.005721 |
| Gstm7 | 1.523989 | 5.91942 | 0.000479 | 0.005813 |
| Krtap24-1 | 6.248061 | 3.473832 | 0.00048 | 0.005814 |
| Mroh6 | 1.517912 | 4.418113 | 0.000485 | 0.005863 |
| Npr3 | 1.750195 | 3.025726 | 0.000489 | 0.005888 |
| AABR07045487.1 | 2.401369 | 3.631117 | 0.000497 | 0.005971 |
| Gabra4 | 4.562265 | 2.824621 | 0.000499 | 0.005981 |
| AABR07030502.1 | 6.20939 | 3.437048 | 0.000501 | 0.005994 |
| Cabcoco1 | 1.954691 | 2.547509 | 0.000502 | 0.006005 |
| LOC100361664 | 5.956724 | 4.162998 | 0.000514 | 0.006133 |
| Fam83g | 1.949033 | 4.624648 | 0.000514 | 0.006133 |
| Ndufa12 | 1.93912 | 3.34317 | 0.00052 | 0.006199 |
| Creb3l4 | 2.159232 | 2.432221 | 0.000521 | 0.006205 |
| Gfra3 | 2.818122 | 1.643377 | 0.000526 | 0.006261 |
| Fam81a | 2.27119 | 2.375163 | 0.000529 | 0.006282 |
| Hyal1 | 1.736933 | 4.394454 | 0.000534 | 0.006335 |
| Hist3h2ba | 4.586072 | 1.674692 | 0.000538 | 0.006375 |
| Nrg2 | 2.659258 | 1.999367 | 0.000539 | 0.006377 |
| Kyat3 | 1.893159 | 2.370931 | 0.000546 | 0.006451 |
| Hephl1 | 4.727254 | 5.567138 | 0.000549 | 0.006473 |
| Bmp5 | 2.235798 | 2.702152 | 0.000549 | 0.006473 |
| Unc5b | 2.896905 | 6.467919 | 0.000552 | 0.006503 |
| Rnf180 | 4.522077 | 3.134125 | 0.000567 | 0.006654 |
| Krt85 | 6.651786 | 8.922613 | 0.000569 | 0.006679 |
| Krt25 | 7.061086 | 9.100696 | 0.000572 | 0.006707 |
| Samd5 | 3.43242 | 4.335356 | 0.00058 | 0.006789 |
| Hapln4 | 3.409206 | 0.616567 | 0.000582 | 0.006797 |
| Tmem65 | 1.369802 | 4.328127 | 0.000587 | 0.006841 |
| Nkd2 | 2.06227 | 5.153158 | 0.0006 | 0.006963 |
| RGD1564854 | 2.876174 | 0.644943 | 0.000602 | 0.006978 |
| Rbp4 | 2.249135 | 2.530281 | 0.000605 | 0.006997 |
| Rcan2 | 1.73297 | 4.407754 | 0.000612 | 0.007078 |
| Fhdc1 | 1.742892 | 5.029122 | 0.000613 | 0.007082 |
| Plxnb1 | 1.610438 | 6.158332 | 0.000615 | 0.007089 |
| Egflam | 1.40184 | 4.175062 | 0.000617 | 0.007108 |
| Serpina11 | 4.466599 | 2.106857 | 0.000623 | 0.007156 |
| Cyp2j16 | 3.981878 | 1.025335 | 0.000623 | 0.007156 |
| Arap2 | 1.707861 | 6.307131 | 0.000625 | 0.007167 |
| Ppargc1a | 1.785734 | 4.064734 | 0.000627 | 0.007176 |
| Tub | 2.823539 | 0.822948 | 0.000637 | 0.00726 |
| Ackr2 | 2.052272 | 3.30067 | 0.000637 | 0.007261 |
| Flg2 | 2.875401 | 5.774413 | 0.000643 | 0.007306 |
| Epha1 | 1.824504 | 5.514197 | 0.000643 | 0.007306 |
| Acadm | 1.41905 | 6.498206 | 0.000651 | 0.007387 |
| Sh2d4a | 1.773728 | 3.605514 | 0.000655 | 0.007421 |
| Krt32 | 5.789685 | 6.70735 | 0.000659 | 0.007454 |
| Gli1 | 2.11976 | 4.255257 | 0.000667 | 0.007534 |
| Rgcc | 1.844803 | 2.453636 | 0.000672 | 0.007588 |
| Grhl1 | 1.683406 | 6.267358 | 0.000676 | 0.007614 |
| Pkp3 | 1.771362 | 6.380383 | 0.000681 | 0.007664 |
| Lce6a | 2.213901 | 1.557178 | 0.000682 | 0.007668 |
| Krt1 | 2.977114 | 9.396775 | 0.000684 | 0.007683 |
| Krt35 | 6.098039 | 7.444425 | 0.000686 | 0.00769 |
| Serpinb3a | 2.037538 | 5.166305 | 0.000686 | 0.00769 |
| AABR07060833.1 | 3.425836 | 0.526096 | 0.000694 | 0.007751 |
| LOC102552326 | 2.761741 | 3.572387 | 0.000695 | 0.007753 |
| Itgb1bp2 | 1.703471 | 5.039704 | 0.000695 | 0.007753 |
| Cdh6 | 2.851468 | 3.970889 | 0.000697 | 0.007764 |
| Krt8 | 3.227171 | 2.248715 | 0.000699 | 0.007774 |
| Rassf10 | 2.657615 | 2.107213 | 0.000711 | 0.007902 |
| Kcnma1 | 1.772878 | 4.98545 | 0.000742 | 0.008202 |
| Cpa4 | 1.473437 | 5.045745 | 0.000743 | 0.008202 |
| Bpifc | 1.873653 | 4.469204 | 0.000748 | 0.008259 |
| Notum | 3.53824 | 1.615794 | 0.00075 | 0.008264 |
| Kifc2 | 1.956511 | 2.900392 | 0.000753 | 0.008294 |
| Cox8b | 1.625006 | 6.772969 | 0.000754 | 0.008294 |
| Wnt3 | 1.928069 | 4.454819 | 0.000762 | 0.00836 |
| Ly6g6d | 4.317629 | 4.294593 | 0.000763 | 0.008365 |
| Rab38 | 1.836652 | 4.091859 | 0.00077 | 0.00843 |
| Krt10 | 2.812052 | 9.866325 | 0.000773 | 0.008452 |
| Efcc1 | 2.588274 | 2.034306 | 0.000776 | 0.008472 |
| Acsl1 | 1.481143 | 7.573998 | 0.000776 | 0.008472 |
| Bnip3 | 1.425887 | 6.216075 | 0.000777 | 0.008472 |
| Ltc4s | 2.579115 | 1.389985 | 0.000783 | 0.008524 |
| Tmem30b | 1.990995 | 2.556668 | 0.000784 | 0.008531 |
| Adamtsl3 | 1.668487 | 4.111846 | 0.000787 | 0.008552 |
| Sox21 | 3.138169 | 2.037849 | 0.000802 | 0.008686 |
| AC111231.1 | 2.934292 | 0.683203 | 0.000803 | 0.00869 |
| AABR07012329.1 | 2.82057 | 9.133382 | 0.000806 | 0.008711 |
| Zim1 | 1.481121 | 3.823229 | 0.000806 | 0.008711 |
| Vsig8 | 6.404613 | 6.729722 | 0.000808 | 0.008727 |
| Pkp1 | 3.493433 | 6.044319 | 1.66E-05 | 0.008801 |
| Serpinb8 | 1.381189 | 5.261526 | 0.000819 | 0.008811 |
| Fmo3 | 2.319937 | 1.72839 | 0.000827 | 0.008887 |
| Hdac11 | 1.659859 | 2.888147 | 0.000835 | 0.008952 |
| Krtap12-2 | 4.941865 | 2.180485 | 0.00084 | 0.008991 |
| Glb1l2 | 1.931954 | 4.187152 | 0.000845 | 0.009044 |
| RGD1562029 | 2.118963 | 3.69257 | 0.00085 | 0.009084 |
| Map7 | 1.737624 | 5.468294 | 0.00085 | 0.009084 |
| Skint8 | 2.445321 | 1.926631 | 0.000866 | 0.009198 |
| Btnl9 | 1.820945 | 2.734796 | 0.000876 | 0.009295 |
| Crispld1 | 2.97124 | 0.611023 | 0.000881 | 0.009337 |
| Dkk2 | 1.9377 | 3.188723 | 0.000883 | 0.009348 |
| Plaat1 | 1.744852 | 5.469759 | 0.000886 | 0.009372 |
| Dlx1 | 5.033462 | 2.656932 | 0.000947 | 0.009893 |
| Klc3 | 2.034662 | 3.249614 | 0.000948 | 0.009893 |
| Jup | 1.573731 | 8.366504 | 0.000981 | 0.010191 |
| LOC100361793 | 5.488327 | 2.640336 | 0.00099 | 0.010266 |
| Stmn4 | 2.032134 | 1.798663 | 0.000991 | 0.010275 |
| Calhm4 | 5.001006 | 3.444624 | 0.001001 | 0.010361 |
| Fmo1 | 1.711653 | 3.581958 | 0.001003 | 0.010365 |
| Krtap17-1 | 5.740757 | 2.379889 | 0.001015 | 0.010472 |
| Efna5 | 2.318828 | 2.502767 | 0.001023 | 0.010537 |
| Egr3 | 1.984067 | 4.979489 | 0.001037 | 0.010652 |
| Trim59 | 1.755125 | 5.169137 | 0.001047 | 0.010743 |
| Zfp711 | 2.166466 | 2.167616 | 0.001061 | 0.010881 |
| Ca14 | 1.700251 | 5.632841 | 0.001062 | 0.010886 |
| Trhde | 2.045304 | 2.36758 | 0.001067 | 0.010923 |
| B3galt1 | 2.301296 | 1.619074 | 0.001072 | 0.010963 |
| Actbl2 | 5.449966 | 2.994029 | 0.001075 | 0.01099 |
| Bves | 1.692011 | 4.853823 | 0.001083 | 0.011033 |
| Igfbp2 | 2.148519 | 1.887149 | 0.001095 | 0.01112 |
| RGD1563060 | 2.510673 | 4.169873 | 0.001098 | 0.01113 |
| Sun2 | 1.420205 | 7.13176 | 0.001104 | 0.011181 |
| Lypd6 | 2.834329 | 0.977464 | 0.001118 | 0.01129 |
| Krtap19-5 | 5.524538 | 1.650561 | 0.001139 | 0.011436 |
| Fads2 | 1.917725 | 4.449846 | 0.001141 | 0.011436 |
| Ephx1 | 1.53418 | 2.967105 | 0.001139 | 0.011436 |
| AABR07030521.1 | 2.591992 | 2.305148 | 0.00116 | 0.011575 |
| Pamr1 | 2.443846 | 1.614018 | 0.001161 | 0.01158 |
| Hr | 1.786068 | 7.274402 | 0.001174 | 0.011679 |
| AABR07028488.1 | 1.574995 | 4.136394 | 0.001175 | 0.011679 |
| Nectin1 | 2.082702 | 7.129589 | 0.001178 | 0.011695 |
| Acsm3 | 2.96326 | 3.505802 | 0.00118 | 0.011706 |
| Myh14 | 1.511429 | 6.98754 | 0.001217 | 0.011974 |
| LOC690386 | 5.474622 | 1.608086 | 0.001225 | 0.012029 |
| C1qtnf9 | 2.120185 | 1.489463 | 0.001232 | 0.012071 |
| Sptssb | 3.464656 | 1.241378 | 0.001254 | 0.012243 |
| AABR07053830.1 | 2.219297 | 1.226671 | 0.001255 | 0.012248 |
| Acss1 | 1.756142 | 5.70345 | 0.001265 | 0.012335 |
| Rhbdl2 | 2.190605 | 1.435743 | 0.001279 | 0.012446 |
| Grb7 | 1.597806 | 3.167211 | 0.001279 | 0.012446 |
| Lrrc39 | 1.469814 | 5.223518 | 0.001291 | 0.012549 |
| Krtap16-5 | 5.335927 | 2.621883 | 0.001294 | 0.012555 |
| Negr1 | 3.101648 | 0.885281 | 0.001296 | 0.012568 |
| Lgi2 | 1.719184 | 4.365216 | 0.001301 | 0.012592 |
| RGD1307461 | 1.636583 | 5.367879 | 0.001333 | 0.012815 |
| Spsb4 | 1.945345 | 3.351261 | 0.001356 | 0.01299 |
| Myl3 | 2.846083 | 5.423693 | 0.001365 | 0.013055 |
| Ptch2 | 2.808178 | 4.086001 | 0.001383 | 0.013217 |
| Adamts17 | 2.104844 | 2.446003 | 0.001394 | 0.013284 |
| Cd24 | 1.378799 | 5.241444 | 0.001393 | 0.013284 |
| Padi3 | 5.113952 | 5.754715 | 0.001411 | 0.013403 |
| Dclk3 | 2.974684 | 4.117746 | 0.001421 | 0.013496 |
| Ptprv | 2.408118 | 4.005359 | 0.001425 | 0.013516 |
| LOC103694398 | 5.477874 | 2.663384 | 0.001433 | 0.013563 |
| Hunk | 3.941612 | 3.618436 | 0.001449 | 0.013695 |
| AC126641.1 | 2.157089 | 2.2451 | 0.001456 | 0.013738 |
| Rptn | 2.152664 | 2.259689 | 0.001463 | 0.013794 |
| Itgbl1 | 1.553175 | 5.208279 | 0.001472 | 0.013862 |
| Foxc1 | 1.556639 | 3.764308 | 0.001479 | 0.013901 |
| Krt83 | 6.905643 | 8.474309 | 0.001485 | 0.013949 |
| RGD1565462 | 4.733991 | 0.750147 | 0.001511 | 0.014175 |
| Ppfia3 | 1.384169 | 4.345935 | 0.001517 | 0.014212 |
| Clmn | 1.656781 | 4.077571 | 0.001545 | 0.014432 |
| Adam11 | 1.506532 | 3.776594 | 0.001546 | 0.014435 |
| Fam83h | 1.744092 | 6.27475 | 0.00156 | 0.014531 |
| Nrk | 1.742202 | 3.494597 | 0.00156 | 0.014531 |
| Foxe1 | 4.380914 | 3.916081 | 0.001564 | 0.014561 |
| Defb1 | 2.181704 | 1.985986 | 0.001573 | 0.014598 |
| Tc2n | 1.573081 | 2.66672 | 0.001571 | 0.014598 |
| Tbx1 | 2.117677 | 3.539988 | 0.00158 | 0.014658 |
| Eepd1 | 1.396265 | 5.680285 | 0.001584 | 0.014685 |
| Pak3 | 1.547524 | 3.194793 | 0.001589 | 0.014722 |
| Mlana | 5.251325 | 1.144567 | 0.0016 | 0.014782 |
| Ptch1 | 1.923962 | 6.198522 | 0.001619 | 0.014934 |
| AABR07050283.1 | 2.096736 | 1.390322 | 0.001621 | 0.014941 |
| Otub2 | 1.519796 | 3.769868 | 0.001636 | 0.015046 |
| Acot13 | 1.503393 | 4.117089 | 0.00164 | 0.015081 |
| Pi16 | 1.44709 | 5.977911 | 0.001649 | 0.015135 |
| Dhtkd1 | 2.248369 | 1.580908 | 0.001665 | 0.01525 |
| F13a1 | 1.39921 | 6.717105 | 0.001691 | 0.015442 |
| Mfap2 | 1.585487 | 2.775057 | 0.001699 | 0.015483 |
| AABR07012314.1 | 2.479572 | 3.172104 | 0.001701 | 0.015487 |
| Krtap26-1 | 5.860751 | 4.922672 | 0.001705 | 0.015504 |
| Ros1 | 2.250059 | 2.10021 | 0.001711 | 0.015539 |
| Wnk2 | 1.513322 | 7.04261 | 0.001719 | 0.015592 |
| Crnn | 4.649803 | 3.731563 | 0.001741 | 0.015759 |
| Ryr3 | 1.5845 | 5.399947 | 0.001746 | 0.015769 |
| Syngr1 | 1.655773 | 2.784874 | 0.001754 | 0.015813 |
| Slc6a2 | 2.121248 | 2.805606 | 0.00176 | 0.01583 |
| Krtap11-1 | 6.085631 | 6.437532 | 0.001775 | 0.015904 |
| AABR07058422.1 | 1.846006 | 4.398752 | 0.001775 | 0.015904 |
| AABR07015055.2 | 4.617983 | 0.920475 | 0.001809 | 0.016141 |
| Pdcd4 | 1.583893 | 7.081771 | 0.001821 | 0.016203 |
| Pdzd9 | 2.148967 | 1.17904 | 0.001832 | 0.016277 |
| Lmntd2 | 2.437196 | 0.813348 | 0.00187 | 0.016588 |
| Hydin | 3.199491 | 1.220032 | 0.001872 | 0.016597 |
| Hnrnpr | 5.172771 | 6.99802 | 5.44E-05 | 0.016663 |
| Hal | 2.355815 | 5.279109 | 0.001906 | 0.016857 |
| AABR07006042.1 | 5.179924 | 1.882055 | 0.001917 | 0.016926 |
| Asb18 | 2.270562 | 1.814081 | 0.001919 | 0.016933 |
| Sult1d1 | 3.733152 | 1.495636 | 0.001924 | 0.016969 |
| Asb15 | 1.996967 | 3.115208 | 0.001969 | 0.017314 |
| Stmn1 | 1.706696 | 5.538982 | 0.001982 | 0.017382 |
| Mss51 | 1.514718 | 5.213591 | 0.001983 | 0.017382 |
| Cybrd1 | 1.396371 | 4.87348 | 0.001982 | 0.017382 |
| Shc3 | 2.6848 | 0.908275 | 0.001986 | 0.017396 |
| Cldn10 | 3.633093 | 0.665205 | 0.001999 | 0.017478 |
| Pou2f3 | 1.714644 | 4.508178 | 0.002015 | 0.017579 |
| Wfikkn2 | 2.289282 | 1.981265 | 0.002031 | 0.017708 |
| Krtap8-1 | 5.930328 | 5.450416 | 0.002065 | 0.017938 |
| Ptprf | 1.972912 | 8.351724 | 0.00208 | 0.018043 |
| Dgcr2 | 3.298445 | 1.0483 | 0.002081 | 0.018043 |
| Rasef | 2.247786 | 1.794849 | 0.002103 | 0.0182 |
| Erbb2 | 1.46012 | 4.633468 | 0.002111 | 0.018234 |
| Hpd | 2.167586 | 2.525521 | 0.002133 | 0.018387 |
| Btbd11 | 1.4213 | 4.197338 | 0.002156 | 0.01855 |
| Ggct | 1.411119 | 5.321169 | 0.002167 | 0.018628 |
| Akr1c3 | 2.815349 | 1.335302 | 0.002177 | 0.018673 |
| Susd2 | 1.946278 | 4.580206 | 0.002203 | 0.018849 |
| AABR07006049.1 | 5.603964 | 3.578463 | 0.002214 | 0.018917 |
| AABR07030469.2 | 5.035875 | 1.758009 | 0.002223 | 0.018968 |
| Klf15 | 1.736413 | 4.532551 | 0.002224 | 0.018968 |
| Bmp8a | 3.50281 | 2.54196 | 0.002237 | 0.01904 |
| Ackr4 | 1.837302 | 2.936533 | 0.002251 | 0.019093 |
| Lad1 | 1.744322 | 6.476244 | 0.002279 | 0.019313 |
| Efnb1 | 1.749691 | 5.655156 | 0.002288 | 0.019358 |
| Sbsn | 1.710747 | 7.397276 | 0.002287 | 0.019358 |
| Fzd1 | 1.629596 | 5.882855 | 0.002318 | 0.019561 |
| Hprt1 | 3.99207 | 1.90578 | 0.002341 | 0.019744 |
| Krt82 | 5.603314 | 6.241949 | 0.002347 | 0.019783 |
| Krtap14 | 5.502663 | 4.704946 | 0.002357 | 0.019827 |
| Cpm | 2.350334 | 5.620328 | 0.002364 | 0.019863 |
| Fbxo40 | 1.704453 | 6.723478 | 0.002402 | 0.020081 |
| Krtap13-1 | 5.497629 | 4.684973 | 0.002421 | 0.020181 |
| Kctd1 | 1.500276 | 4.583879 | 0.002433 | 0.020267 |
| Clcn4 | 1.559814 | 4.572049 | 0.002438 | 0.020292 |
| Mpzl2 | 1.532292 | 5.356163 | 0.002441 | 0.020302 |
| Bhlha9 | 5.30542 | 1.187519 | 0.002452 | 0.020379 |
| RGD1305347 | 2.450792 | 0.941254 | 0.002458 | 0.020416 |
| Il36b | 2.471627 | 2.323965 | 0.002464 | 0.020442 |
| Fbp2 | 1.646766 | 5.635928 | 0.002473 | 0.020506 |
| Tchh | 5.905184 | 10.54265 | 0.002494 | 0.020632 |
| Gjb2 | 3.320728 | 7.856564 | 0.002525 | 0.020872 |
| Abca8 | 1.837256 | 3.167379 | 0.00253 | 0.0209 |
| Cldn23 | 2.317959 | 1.041573 | 0.002538 | 0.020938 |
| Slc27a6 | 2.273276 | 2.899944 | 0.002545 | 0.020986 |
| Slc16a7 | 2.62494 | 3.978337 | 0.002561 | 0.02108 |
| AABR07033579.3 | 5.153894 | 2.648033 | 0.002577 | 0.021168 |
| Ptn | 1.709368 | 4.366857 | 0.00261 | 0.021427 |
| Cwh43 | 1.550807 | 3.831121 | 0.002646 | 0.021668 |
| LOC680442 | 5.201089 | 1.104462 | 0.002649 | 0.02168 |
| Ar | 1.386692 | 5.674174 | 0.002654 | 0.021702 |
| Slc2a4 | 1.719375 | 7.630723 | 0.002661 | 0.021738 |
| Zfp266 | 1.426868 | 6.886302 | 0.002665 | 0.021755 |
| Asb2 | 1.37934 | 6.454216 | 0.00267 | 0.021782 |
| Meox1 | 2.308781 | 4.638125 | 0.000117 | 0.021899 |
| Gm37863 | 2.047746 | 4.553566 | 0.000119 | 0.021899 |
| Nccrp1 | 1.590871 | 4.212851 | 0.002695 | 0.021932 |
| Kcnk4 | 2.044109 | 1.482684 | 0.002728 | 0.022157 |
| Stk26 | 2.405013 | 3.753426 | 0.002738 | 0.02222 |
| Ccdc155 | 1.756563 | 3.091861 | 0.002742 | 0.022233 |
| Neu2 | 1.604642 | 5.903979 | 0.002791 | 0.022511 |
| Slc2a12 | 1.507343 | 4.61512 | 0.002792 | 0.022511 |
| Atp6v1d | 2.295607 | 4.966277 | 0.00014 | 0.022589 |
| Gm26769 | 1.868609 | 4.707776 | 0.000149 | 0.022777 |
| Nos1 | 1.843546 | 5.716265 | 0.002839 | 0.022842 |
| LOC100910851 | 4.090518 | 1.108186 | 0.002844 | 0.022852 |
| Alpg | 2.358231 | 0.90835 | 0.002851 | 0.022886 |
| Prxl2a | 1.419111 | 3.972445 | 0.00285 | 0.022886 |
| St8sia6 | 2.642881 | 0.641167 | 0.002861 | 0.022935 |
| Sema6c | 1.482328 | 6.337898 | 0.002862 | 0.022935 |
| Aspg | 1.740733 | 2.735535 | 0.002871 | 0.022982 |
| Mylk4 | 3.251016 | 0.770388 | 0.002874 | 0.022983 |
| Cenpf | 1.584839 | 5.89285 | 0.002916 | 0.023264 |
| Tspan18 | 1.70578 | 3.551499 | 0.00294 | 0.02342 |
| Csdc2 | 3.125474 | 3.494242 | 0.002948 | 0.023467 |
| Scn10a | 2.830284 | 0.827806 | 0.002963 | 0.023576 |
| Gsdma | 1.838274 | 4.905446 | 0.002996 | 0.023762 |
| Syde2 | 1.68792 | 3.477691 | 0.003012 | 0.023848 |
| Krt5 | 1.553675 | 9.368836 | 0.003021 | 0.023902 |
| Coq10a | 1.437725 | 6.343504 | 0.003026 | 0.02393 |
| Them4 | 1.867062 | 3.620773 | 0.003066 | 0.024206 |
| Cbx2 | 1.811812 | 4.143235 | 0.003066 | 0.024206 |
| Tdh | 3.741471 | 1.426561 | 0.003075 | 0.024236 |
| Phkg1 | 1.827905 | 6.075443 | 0.003093 | 0.02433 |
| AC099183.4 | 5.094187 | 1.020665 | 0.003104 | 0.024399 |
| NEWGENE_1310561 | 4.646622 | 2.080863 | 0.003119 | 0.024503 |
| AABR07012310.1 | 2.307098 | 2.508305 | 0.00312 | 0.024503 |
| LOC108348118 | 3.721026 | 1.666321 | 0.003164 | 0.024793 |
| Fermt1 | 1.807586 | 4.035623 | 0.003171 | 0.024821 |
| Dok5 | 1.521619 | 3.133307 | 0.003178 | 0.024831 |
| Has3 | 1.716888 | 4.916628 | 0.00319 | 0.024913 |
| Uox | 1.596804 | 3.218946 | 0.003198 | 0.024952 |
| RGD1564571 | 2.264747 | 1.257704 | 0.00322 | 0.025092 |
| LOC100911486 | 2.120038 | 1.771563 | 0.003221 | 0.025092 |
| Ggh | 1.475008 | 3.029645 | 0.003221 | 0.025092 |
| Gata6 | 2.411044 | 1.15629 | 0.003237 | 0.02516 |
| Dpp6 | 2.024347 | 1.830206 | 0.003246 | 0.025214 |
| Txlnb | 1.606434 | 7.52893 | 0.003251 | 0.025238 |
| RGD1306271 | 1.36683 | 4.127778 | 0.003281 | 0.025395 |
| LOC691485 | 2.11457 | 1.455677 | 0.003292 | 0.025468 |
| Col7a1 | 1.547723 | 6.242553 | 0.003312 | 0.025603 |
| LOC684762 | 2.003088 | 5.267816 | 0.003322 | 0.025658 |
| Fam89a | 1.666603 | 2.101356 | 0.00333 | 0.025692 |
| Lor | 2.360116 | 3.446789 | 0.003347 | 0.025807 |
| Trpc3 | 1.922011 | 1.821908 | 0.003366 | 0.025884 |
| AABR07021544.1 | 1.383688 | 4.672624 | 0.003374 | 0.025933 |
| Adgrg1 | 1.690375 | 6.290453 | 0.003378 | 0.025948 |
| Tpi1 | 1.564448 | 4.636249 | 0.003393 | 0.026025 |
| AABR07072853.5 | 1.734315 | 6.197786 | 0.003416 | 0.026155 |
| LOC100365588 | 4.665216 | 2.948009 | 0.003419 | 0.026167 |
| Pinlyp | 2.563246 | 2.761138 | 0.00345 | 0.026318 |
| AABR07031489.1 | 2.101211 | 1.787754 | 0.003445 | 0.026318 |
| Rassf9 | 1.504705 | 3.915765 | 0.003447 | 0.026318 |
| Edar | 1.796101 | 1.966282 | 0.003461 | 0.026348 |
| Sirpb3 | 2.318331 | 1.416587 | 0.003465 | 0.026367 |
| Spink5 | 4.272123 | 2.330229 | 0.003481 | 0.026436 |
| Clstn3 | 2.090148 | 1.622467 | 0.003482 | 0.026436 |
| St14 | 1.989389 | 5.789494 | 0.003523 | 0.026678 |
| Nfe2l3 | 1.592395 | 3.98689 | 0.003525 | 0.026684 |
| AABR07072002.1 | 4.660855 | 1.443853 | 0.003539 | 0.026731 |
| Spz1 | 1.842723 | 4.343884 | 0.000206 | 0.0268 |
| Krt78 | 2.007095 | 5.301244 | 0.00357 | 0.026927 |
| Cxadr | 1.643457 | 3.872345 | 0.003595 | 0.027054 |
| Kcnmb4 | 3.315145 | 0.553415 | 0.003599 | 0.027062 |
| Hspa2 | 1.895877 | 4.80247 | 0.003626 | 0.027218 |
| AABR07009834.1 | 2.249963 | 1.548527 | 0.00364 | 0.027269 |
| Dsc2 | 2.585739 | 3.053109 | 0.003644 | 0.027283 |
| Dnase1l2 | 2.062214 | 3.421236 | 0.003679 | 0.027488 |
| Ca6 | 1.556597 | 2.633316 | 0.003683 | 0.027488 |
| Tnnc1 | 2.486614 | 4.183413 | 0.003685 | 0.027493 |
| Lzts1 | 2.92567 | 4.158757 | 0.003688 | 0.027498 |
| Cyp39a1 | 1.949179 | 1.883089 | 0.003713 | 0.027658 |
| Krt26 | 5.266212 | 4.793409 | 0.003728 | 0.027741 |
| Flrt3 | 1.719379 | 4.843866 | 0.003732 | 0.027753 |
| Defb22 | 1.731209 | 4.397667 | 0.000234 | 0.027959 |
| Sdr42e1 | 2.060911 | 2.488254 | 0.003794 | 0.028117 |
| Gm13511 | 1.85605 | 4.648403 | 0.000251 | 0.02826 |
| AL954855.1 | 1.814502 | 4.315677 | 0.000245 | 0.02826 |
| St8sia5 | 1.894615 | 2.686124 | 0.003836 | 0.028357 |
| Tnfrsf14 | 2.277082 | 4.945784 | 0.000271 | 0.028383 |
| Fsd2 | 1.921647 | 6.31537 | 0.003868 | 0.02852 |
| Gm15161 | 1.685949 | 4.296328 | 0.000283 | 0.028624 |
| Ptk7 | 1.803342 | 5.037513 | 0.003893 | 0.028678 |
| B3gnt8 | 2.083376 | 2.27969 | 0.003916 | 0.028815 |
| RGD1560730 | 2.566573 | 3.053165 | 0.003924 | 0.028855 |
| Epdr1 | 1.397532 | 4.739318 | 0.00397 | 0.02915 |
| Kcnk3 | 1.82147 | 1.80881 | 0.003974 | 0.029153 |
| AABR07045485.1 | 1.624848 | 6.230236 | 0.003974 | 0.029153 |
| Trmt9b | 2.306131 | 0.696095 | 0.00404 | 0.029559 |
| Marveld2 | 1.94106 | 2.364684 | 0.004093 | 0.029907 |
| Krtap31-1 | 4.593293 | 1.345573 | 0.004116 | 0.03 |
| A930016O22Rik | 2.21666 | 5.683194 | 0.000327 | 0.030001 |
| Fn3k | 2.245376 | 1.646517 | 0.004138 | 0.030126 |
| Lipk | 2.112893 | 3.230881 | 0.004142 | 0.030138 |
| Tcf7 | 2.008676 | 3.698438 | 0.004153 | 0.030196 |
| Kb23 | 6.738404 | 8.28799 | 0.004164 | 0.030254 |
| Krt86 | 6.012489 | 7.16326 | 0.00418 | 0.030341 |
| H1f5 | 1.809967 | 5.911579 | 0.004204 | 0.030486 |
| Sspn | 1.420297 | 6.014191 | 0.004252 | 0.030788 |
| Lypd5 | 1.677181 | 3.682555 | 0.004274 | 0.030928 |
| Pla2g4f | 1.540725 | 5.273366 | 0.004283 | 0.030963 |
| Dnajc6 | 2.804999 | 2.917227 | 0.004295 | 0.031038 |
| Plag1 | 1.664559 | 2.025125 | 0.004328 | 0.031258 |
| Sult5a1 | 2.451722 | 2.466449 | 0.004335 | 0.031296 |
| Mgat5 | 1.713963 | 4.749906 | 0.004356 | 0.031417 |
| Cep70 | 1.734291 | 2.795902 | 0.00439 | 0.031585 |
| C1qtnf7 | 1.683163 | 1.713135 | 0.004483 | 0.032168 |
| AABR07072181.3 | 5.014518 | 3.970657 | 0.004506 | 0.032317 |
| Rassf7 | 1.448075 | 2.710307 | 0.004513 | 0.032338 |
| C1qc | 1.454374 | 5.704616 | 0.00456 | 0.03259 |
| Plekhg1 | 1.791163 | 6.175004 | 0.004617 | 0.032886 |
| Ttc22 | 1.380694 | 2.977202 | 0.004615 | 0.032886 |
| Fam167a | 2.126531 | 5.423099 | 0.004643 | 0.033043 |
| Catsperz | 1.683373 | 2.373406 | 0.004653 | 0.033096 |
| AABR07067099.1 | 2.25516 | 0.666666 | 0.004672 | 0.033174 |
| Gprasp2 | 1.857367 | 2.148362 | 0.004679 | 0.033198 |
| Krtap7-1 | 5.691738 | 6.603399 | 0.004684 | 0.033201 |
| Tm4sf4 | 3.257231 | 1.524126 | 0.004694 | 0.033238 |
| Mrgprb3 | 1.620833 | 2.703717 | 0.004701 | 0.033257 |
| Nim1k | 4.191654 | 2.451943 | 0.004745 | 0.033495 |
| AABR07005535.1 | 2.307114 | 0.980671 | 0.004801 | 0.033771 |
| AC132057.1 | 4.701269 | 0.724965 | 0.004846 | 0.034035 |
| Slc4a1 | 2.508939 | 1.74229 | 0.004879 | 0.034229 |
| Krt31 | 6.581675 | 9.053455 | 0.004974 | 0.034758 |
| Krtap27-1 | 4.207879 | 1.885345 | 0.004986 | 0.034812 |
| Col11a1 | 1.541504 | 3.650737 | 0.005 | 0.034878 |
| Krtap15-1 | 5.80095 | 6.585966 | 0.005073 | 0.035301 |
| Ces2h | 2.541737 | 0.812466 | 0.005081 | 0.035343 |
| Atp10b | 1.442903 | 3.58045 | 0.005167 | 0.035817 |
| Slpil3 | 3.018678 | 1.148443 | 0.005181 | 0.035877 |
| Samd12 | 1.813767 | 1.46435 | 0.005189 | 0.035904 |
| Rab7 | 2.003009 | 4.754488 | 0.000465 | 0.036083 |
| Krt27 | 6.129155 | 7.37689 | 0.005274 | 0.036437 |
| Ces1e | 1.931107 | 4.806246 | 0.000503 | 0.036602 |
| Espn | 1.963133 | 1.939163 | 0.005343 | 0.036792 |
| Igsf10 | 1.611685 | 4.410318 | 0.00051 | 0.036917 |
| Krtap1-5 | 4.638036 | 3.652266 | 0.005431 | 0.037271 |
| Cpa3 | 1.464859 | 4.334932 | 0.005429 | 0.037271 |
| Micalcl | 1.885331 | 3.649477 | 0.00552 | 0.037778 |
| Ctnnd2 | 2.081328 | 4.705231 | 0.00057 | 0.037986 |
| Rnf39 | 1.522391 | 3.821904 | 0.005673 | 0.038536 |
| Hoxb2 | 2.359084 | 1.660675 | 0.005751 | 0.038987 |
| Krt39 | 4.239473 | 2.565014 | 0.005787 | 0.039177 |
| Hdc | 1.985425 | 4.391329 | 0.00064 | 0.039396 |
| Stfa3 | 1.98009 | 3.191404 | 0.005851 | 0.03952 |
| LOC680428 | 4.587653 | 0.643332 | 0.005887 | 0.039692 |
| LOC685544 | 4.587653 | 0.643332 | 0.005884 | 0.039692 |
| Efcab12 | 2.606247 | 5.516976 | 0.000661 | 0.039812 |
| Gm12628 | 1.959299 | 5.167702 | 0.000657 | 0.039812 |
| AABR07027752.2 | 2.716069 | 2.427783 | 0.00591 | 0.039824 |
| Gpr37 | 2.585349 | 2.293962 | 0.005919 | 0.03985 |
| Rtl3 | 2.164298 | 0.918384 | 0.005932 | 0.03992 |
| Tmem25 | 1.98753 | 0.94527 | 0.005941 | 0.039939 |
| Krt73 | 5.104327 | 7.478859 | 0.005996 | 0.040237 |
| Tnmd | 2.146878 | 1.40213 | 0.006014 | 0.040326 |
| Rnf208 | 1.970772 | 2.414812 | 0.006015 | 0.040326 |
| AABR07027581.1 | 2.677798 | 1.950961 | 0.006043 | 0.040475 |
| Ephb1 | 2.331713 | 2.880907 | 0.006041 | 0.040475 |
| RGD1561916 | 1.892109 | 1.987206 | 0.006084 | 0.040654 |
| Ripk4 | 1.625751 | 4.322357 | 0.006093 | 0.040678 |
| Krtap2-1 | 4.01482 | 1.369377 | 0.006109 | 0.04077 |
| Zfp296 | 1.8083 | 1.403416 | 0.006121 | 0.040831 |
| Wnt6 | 2.455087 | 2.603708 | 0.006153 | 0.040967 |
| Pax1 | 2.706134 | 0.64179 | 0.006381 | 0.042166 |
| Sumf2 | 2.04465 | 5.870496 | 0.000836 | 0.042181 |
| Ovol1 | 1.498185 | 6.284414 | 0.006397 | 0.042201 |
| Acsm5 | 2.274414 | 1.264791 | 0.006411 | 0.042279 |
| Nox1 | 2.143792 | 0.927369 | 0.006456 | 0.042537 |
| RGD1310935 | 1.746141 | 3.010658 | 0.006469 | 0.042559 |
| Ephx3 | 1.719048 | 3.366024 | 0.006467 | 0.042559 |
| Mc2r | 1.927626 | 1.151608 | 0.006477 | 0.042563 |
| Gcat | 1.605619 | 2.574228 | 0.006472 | 0.042563 |
| Cryl1 | 1.593542 | 2.17317 | 0.006478 | 0.042563 |
| Ube2ql1 | 1.820048 | 1.712746 | 0.006495 | 0.042601 |
| Aip | 1.894796 | 4.915425 | 0.000873 | 0.042727 |
| Ankrd35 | 1.487242 | 4.574898 | 0.006551 | 0.042897 |
| Stx4a | 1.42906 | 4.19315 | 0.000899 | 0.042934 |
| Tulp1 | 1.843259 | 1.738042 | 0.006576 | 0.043011 |
| Dlx4 | 3.421161 | 1.523216 | 0.006611 | 0.043219 |
| Fzd5 | 2.509218 | 3.817076 | 0.00662 | 0.043261 |
| AABR07044404.1 | 2.148903 | 3.584684 | 0.00664 | 0.043287 |
| Slc45a3 | 1.434057 | 2.689233 | 0.006631 | 0.043287 |
| Irx2 | 1.576761 | 4.33504 | 0.006757 | 0.043937 |
| Nemp2 | 1.45613 | 4.2654 | 0.000972 | 0.044301 |
| LOC100362366 | 1.899593 | 1.617478 | 0.006846 | 0.044415 |
| Grin3a | 2.218991 | 5.499363 | 0.00099 | 0.04448 |
| Cenpp | 1.81278 | 4.611062 | 0.000991 | 0.04448 |
| Elmod1 | 2.075096 | 3.647995 | 0.006876 | 0.044537 |
| Cct6a | 2.659287 | 6.072823 | 0.001008 | 0.044817 |
| Traj21 | 2.539502 | 5.880321 | 0.001011 | 0.044817 |
| Mif4gd | 1.845093 | 5.070863 | 0.001002 | 0.044817 |
| Col12a1 | 2.432919 | 6.559291 | 0.001085 | 0.045207 |
| Gm44752 | 1.980151 | 5.332546 | 0.001076 | 0.045207 |
| Gm17322 | 1.956769 | 5.64507 | 0.001059 | 0.045207 |
| Adamts18 | 2.177985 | 2.56259 | 0.007033 | 0.045296 |
| Cdh3 | 1.756596 | 5.860057 | 0.007038 | 0.045296 |
| Zxdb | 1.819835 | 4.976724 | 0.001096 | 0.045438 |
| C330024D21Rik | 1.620976 | 4.203409 | 0.001101 | 0.045485 |
| Gpr82 | 1.908678 | 5.446674 | 0.001111 | 0.045591 |
| Gm19514 | 1.549879 | 4.47359 | 0.001117 | 0.045683 |
| Aldh1a7 | 2.192997 | 2.074176 | 0.007139 | 0.04587 |
| AABR07027811.2 | 1.778474 | 4.104582 | 0.007179 | 0.046102 |
| Pkib | 1.533166 | 2.299322 | 0.007201 | 0.046205 |
| Krt13 | 5.184832 | 7.498217 | 0.001171 | 0.046765 |
| Pla2g2e | 3.904031 | 1.282062 | 0.007326 | 0.046858 |
| Sult2b1 | 1.470823 | 3.630159 | 0.007379 | 0.047016 |
| Epha4 | 2.172098 | 4.975234 | 0.001227 | 0.04727 |
| Taf1b | 2.048219 | 5.091668 | 0.001224 | 0.04727 |
| Rnf222 | 2.047502 | 2.726931 | 0.007437 | 0.047301 |
| Tdrd12 | 1.835425 | 4.746091 | 0.001249 | 0.047467 |
| Hdgfl3 | 1.727794 | 4.485696 | 0.001269 | 0.047648 |
| Stk33 | 2.256335 | 6.464397 | 0.001302 | 0.04827 |
| Klhl33 | 1.371667 | 4.558887 | 0.007634 | 0.048341 |
| Ppara | 1.430115 | 2.391797 | 0.007652 | 0.048398 |
| Alkbh3 | 1.601789 | 4.748171 | 0.001311 | 0.048459 |
| Slc7a15 | 2.031859 | 0.832067 | 0.007695 | 0.048602 |
| Cyp1a1 | 2.907791 | 0.820633 | 0.007722 | 0.048704 |
| Tpsb2 | 1.556565 | 2.838448 | 0.007795 | 0.0491 |
| Gm48309 | 1.533194 | 4.661225 | 0.001348 | 0.049217 |
| Rnf112 | 2.023669 | 1.115388 | 0.007854 | 0.049388 |
| AABR07001905.1 | 2.339216 | 1.560794 | 0.007966 | 0.049997 |
| Gpd1 | 1.368034 | 7.932068 | 0.008031 | 0.050261 |
| Gm26594 | 1.657945 | 4.851392 | 0.001408 | 0.050491 |
| Retn | 1.909437 | 1.998418 | 0.008102 | 0.050659 |
| Stra6 | 3.712864 | 3.900406 | 0.008142 | 0.050841 |
| AABR07008030.1 | 1.801794 | 2.408299 | 0.008212 | 0.051194 |
| Dupd1 | 1.544234 | 3.109399 | 0.008257 | 0.051362 |
| Msi1 | 2.568467 | 3.545786 | 0.008268 | 0.051391 |
| Rnf43 | 1.691428 | 3.12664 | 0.008303 | 0.051548 |
| Csmd1 | 1.640464 | 1.71664 | 0.008331 | 0.051627 |
| Elf5 | 2.349287 | 2.280587 | 0.008351 | 0.051703 |
| E130201H02Rik | 2.474579 | 4.629312 | 0.001504 | 0.051731 |
| A030005L19Rik | 1.942857 | 5.077703 | 0.001491 | 0.051731 |
| Harbi1 | 1.503328 | 4.411432 | 0.001487 | 0.051731 |
| Tprg1 | 1.706591 | 4.326263 | 0.008429 | 0.052054 |
| Gm8520 | 1.774182 | 4.599452 | 0.001554 | 0.052077 |
| Arl15 | 1.448356 | 4.270521 | 0.008603 | 0.052904 |
| Fam131b | 1.398554 | 2.896019 | 0.008616 | 0.052964 |
| Id4 | 1.667024 | 3.454494 | 0.008676 | 0.053147 |
| Ldb3 | 1.638574 | 9.721869 | 0.008675 | 0.053147 |
| 2700081O15Rik | 2.380073 | 5.807631 | 0.001659 | 0.053516 |
| Gm29241 | 1.718599 | 5.237543 | 0.001663 | 0.053516 |
| Fgd3 | 1.677999 | 5.185118 | 0.001657 | 0.053516 |
| Hebp2 | 1.59393 | 2.319798 | 0.008825 | 0.053945 |
| Gm31084 | 1.826961 | 4.854621 | 0.001691 | 0.053978 |
| Krt81 | 5.447636 | 6.761276 | 0.008854 | 0.054104 |
| Aknad1 | 3.229098 | 0.973542 | 0.008875 | 0.05421 |
| AABR07048211.1 | 1.987714 | 1.189972 | 0.008991 | 0.054698 |
| Tesc | 2.052879 | 2.687764 | 0.009098 | 0.055198 |
| Gdpd2 | 2.409524 | 4.300398 | 0.009139 | 0.055401 |
| Lap3 | 1.840059 | 7.44073 | 0.009155 | 0.055429 |
| Tmem102 | 2.188813 | 1.436989 | 0.009168 | 0.055473 |
| Proser2 | 2.062675 | 2.616845 | 0.00918 | 0.055483 |
| Hist1h2bo | 1.612576 | 4.689227 | 0.009241 | 0.055713 |
| LOC103689983 | 4.27316 | 1.416028 | 0.009287 | 0.055923 |
| Gm14218 | 2.08434 | 5.516539 | 0.001778 | 0.055958 |
| Gm14097 | 1.949 | 5.196389 | 0.001792 | 0.056008 |
| Slc9a2 | 2.277747 | 5.977596 | 0.001834 | 0.056729 |
| Gm4654 | 2.396594 | 5.263409 | 0.00185 | 0.056787 |
| Akr1c19 | 1.64325 | 1.674842 | 0.009461 | 0.056827 |
| Kcnj15 | 1.760817 | 2.570939 | 0.009514 | 0.057076 |
| Dmkn | 1.60892 | 7.483743 | 0.009534 | 0.057104 |
| Trappc6a | 1.41019 | 2.378923 | 0.009551 | 0.057175 |
| Ecrg4 | 2.1424 | 1.293349 | 0.009577 | 0.057287 |
| Akr1e2 | 1.448585 | 2.449476 | 0.009612 | 0.057429 |
| Klhl38 | 1.514544 | 5.357648 | 0.00965 | 0.057627 |
| AABR07033590.1 | 3.694295 | 1.226925 | 0.009713 | 0.05791 |
| Ccdc106 | 1.93437 | 4.97548 | 0.00195 | 0.058049 |
| Tgm6 | 3.587072 | 3.333997 | 0.009763 | 0.058163 |
| Sparc | 2.502541 | 5.346204 | 0.001971 | 0.058362 |
| Ttc21a | 2.418884 | 1.384254 | 0.009807 | 0.058377 |
| Calml4 | 1.736988 | 2.228664 | 0.009942 | 0.059033 |
| Lrrc56 | 1.507956 | 1.914108 | 0.009969 | 0.059135 |
| Sfn | 1.595714 | 6.532276 | 0.009981 | 0.059169 |
| AABR07044301.1 | 1.942648 | 1.652477 | 0.010006 | 0.059268 |
| Aspm | 1.439522 | 5.5624 | 0.010111 | 0.059718 |
| Wnk4 | 1.61844 | 1.920485 | 0.010274 | 0.060412 |
| A230103J11Rik | 1.580158 | 4.410904 | 0.002082 | 0.060756 |
| Thrap3 | 2.431135 | 6.702718 | 0.002115 | 0.06093 |
| Prune1 | 2.335122 | 6.318524 | 0.002123 | 0.06093 |
| Il31ra | 1.787428 | 1.89447 | 0.010399 | 0.060971 |
| Myom1 | 1.386676 | 8.90151 | 0.010456 | 0.061205 |
| Gm49876 | 1.834069 | 4.93393 | 0.00216 | 0.061383 |
| Mtmr10 | 1.427431 | 6.330859 | 0.010522 | 0.061497 |
| AABR07032724.1 | 1.811663 | 2.871605 | 0.010557 | 0.061625 |
| Bmp6 | 1.438014 | 2.045388 | 0.010709 | 0.062413 |
| Myoz3 | 1.421515 | 5.421506 | 0.01075 | 0.062575 |
| Gmppa | 2.30577 | 5.959429 | 0.002263 | 0.062725 |
| Slc7a1 | 1.708758 | 6.176193 | 0.010793 | 0.062759 |
| Gm23645 | 2.120282 | 5.976447 | 0.002292 | 0.062831 |
| LOC100363136 | 4.476138 | 3.219133 | 0.010831 | 0.062934 |
| AABR07034362.2 | 1.408292 | 3.440173 | 0.010834 | 0.062934 |
| Tex13b | 1.4677 | 4.772792 | 0.002321 | 0.062966 |
| Psrc1 | 2.087902 | 1.839204 | 0.011029 | 0.06384 |
| LOC108348235 | 3.815166 | 1.981589 | 0.011037 | 0.063858 |
| Fndc3c1 | 2.425433 | 5.177411 | 0.002467 | 0.064292 |
| Hoxd9 | 2.196762 | 5.656657 | 0.002466 | 0.064292 |
| Aldh18a1 | 1.898791 | 4.915328 | 0.002425 | 0.064292 |
| Med21 | 1.753407 | 5.459487 | 0.002472 | 0.064292 |
| Fam168a | 1.753203 | 5.070276 | 0.002452 | 0.064292 |
| Syn2 | 1.678054 | 4.706458 | 0.002457 | 0.064292 |
| Ndufa4l2 | 1.375385 | 2.789387 | 0.011165 | 0.064368 |
| Ap5s1 | 2.103455 | 4.898095 | 0.002495 | 0.064391 |
| Tex46 | 2.027524 | 5.171634 | 0.00249 | 0.064391 |
| Trim37 | 1.427359 | 4.40432 | 0.00249 | 0.064391 |
| AC120949.4 | 2.084814 | 0.693211 | 0.011209 | 0.064508 |
| Fam180a | 1.884696 | 0.76691 | 0.011253 | 0.064721 |
| Wwc2 | 1.523834 | 4.480843 | 0.002534 | 0.064786 |
| Asgr1 | 1.991182 | 5.640987 | 0.002553 | 0.064856 |
| Pcbp3 | 2.402226 | 6.143722 | 0.00264 | 0.065074 |
| Gm15602 | 1.689155 | 4.887447 | 0.002625 | 0.065074 |
| Sinhcaf | 1.539041 | 4.984971 | 0.002643 | 0.065074 |
| Rpl10a-ps2 | 1.381808 | 4.061634 | 0.002644 | 0.065074 |
| Krtap1-3 | 4.218824 | 2.503806 | 0.011338 | 0.065086 |
| Myo5c | 3.378688 | 2.536415 | 0.011339 | 0.065086 |
| RGD1564899 | 1.36958 | 7.518753 | 0.011372 | 0.065252 |
| Gm13898 | 1.967504 | 5.069256 | 0.002682 | 0.065279 |
| LOC100912348 | 3.679961 | 0.803588 | 0.011457 | 0.065531 |
| Gja1 | 2.313232 | 9.764425 | 0.011779 | 0.067106 |
| Zg16 | 1.8997 | 5.634492 | 0.002897 | 0.067472 |
| Tsga13 | 1.727133 | 5.020691 | 0.002912 | 0.0675 |
| Vwa2 | 1.427616 | 2.771157 | 0.011959 | 0.067948 |
| Mogat2 | 1.689045 | 2.238993 | 0.011979 | 0.068006 |
| Frem3 | 3.487393 | 0.529546 | 0.012039 | 0.068269 |
| Sptssa | 1.618776 | 5.113134 | 0.002966 | 0.06845 |
| Nmrk2 | 1.381899 | 5.647369 | 0.012203 | 0.069062 |
| Gprc5d | 3.308335 | 5.054015 | 0.012273 | 0.069378 |
| Dlx5 | 2.110961 | 0.880504 | 0.012337 | 0.069709 |
| Ccdc150 | 1.680915 | 4.95944 | 0.003077 | 0.069846 |
| Gm4131 | 1.628676 | 4.7575 | 0.003066 | 0.069846 |
| Hspa1b | 1.545572 | 1.81836 | 0.012458 | 0.070177 |
| 5031415H12Rik | 2.753361 | 6.86257 | 0.003238 | 0.07125 |
| Slc1a3 | 1.592329 | 4.629638 | 0.012851 | 0.072026 |
| Calcoco1 | 2.857954 | 1.254498 | 0.012914 | 0.072238 |
| Tpd52l1 | 1.766391 | 2.649868 | 0.012921 | 0.072248 |
| Gm7740 | 1.695819 | 4.735289 | 0.003389 | 0.072336 |
| Hrob | 1.663165 | 4.885829 | 0.003409 | 0.072336 |
| Gm49254 | 1.643959 | 4.638612 | 0.003355 | 0.072336 |
| Asb10 | 1.42494 | 4.488383 | 0.012948 | 0.072346 |
| Mettl21c | 1.511844 | 2.667804 | 0.01296 | 0.072386 |
| Traj20 | 2.27059 | 5.50582 | 0.003433 | 0.072404 |
| Cd244a | 1.713016 | 5.3269 | 0.003439 | 0.072404 |
| CT030184.1 | 2.017776 | 6.108344 | 0.003475 | 0.072524 |
| Gm13397 | 1.896064 | 5.841125 | 0.003491 | 0.072725 |
| Antxr1 | 1.862636 | 5.022853 | 0.003509 | 0.072726 |
| Gm13777 | 2.228279 | 6.562886 | 0.003524 | 0.072783 |
| Gm14161 | 1.7048 | 4.796487 | 0.003519 | 0.072783 |
| Gm24475 | 1.884063 | 5.290051 | 0.003541 | 0.073019 |
| Lman1l | 2.061842 | 6.489384 | 0.003567 | 0.073169 |
| Crabp2 | 2.200272 | 6.703179 | 0.003576 | 0.073218 |
| Ccne2 | 2.062978 | 5.855396 | 0.003614 | 0.073408 |
| Abhd4 | 1.526407 | 4.501335 | 0.00363 | 0.073458 |
| Zfp786 | 1.651879 | 1.876196 | 0.013224 | 0.073544 |
| Slc36a2 | 1.561192 | 1.685519 | 0.01331 | 0.073769 |
| Usp26 | 2.186683 | 4.799564 | 0.003698 | 0.074071 |
| Ccl22 | 1.667108 | 1.797924 | 0.013492 | 0.07461 |
| Serpinb11 | 2.285314 | 1.016059 | 0.013554 | 0.074866 |
| Gm15564 | 1.589179 | 4.54214 | 0.003755 | 0.074955 |
| LOC501406 | 1.381674 | 1.939969 | 0.013595 | 0.075032 |
| Gvin-ps2 | 1.6775 | 4.749439 | 0.003802 | 0.075145 |
| Trpt1 | 1.707898 | 4.644552 | 0.003817 | 0.075315 |
| Kif11 | 2.214379 | 5.36745 | 0.003881 | 0.075758 |
| Krtap3-3 | 3.6973 | 2.533357 | 0.013775 | 0.075791 |
| LOC102549061 | 1.458132 | 5.417071 | 0.013907 | 0.076196 |
| Gm12919 | 2.456945 | 5.339705 | 0.004034 | 0.077402 |
| Nudt17 | 1.975603 | 5.410928 | 0.004039 | 0.077402 |
| Grpel2 | 1.951393 | 5.421323 | 0.004127 | 0.077673 |
| 9030204H09Rik | 1.566454 | 4.871811 | 0.004103 | 0.077673 |
| Prss35 | 1.860928 | 1.107871 | 0.014445 | 0.078324 |
| Cxcl9 | 1.527849 | 3.060161 | 0.014486 | 0.078456 |
| Traj17 | 1.988707 | 5.245655 | 0.004232 | 0.078568 |
| Ube2o | 2.05655 | 7.088835 | 0.004248 | 0.078625 |
| Gm15023 | 1.519463 | 4.690538 | 0.004259 | 0.0787 |
| Lpar2 | 2.221526 | 0.850188 | 0.014568 | 0.078811 |
| Skint10 | 2.106711 | 1.416589 | 0.014597 | 0.078942 |
| Dach1 | 1.385077 | 3.977911 | 0.014738 | 0.079501 |
| 1110028F11Rik | 1.462787 | 4.468243 | 0.004334 | 0.079836 |
| Krtap3-1 | 4.315796 | 4.568454 | 0.014873 | 0.080046 |
| Fzd3 | 1.859532 | 3.490191 | 0.014929 | 0.080227 |
| Cmss1 | 1.472593 | 3.007078 | 0.014951 | 0.080317 |
| Cldn3 | 2.174911 | 1.062791 | 0.015042 | 0.080683 |
| Krcc1 | 2.502261 | 5.542244 | 0.00442 | 0.080685 |
| Fam83c | 1.747125 | 5.560651 | 0.004443 | 0.080861 |
| Gm5600 | 1.416876 | 4.617523 | 0.004464 | 0.080994 |
| Dnah5 | 1.724481 | 4.191101 | 0.004539 | 0.081277 |
| Gm11464 | 1.695501 | 5.791947 | 0.004525 | 0.081277 |
| Klhl21 | 1.424919 | 4.372701 | 0.004548 | 0.081277 |
| Cmtm8 | 1.956287 | 1.498861 | 0.015244 | 0.081524 |
| Ankrd29 | 1.568052 | 1.684523 | 0.015241 | 0.081524 |
| Gm44284 | 2.427979 | 5.754961 | 0.004576 | 0.081652 |
| Oxld1 | 1.479304 | 1.500774 | 0.015283 | 0.081676 |
| Adam22 | 1.55842 | 4.964349 | 0.004597 | 0.081677 |
| Fat1 | 1.396487 | 9.374709 | 0.015315 | 0.081786 |
| Erbb4 | 2.128396 | 0.650177 | 0.015364 | 0.082019 |
| Gm36804 | 1.401006 | 4.338418 | 0.004649 | 0.082098 |
| Rdh19 | 1.483803 | 4.454102 | 0.004662 | 0.082213 |
| Tex9 | 1.812365 | 5.790195 | 0.004707 | 0.082429 |
| Gm2011 | 1.673565 | 5.360153 | 0.004702 | 0.082429 |
| Ndufaf6 | 1.474866 | 5.027674 | 0.004743 | 0.082515 |
| Gm42842 | 1.440384 | 4.910353 | 0.004779 | 0.082811 |
| Krt20 | 3.712099 | 6.118394 | 0.004802 | 0.082931 |
| Brsk1 | 1.495093 | 4.609983 | 0.004807 | 0.082931 |
| Ciita | 1.607323 | 4.36672 | 0.004831 | 0.083119 |
| Ippk | 2.189857 | 5.784122 | 0.004895 | 0.083133 |
| Cnih1 | 1.938969 | 5.97317 | 0.004965 | 0.083133 |
| Gm10392 | 1.890195 | 5.549678 | 0.004889 | 0.083133 |
| Gm9745 | 1.627324 | 5.043619 | 0.004961 | 0.083133 |
| Gm16184 | 1.577511 | 5.115563 | 0.004925 | 0.083133 |
| Gm39302 | 1.405128 | 4.62599 | 0.004929 | 0.083133 |
| Tnnt1 | 2.372704 | 4.998925 | 0.005026 | 0.08356 |
| Ndufs3 | 1.667551 | 5.042581 | 0.005018 | 0.08356 |
| Col3a1 | 2.276147 | 7.632376 | 0.005043 | 0.083615 |
| Ubap1l | 2.77523 | 6.530893 | 0.005093 | 0.08363 |
| Tmem30a | 2.203126 | 7.088953 | 0.005063 | 0.08363 |
| Med8 | 1.890038 | 6.218948 | 0.0051 | 0.08363 |
| Gm10193 | 1.530014 | 4.90383 | 0.00508 | 0.08363 |
| Ddx1 | 1.711513 | 5.667902 | 0.005125 | 0.08374 |
| Klk5 | 1.86833 | 3.254972 | 0.015754 | 0.083746 |
| Pkhd1l1 | 1.382861 | 3.135794 | 0.015836 | 0.084102 |
| Myom2 | 2.572896 | 6.822348 | 0.005204 | 0.08425 |
| Trim6 | 2.336824 | 5.236497 | 0.005189 | 0.08425 |
| Nkx2-5 | 1.936864 | 5.305263 | 0.005191 | 0.08425 |
| Gm46355 | 1.901499 | 5.640474 | 0.005187 | 0.08425 |
| Gm16130 | 1.767835 | 5.408491 | 0.005246 | 0.084296 |
| Gm9298 | 1.454655 | 4.61235 | 0.005236 | 0.084296 |
| Slc30a1 | 1.949551 | 6.787007 | 0.01597 | 0.084693 |
| Wnt16 | 1.923641 | 4.657503 | 0.005321 | 0.084929 |
| Klhl3 | 1.976556 | 7.128156 | 0.005437 | 0.085521 |
| Krt75 | 3.171545 | 5.047879 | 0.005482 | 0.085961 |
| Gm11551 | 2.464165 | 5.807674 | 0.005509 | 0.086081 |
| Hist1h3b | 1.719215 | 3.981756 | 0.016388 | 0.086434 |
| Alkbh5 | 1.737343 | 5.502111 | 0.005592 | 0.086466 |
| Gm48859 | 1.987337 | 6.851534 | 0.005603 | 0.086534 |
| Cdhr3 | 1.892991 | 0.908001 | 0.016473 | 0.086851 |
| AC130146.1 | 1.370207 | 4.470115 | 0.016633 | 0.087408 |
| Cysrt1 | 1.520271 | 1.87963 | 0.016713 | 0.087765 |
| AC141521.1 | 2.930975 | 0.55565 | 0.016781 | 0.087864 |
| Jag1 | 1.551888 | 7.602263 | 0.01681 | 0.087952 |
| Ptger3 | 1.467531 | 2.084687 | 0.016851 | 0.088104 |
| Gm38014 | 1.578475 | 5.031529 | 0.005835 | 0.088208 |
| Ybx2 | 1.730933 | 5.439032 | 0.00589 | 0.088663 |
| Pank2 | 1.760233 | 5.355684 | 0.005901 | 0.08872 |
| C1qtnf3 | 2.020093 | 0.961974 | 0.017035 | 0.088904 |
| Cd300lg | 1.57423 | 2.47578 | 0.017201 | 0.089557 |
| Luc7l3 | 1.756867 | 4.885189 | 0.005997 | 0.089596 |
| Art4 | 1.92522 | 2.13336 | 0.01725 | 0.089704 |
| B3gat3 | 1.484582 | 4.846502 | 0.006023 | 0.089771 |
| Tmem200a | 3.12239 | 0.984662 | 0.017293 | 0.08985 |
| LOC103689971 | 2.981809 | 2.353482 | 0.017296 | 0.08985 |
| Gm10913 | 1.777849 | 5.012422 | 0.006049 | 0.090034 |
| Rpl32 | 1.988655 | 4.86674 | 0.006066 | 0.090052 |
| Gm10840 | 1.601313 | 5.087364 | 0.006074 | 0.090052 |
| Krt34 | 4.699995 | 6.139782 | 0.017345 | 0.09007 |
| Gm43689 | 2.611471 | 5.969173 | 0.006114 | 0.090221 |
| Cfap300 | 1.658407 | 4.266701 | 0.006138 | 0.090353 |
| Glipr1l3 | 1.429193 | 4.455623 | 0.006138 | 0.090353 |
| Clps | 1.761916 | 5.060792 | 0.006339 | 0.090848 |
| Mccc1os | 1.504807 | 5.179849 | 0.006336 | 0.090848 |
| Tmem132c | 1.91297 | 1.364639 | 0.017585 | 0.091118 |
| LOC102552128 | 1.73536 | 1.135372 | 0.017614 | 0.091235 |
| Abca9 | 1.80068 | 5.373498 | 0.006408 | 0.091284 |
| Maged1 | 1.469564 | 4.650456 | 0.006415 | 0.091284 |
| Gm34059 | 2.242619 | 5.162064 | 0.006442 | 0.091408 |
| Calhm6 | 1.708619 | 5.79387 | 0.006475 | 0.091589 |
| AC114363.1 | 1.920654 | 0.837363 | 0.017747 | 0.091682 |
| Eps8l1 | 1.554015 | 6.037448 | 0.01775 | 0.091682 |
| Sema6a | 1.864063 | 5.254305 | 0.006515 | 0.091928 |
| Wnt5b | 2.097634 | 0.900265 | 0.017832 | 0.091933 |
| AABR07055288.1 | 1.810369 | 1.111926 | 0.017854 | 0.091956 |
| Itgae | 1.784412 | 5.082498 | 0.006593 | 0.092066 |
| Gm49616 | 1.662986 | 5.607078 | 0.006611 | 0.092066 |
| Gm20535 | 1.581271 | 5.500334 | 0.006622 | 0.092066 |
| Ttbk2 | 1.498231 | 5.210158 | 0.006603 | 0.092066 |
| F2 | 1.375533 | 4.520628 | 0.006588 | 0.092066 |
| Trh | 2.129514 | 1.012067 | 0.017965 | 0.092332 |
| Slc5a11 | 2.128032 | 0.920389 | 0.018012 | 0.092505 |
| Fbxo39 | 2.037161 | 6.594519 | 0.006671 | 0.092606 |
| Mcoln3 | 2.46083 | 2.260258 | 0.018059 | 0.092682 |
| Slfn10-ps | 1.980152 | 5.203536 | 0.006718 | 0.09295 |
| Hspa12b | 1.649549 | 5.968735 | 0.006727 | 0.09295 |
| Gm5641 | 2.249749 | 5.668168 | 0.006775 | 0.093178 |
| B230312C02Rik | 1.95786 | 5.397265 | 0.006781 | 0.093178 |
| Rab11fip4os2 | 1.512826 | 5.314949 | 0.006781 | 0.093178 |
| LOC100912564 | 1.408025 | 5.118712 | 0.018303 | 0.093667 |
| Gjd2 | 2.043118 | 5.254047 | 0.006854 | 0.093949 |
| Phyhip | 1.406771 | 5.009375 | 0.006895 | 0.094071 |
| Krt84 | 2.997318 | 5.52891 | 0.006993 | 0.094248 |
| Slc17a7 | 2.030726 | 6.698699 | 0.007026 | 0.094248 |
| Tnfrsf19 | 2.026429 | 6.381703 | 0.006971 | 0.094248 |
| Enox1 | 1.640966 | 5.608209 | 0.006991 | 0.094248 |
| Gm37522 | 1.516367 | 5.060407 | 0.007018 | 0.094248 |
| Rhpn2 | 1.706754 | 2.257195 | 0.018517 | 0.094568 |
| Scarf1 | 2.149349 | 6.14831 | 0.007068 | 0.094612 |
| Clcn1 | 1.430138 | 6.154237 | 0.018673 | 0.095225 |
| Gm4651 | 1.742396 | 6.216486 | 0.007153 | 0.095309 |
| Sar1a | 2.229689 | 6.203898 | 0.007193 | 0.095512 |
| Uckl1 | 2.08608 | 5.100351 | 0.007225 | 0.095512 |
| Ccz1 | 1.409972 | 4.660867 | 0.007211 | 0.095512 |
| Prkaca | 2.056151 | 7.029921 | 0.007283 | 0.096065 |
| AC111804.2 | 2.075882 | 0.528774 | 0.018932 | 0.096386 |
| Adgrg2 | 1.829597 | 0.736172 | 0.018942 | 0.096393 |
| Smim24 | 2.425096 | 1.072409 | 0.018952 | 0.096408 |
| Tmem169 | 1.452417 | 4.708426 | 0.007343 | 0.096545 |
| Fam91a1 | 2.343679 | 5.684451 | 0.007367 | 0.096755 |
| Dstyk | 1.856861 | 5.861547 | 0.007421 | 0.097031 |
| Tmem35a | 2.108234 | 0.575683 | 0.019157 | 0.097215 |
| Efna4 | 1.427024 | 2.482294 | 0.019194 | 0.097351 |
| Mr1 | 1.511239 | 1.451402 | 0.019301 | 0.097674 |
| Gm25432 | 1.450027 | 4.694783 | 0.007568 | 0.098294 |
| Mtif2 | 1.509989 | 4.756872 | 0.007596 | 0.098452 |
| Mt4 | 1.549414 | 4.630938 | 0.007635 | 0.098514 |
| Arhgef17 | 1.482827 | 4.842955 | 0.007642 | 0.098514 |
| Ranbp17 | 2.078033 | 7.491685 | 0.007774 | 0.098626 |
| Ap2a2 | 1.937651 | 6.837835 | 0.007707 | 0.098626 |
| Grm6 | 1.832771 | 5.507201 | 0.007741 | 0.098626 |
| Colq | 2.095328 | 6.800926 | 0.007813 | 0.098881 |
| Ap1m2 | 1.851567 | 5.46793 | 0.007826 | 0.098881 |
| LOC100910814 | 3.161538 | 0.737776 | 0.019615 | 0.098936 |
| Asic4 | 1.7965 | 4.988388 | 0.00788 | 0.098947 |
| Ankrd9 | 1.501188 | 4.827923 | 0.007893 | 0.098947 |
| Gm12473 | 1.461724 | 4.732228 | 0.007899 | 0.098947 |
| Clptm1 | 1.532939 | 4.952788 | 0.007932 | 0.098988 |
| BC055324 | 1.600821 | 5.144951 | 0.007998 | 0.099257 |
| Bambi | 1.763599 | 4.83069 | 0.00803 | 0.099341 |
| Gm5921 | 1.43656 | 4.525565 | 0.00805 | 0.099481 |
| Itgb2l | 2.160255 | 5.594111 | 0.008126 | 0.10003 |
| Cep112it | 1.777275 | 5.249076 | 0.00817 | 0.10003 |
| Snu13 | 1.434875 | 4.780432 | 0.008137 | 0.10003 |
| 4931422A03Rik | 1.380663 | 4.553839 | 0.008161 | 0.10003 |
| Gm13746 | 1.621765 | 5.665872 | 0.008357 | 0.101489 |
| Gm19174 | 1.786757 | 6.031735 | 0.008422 | 0.101967 |
| Krt14 | 3.26868 | 5.720338 | 0.00845 | 0.101997 |
| Ftx | 2.035928 | 6.215056 | 0.00845 | 0.101997 |
| Gm49478 | 1.613641 | 5.565677 | 0.008572 | 0.102681 |
| Arc | 2.140608 | 4.964471 | 0.008659 | 0.10317 |
| Arap1 | 2.007347 | 5.917989 | 0.008676 | 0.103268 |
| Ankmy1 | 1.618948 | 0.975728 | 0.020819 | 0.10333 |
| Spon2 | 1.612067 | 5.940939 | 0.008696 | 0.103396 |
| Gm38020 | 1.838079 | 4.701922 | 0.008757 | 0.103663 |
| 4930438A08Rik | 1.368481 | 4.619712 | 0.008762 | 0.103663 |
| Gm41609 | 2.155057 | 6.255839 | 0.008785 | 0.103778 |
| Rab31 | 1.91353 | 6.928503 | 0.008839 | 0.103778 |
| Slx1b | 1.58373 | 5.481212 | 0.008854 | 0.103778 |
| Ip6k2 | 1.591868 | 5.454653 | 0.008888 | 0.10379 |
| Anapc13 | 2.687059 | 6.296825 | 0.009083 | 0.104479 |
| Atcay | 1.731052 | 5.675628 | 0.00904 | 0.104479 |
| Tmem258 | 1.590135 | 5.148959 | 0.009038 | 0.104479 |
| Arnt | 1.544215 | 5.568837 | 0.009025 | 0.104479 |
| 6820408C15Rik | 1.534983 | 5.336014 | 0.009068 | 0.104479 |
| Susd5 | 1.399233 | 4.95608 | 0.009099 | 0.104479 |
| Gm12967 | 1.850433 | 6.176394 | 0.009171 | 0.104971 |
| Gm15787 | 1.640418 | 4.694187 | 0.009168 | 0.104971 |
| Dnah7c | 1.927431 | 5.712042 | 0.009284 | 0.105661 |
| Sugp1 | 1.767004 | 9.313995 | 0.009283 | 0.105661 |
| Amd-ps3 | 1.73143 | 4.854394 | 0.009346 | 0.105661 |
| Nr2e3 | 1.496958 | 5.13641 | 0.009329 | 0.105661 |
| Gm20681 | 2.174507 | 8.112064 | 0.009576 | 0.107038 |
| Rrm1 | 2.184322 | 6.689703 | 0.009608 | 0.107222 |
| Ndufa11 | 1.898281 | 6.580386 | 0.009723 | 0.107596 |
| Gfpt2 | 1.578307 | 5.525655 | 0.009751 | 0.107596 |
| Echdc2 | 1.512933 | 4.781817 | 0.009713 | 0.107596 |
| Ttc39a | 1.369208 | 5.191889 | 0.009702 | 0.107596 |
| Crip1 | 1.670765 | 5.61232 | 0.009799 | 0.107683 |
| Tmem233 | 1.398421 | 3.792104 | 0.022053 | 0.108052 |
| Mxd1 | 1.995199 | 5.780878 | 0.00987 | 0.108104 |
| Gm11267 | 2.023177 | 6.895098 | 0.009893 | 0.108128 |
| Dio3 | 2.521881 | 2.020047 | 0.022122 | 0.108243 |
| Snai3 | 1.464684 | 2.569321 | 0.022168 | 0.108356 |
| Ass1 | 2.291858 | 5.627184 | 0.010025 | 0.108402 |
| Olfr371 | 2.290393 | 7.217394 | 0.009983 | 0.108402 |
| Gm15943 | 2.082403 | 6.107118 | 0.01002 | 0.108402 |
| Prpf19 | 1.679797 | 5.719481 | 0.009999 | 0.108402 |
| Dars | 1.382739 | 4.367515 | 0.009951 | 0.108402 |
| Cxcr3 | 1.748259 | 4.831566 | 0.010106 | 0.108788 |
| Gm6344 | 1.536333 | 5.068025 | 0.010136 | 0.108858 |
| Gm15806 | 1.451088 | 4.759582 | 0.010138 | 0.108858 |
| Fcnb | 2.73951 | 8.437132 | 0.010242 | 0.109368 |
| Tp73 | 1.548971 | 2.53148 | 0.022508 | 0.109465 |
| AC099183.3 | 3.239426 | 2.391744 | 0.022547 | 0.109619 |
| Col5a1 | 2.251028 | 7.002315 | 0.010371 | 0.109673 |
| Gm26511 | 1.724726 | 5.970508 | 0.010372 | 0.109673 |
| Prkcb | 1.471714 | 5.362837 | 0.010331 | 0.109673 |
| Nphp1 | 1.368128 | 4.99469 | 0.010458 | 0.110001 |
| Krt79 | 2.823842 | 6.348772 | 0.010513 | 0.110038 |
| Tipin | 1.449113 | 5.182685 | 0.010545 | 0.110038 |
| Gm12611 | 1.374337 | 5.080108 | 0.010521 | 0.110038 |
| Lrrc8e | 1.554286 | 3.031006 | 0.022726 | 0.110449 |
| AABR07012295.1 | 2.195021 | 1.234163 | 0.022843 | 0.110881 |
| Osgin2 | 1.664123 | 5.847079 | 0.010699 | 0.111064 |
| Chrna10 | 1.497408 | 5.089887 | 0.010774 | 0.111153 |
| 4930505N22Rik | 1.612483 | 4.676612 | 0.01081 | 0.111249 |
| Plppr5 | 1.547499 | 1.306945 | 0.023106 | 0.111659 |
| Gm4756 | 1.564643 | 4.67051 | 0.010964 | 0.111681 |
| Gm40999 | 1.449862 | 4.962758 | 0.010958 | 0.111681 |
| Fcrl2 | 1.369903 | 3.13092 | 0.023263 | 0.112305 |
| Raly | 1.73392 | 7.324035 | 0.011115 | 0.112373 |
| Car2 | 1.393715 | 2.404108 | 0.023381 | 0.112689 |
| Krt15 | 2.982486 | 5.243357 | 0.011188 | 0.112759 |
| Fam111a | 1.980441 | 5.103588 | 0.023432 | 0.112815 |
| Hs6st3 | 1.912472 | 1.082913 | 0.023454 | 0.112815 |
| Npas4 | 1.604514 | 5.444241 | 0.011242 | 0.113156 |
| Osbpl11 | 1.774264 | 6.693155 | 0.011295 | 0.113407 |
| B4galnt4 | 1.626295 | 3.066295 | 0.02362 | 0.1135 |
| Ninj2 | 1.788599 | 1.314066 | 0.02364 | 0.113524 |
| Cdk2ap2 | 1.935229 | 5.967147 | 0.011405 | 0.113556 |
| Ttll11 | 1.601499 | 5.607594 | 0.011412 | 0.113556 |
| Gm17022 | 1.520989 | 5.489744 | 0.011399 | 0.113556 |
| Cfap97d2 | 1.452591 | 5.294603 | 0.011427 | 0.113556 |
| Gm14245 | 1.371611 | 4.288525 | 0.011459 | 0.113675 |
| Gm7464 | 2.299005 | 5.74707 | 0.011502 | 0.113951 |
| Btnl4 | 2.167723 | 6.669605 | 0.011545 | 0.114188 |
| Gm40655 | 1.915698 | 6.430905 | 0.011669 | 0.114846 |
| Gm30042 | 1.524573 | 4.730658 | 0.011666 | 0.114846 |
| Gm20705 | 1.434146 | 4.933216 | 0.011642 | 0.114846 |
| AC099089.1 | 1.592879 | 2.509478 | 0.024039 | 0.114991 |
| Ntng1 | 1.759292 | 1.186565 | 0.024062 | 0.114992 |
| Huwe1 | 1.58686 | 5.936936 | 0.011743 | 0.115024 |
| Gm44036 | 1.485898 | 4.309944 | 0.011712 | 0.115024 |
| Gm6937 | 1.37787 | 4.891023 | 0.011758 | 0.115024 |
| Necap2 | 2.532125 | 4.525714 | 0.011787 | 0.115036 |
| Gm8130 | 1.955812 | 6.622902 | 0.011821 | 0.115036 |
| Hspg2 | 1.601806 | 5.559329 | 0.011811 | 0.115036 |
| Gm49011 | 1.477041 | 5.013854 | 0.011833 | 0.115036 |
| Gm12981 | 1.459946 | 5.005027 | 0.011897 | 0.115074 |
| Prdx1 | 1.403611 | 5.162784 | 0.011909 | 0.115074 |
| Dusp14 | 1.384007 | 2.289542 | 0.024152 | 0.115292 |
| Rnaset2b | 1.873877 | 5.627624 | 0.011984 | 0.11552 |
| LOC680396 | 3.126926 | 0.740458 | 0.024262 | 0.115628 |
| Rnf227 | 1.418159 | 1.98172 | 0.024392 | 0.116082 |
| Gm28375 | 1.605695 | 5.855927 | 0.012183 | 0.116467 |
| Gm11548 | 1.394039 | 4.681281 | 0.012195 | 0.116467 |
| 4933405E24Rik | 1.813486 | 5.127106 | 0.012246 | 0.11674 |
| Lrrc38 | 1.619359 | 1.996854 | 0.02462 | 0.116989 |
| 1700003G18Rik | 1.371669 | 4.532305 | 0.012306 | 0.117155 |
| Wdcp | 1.7159 | 6.043108 | 0.012486 | 0.118083 |
| Aldh3b3 | 1.578834 | 6.054449 | 0.012465 | 0.118083 |
| Glod5 | 1.730927 | 6.286037 | 0.012546 | 0.118214 |
| Gm34885 | 1.499946 | 4.937932 | 0.012575 | 0.118294 |
| Usp13 | 1.860618 | 5.254928 | 0.012683 | 0.118465 |
| Lcn10 | 1.804992 | 6.307444 | 0.012655 | 0.118465 |
| Akt2 | 1.801692 | 6.125616 | 0.012634 | 0.118465 |
| Esco2 | 1.377347 | 2.843419 | 0.025063 | 0.118548 |
| Gm12351 | 1.993726 | 4.911877 | 0.012774 | 0.118951 |
| Mtrex | 1.87962 | 5.576058 | 0.012888 | 0.119358 |
| Bspry | 1.660937 | 5.910436 | 0.012853 | 0.119358 |
| Spata31 | 1.495329 | 5.316129 | 0.012842 | 0.119358 |
| Gm4750 | 1.85671 | 5.03498 | 0.013002 | 0.120044 |
| Zfp2 | 1.754313 | 5.71589 | 0.013039 | 0.120205 |
| Mir3109 | 1.858549 | 5.878864 | 0.013088 | 0.120468 |
| Gm49181 | 1.50818 | 4.728895 | 0.013117 | 0.120546 |
| Gm41555 | 1.38489 | 4.690078 | 0.01315 | 0.120604 |
| Plb1 | 1.583727 | 1.878718 | 0.025734 | 0.120696 |
| Nckap5 | 1.370978 | 3.896064 | 0.025763 | 0.120755 |
| Mrpl13 | 2.112567 | 6.560758 | 0.01322 | 0.12103 |
| Zfp791 | 1.620337 | 6.033234 | 0.013213 | 0.12103 |
| Tnks2 | 2.471655 | 1.015872 | 0.025872 | 0.121147 |
| Izumo1r | 1.450739 | 5.22878 | 0.01326 | 0.121199 |
| Gm29585 | 1.640697 | 5.888096 | 0.013296 | 0.1212 |
| Gm14051 | 1.371189 | 4.488963 | 0.013316 | 0.1212 |
| Gm23116 | 1.700616 | 4.798948 | 0.013335 | 0.121206 |
| Antxr2 | 1.580186 | 4.800447 | 0.013341 | 0.121206 |
| Dchs1 | 2.051797 | 5.689233 | 0.013499 | 0.121845 |
| Lpar4 | 1.567578 | 5.446346 | 0.013512 | 0.121845 |
| Gm48752 | 1.422037 | 4.987208 | 0.013496 | 0.121845 |
| Acp2 | 1.704276 | 5.298262 | 0.013556 | 0.12204 |
| Cdkn2b | 1.49742 | 3.990573 | 0.026426 | 0.122922 |
| Rnf181 | 1.43362 | 5.338678 | 0.013794 | 0.123346 |
| AABR07000411.1 | 1.495824 | 1.290157 | 0.026587 | 0.123538 |
| Scn1a | 1.876188 | 0.94011 | 0.026608 | 0.123555 |
| Pcdhgb8 | 1.686605 | 7.13587 | 0.013919 | 0.123723 |
| Asxl1 | 1.54819 | 6.065723 | 0.01394 | 0.123822 |
| Syt9 | 1.625459 | 1.605521 | 0.026707 | 0.123942 |
| Cdkl3 | 1.501233 | 5.100043 | 0.013987 | 0.124148 |
| Kbtbd3 | 1.816279 | 5.861768 | 0.014018 | 0.124333 |
| Flg | 2.955414 | 6.813308 | 0.014107 | 0.124574 |
| Tnfsf15 | 1.566332 | 1.142545 | 0.0269 | 0.124635 |
| Lpxn | 1.582379 | 5.760998 | 0.014167 | 0.124752 |
| Rb1 | 1.485588 | 5.225314 | 0.014149 | 0.124752 |
| Slc5a10 | 1.419045 | 2.246253 | 0.027017 | 0.124774 |
| 9830107B12Rik | 2.172079 | 5.404183 | 0.014198 | 0.12481 |
| Gm29372 | 1.670355 | 5.572408 | 0.014213 | 0.124856 |
| Sntb1 | 1.694943 | 5.694662 | 0.014274 | 0.125024 |
| Glis2 | 1.478468 | 4.848631 | 0.014335 | 0.125038 |
| Sectm1a | 1.839265 | 5.849993 | 0.014407 | 0.125354 |
| Krtap4-3 | 3.658024 | 3.522133 | 0.027222 | 0.125604 |
| Chl1 | 2.180912 | 6.142299 | 0.014489 | 0.125718 |
| Gm26583 | 1.901747 | 5.8395 | 0.014497 | 0.125718 |
| Cfap410 | 2.361056 | 4.393922 | 0.014656 | 0.125764 |
| Fxyd1 | 2.111062 | 6.694694 | 0.014546 | 0.125764 |
| Scaf1 | 1.826689 | 6.729497 | 0.014642 | 0.125764 |
| Ddx25 | 1.77453 | 5.720709 | 0.014648 | 0.125764 |
| Krt88 | 1.709115 | 4.959642 | 0.014616 | 0.125764 |
| Glod4 | 1.493444 | 5.611641 | 0.014664 | 0.125764 |
| Gm9991 | 1.410852 | 5.244191 | 0.014597 | 0.125764 |
| Cap2 | 1.573465 | 6.112975 | 0.014739 | 0.126044 |
| Gm3807 | 1.625128 | 5.810947 | 0.014768 | 0.126107 |
| A2ml1 | 1.99017 | 6.035133 | 0.014813 | 0.126311 |
| Gm18558 | 1.932557 | 4.761759 | 0.014889 | 0.126605 |
| Gars | 1.68931 | 6.253396 | 0.014975 | 0.126605 |
| Rgs20 | 1.645304 | 6.275982 | 0.014892 | 0.126605 |
| Col9a3 | 1.598146 | 5.937048 | 0.015053 | 0.126912 |
| D030025P21Rik | 1.417422 | 5.331851 | 0.015099 | 0.127113 |
| F10 | 1.674291 | 6.541702 | 0.015324 | 0.127926 |
| Hif1an | 1.812956 | 7.08946 | 0.015372 | 0.128065 |
| Gm18959 | 1.578893 | 5.615379 | 0.015373 | 0.128065 |
| Otop2 | 2.537423 | 0.673042 | 0.028159 | 0.128858 |
| Aldh3a1 | 1.408367 | 1.873044 | 0.028208 | 0.129 |
| LOC100910554 | 1.830335 | 1.293916 | 0.028311 | 0.129229 |
| Creld2 | 1.504543 | 5.812271 | 0.01568 | 0.129275 |
| Fbxl19 | 1.445239 | 5.608736 | 0.015704 | 0.129382 |
| Prm1 | 1.528714 | 5.228974 | 0.015792 | 0.129574 |
| Slc6a21 | 1.531314 | 5.662799 | 0.015824 | 0.129744 |
| Hist1h2bd | 1.540164 | 3.348656 | 0.028487 | 0.129785 |
| Slc23a3 | 1.400427 | 1.231323 | 0.028526 | 0.129881 |
| Msantd3 | 1.525403 | 5.311146 | 0.015872 | 0.129958 |
| Glp2r | 1.912305 | 6.239545 | 0.015908 | 0.129984 |
| Ambra1 | 1.391033 | 5.552479 | 0.015964 | 0.130103 |
| Rnf24 | 1.430532 | 5.658899 | 0.01601 | 0.130375 |
| LOC100360846 | 1.401869 | 1.497346 | 0.028697 | 0.130491 |
| Lcn12 | 1.700292 | 0.811976 | 0.028715 | 0.130495 |
| Gm46233 | 2.129945 | 5.385134 | 0.016105 | 0.130519 |
| Vstm4 | 1.908844 | 4.86568 | 0.016088 | 0.130519 |
| Gm39500 | 1.639749 | 6.30892 | 0.016443 | 0.130647 |
| Gm15764 | 1.927238 | 6.615199 | 0.016497 | 0.130942 |
| Cacna2d1 | 1.487707 | 5.170483 | 0.016518 | 0.131028 |
| Gm15484 | 1.531585 | 5.171501 | 0.016606 | 0.131241 |
| Cdc45 | 1.512289 | 4.689971 | 0.01664 | 0.131382 |
| Gm26413 | 1.401457 | 4.858697 | 0.016729 | 0.131826 |
| Gm44745 | 1.445183 | 4.745189 | 0.016761 | 0.13197 |
| Gm27254 | 1.620126 | 5.339864 | 0.016837 | 0.132045 |
| C4b | 1.504874 | 6.099122 | 0.016966 | 0.132295 |
| Gatd1 | 1.633611 | 1.398745 | 0.029388 | 0.132973 |
| Pno1 | 1.922225 | 5.914127 | 0.017178 | 0.133191 |
| Gm12185 | 1.68935 | 6.896904 | 0.017182 | 0.133191 |
| Kng1 | 2.55011 | 2.805018 | 0.029456 | 0.133238 |
| Gkap1 | 1.941079 | 7.425905 | 0.017276 | 0.133262 |
| Traf7 | 1.916885 | 6.235366 | 0.017302 | 0.133262 |
| Gm37894 | 1.847783 | 7.064705 | 0.017252 | 0.133262 |
| Btnl6 | 1.401812 | 4.908464 | 0.017289 | 0.133262 |
| Slc4a9 | 1.375438 | 1.516895 | 0.029503 | 0.133327 |
| D230025D16Rik | 1.66241 | 5.875429 | 0.017353 | 0.133478 |
| Gsg1l2 | 2.125196 | 6.622148 | 0.017485 | 0.133608 |
| Gpr156 | 2.037437 | 6.949643 | 0.017423 | 0.133608 |
| Tm4sf20 | 1.584451 | 6.017646 | 0.017535 | 0.133608 |
| Gm44822 | 1.478111 | 5.64523 | 0.017605 | 0.133608 |
| Stk32a | 1.476875 | 5.010749 | 0.017605 | 0.133608 |
| Gm37132 | 1.404357 | 5.301959 | 0.017546 | 0.133608 |
| Sirt1 | 2.539938 | 6.943831 | 0.017658 | 0.133757 |
| Cibar1 | 1.709257 | 6.781513 | 0.017799 | 0.133858 |
| Rnf223 | 1.512547 | 4.988818 | 0.017841 | 0.13387 |
| Scn8a | 1.720115 | 1.852805 | 0.029685 | 0.133979 |
| Gpr35 | 1.675255 | 6.599961 | 0.017873 | 0.133986 |
| Wdr12 | 1.507015 | 5.94477 | 0.017968 | 0.134314 |
| Gm15321 | 1.537601 | 5.699707 | 0.018063 | 0.134688 |
| Ccdc170 | 1.511356 | 1.111214 | 0.030122 | 0.135531 |
| Mettl9 | 1.870918 | 7.877749 | 0.018313 | 0.135789 |
| Ces2a | 2.068617 | 7.157328 | 0.018395 | 0.136169 |
| Gm44178 | 1.720367 | 5.470819 | 0.018539 | 0.136451 |
| Gm47746 | 1.583307 | 5.278204 | 0.018513 | 0.136451 |
| AABR07012313.1 | 1.744196 | 2.506275 | 0.030417 | 0.136477 |
| Zc2hc1c | 1.537083 | 1.891137 | 0.030508 | 0.1368 |
| Wnt10a | 1.602063 | 2.785354 | 0.030519 | 0.136808 |
| Ppp1r1b | 1.534317 | 6.143398 | 0.018657 | 0.136814 |
| Tpra1 | 1.662784 | 5.625105 | 0.0187 | 0.136894 |
| Zbtb3 | 1.537799 | 4.751367 | 0.018735 | 0.136894 |
| Gm37502 | 1.648231 | 5.531885 | 0.01889 | 0.137319 |
| Ceacam18 | 1.605248 | 6.127086 | 0.018899 | 0.137319 |
| Gm13171 | 1.58358 | 6.092597 | 0.018977 | 0.137703 |
| Gm17151 | 1.53746 | 4.801994 | 0.019044 | 0.137829 |
| Gm16982 | 1.794902 | 6.599673 | 0.019097 | 0.13792 |
| Mgl2 | 1.617854 | 5.120592 | 0.019201 | 0.138291 |
| 6330403L08Rik | 1.57373 | 6.070666 | 0.019224 | 0.138291 |
| Ep300 | 1.693565 | 6.668634 | 0.019344 | 0.138694 |
| Tmprss2 | 1.453382 | 4.700282 | 0.01938 | 0.13875 |
| Raf1 | 1.43159 | 5.802311 | 0.019386 | 0.13875 |
| Tnfaip8l3 | 1.958268 | 4.826584 | 0.019398 | 0.138751 |
| Stk11ip | 1.702273 | 6.128436 | 0.019449 | 0.138938 |
| Gm28100 | 1.607253 | 5.636754 | 0.019494 | 0.139021 |
| Casp1 | 1.39609 | 4.734102 | 0.019529 | 0.139104 |
| Zfp286 | 1.599877 | 5.003569 | 0.019666 | 0.139576 |
| Gm11788 | 1.581995 | 5.271485 | 0.019652 | 0.139576 |
| Pstpip2 | 1.372812 | 5.254624 | 0.01969 | 0.139665 |
| Gria4 | 1.446287 | 5.354631 | 0.019741 | 0.139909 |
| Nrap | 2.404664 | 7.613877 | 0.019846 | 0.140515 |
| Ube2s | 1.709643 | 6.806161 | 0.019927 | 0.140599 |
| Gm960 | 1.59498 | 5.631029 | 0.019923 | 0.140599 |
| Gucy2g | 1.479999 | 2.086235 | 0.031771 | 0.14098 |
| Psme2 | 1.664914 | 5.464416 | 0.020109 | 0.141295 |
| Krtap4-6 | 3.231567 | 6.492844 | 0.020271 | 0.141683 |
| Gm6855 | 1.650784 | 6.664853 | 0.020339 | 0.141906 |
| Prss53 | 1.718682 | 6.437778 | 0.020354 | 0.141928 |
| Zdhhc4 | 1.528397 | 5.698944 | 0.020424 | 0.142172 |
| Large2 | 1.645323 | 2.851908 | 0.032262 | 0.142388 |
| Fam3b | 1.469873 | 1.776742 | 0.032286 | 0.142436 |
| Grtp1 | 1.41731 | 1.142636 | 0.032314 | 0.142471 |
| n-R5s143 | 1.524863 | 5.95495 | 0.020492 | 0.142492 |
| Gm17851 | 2.825302 | 5.431217 | 0.020613 | 0.14258 |
| Myo1f | 1.908198 | 8.885387 | 0.020561 | 0.14258 |
| Rnf31 | 1.466833 | 5.555255 | 0.020626 | 0.14258 |
| Ern1 | 1.552096 | 4.893403 | 0.020678 | 0.142693 |
| Pgm5 | 1.571791 | 4.87946 | 0.020746 | 0.142993 |
| Tmem189 | 1.469155 | 5.343363 | 0.020773 | 0.143056 |
| Gm37761 | 1.697271 | 5.152316 | 0.020883 | 0.143341 |
| Phf11d | 1.748162 | 7.256639 | 0.021064 | 0.143707 |
| 4930481A15Rik | 1.605326 | 5.619143 | 0.021066 | 0.143707 |
| Litaf | 1.666474 | 4.896303 | 0.021182 | 0.144228 |
| Lockd | 1.750776 | 6.093538 | 0.021307 | 0.144641 |
| Polr2h | 1.740477 | 7.042212 | 0.021371 | 0.144641 |
| Kctd15 | 1.605225 | 6.718116 | 0.021373 | 0.144641 |
| Gm11954 | 1.43367 | 4.685159 | 0.021612 | 0.145516 |
| Krt90 | 2.791436 | 5.548812 | 0.021782 | 0.146435 |
| Calhm1 | 1.440447 | 5.180869 | 0.021938 | 0.146721 |
| Gm30873 | 1.877594 | 4.890319 | 0.02196 | 0.146785 |
| Mtr | 1.524084 | 6.969076 | 0.022016 | 0.146913 |
| Tspan13 | 1.406027 | 5.100939 | 0.022014 | 0.146913 |
| LOC103691744 | 2.042765 | 1.334818 | 0.033707 | 0.147226 |
| Ubap2l | 1.634874 | 6.586152 | 0.022108 | 0.14728 |
| Fer1l6 | 1.564959 | 1.235834 | 0.033776 | 0.147439 |
| Camk1d | 1.628453 | 5.227458 | 0.022207 | 0.14747 |
| Ssr2 | 1.420993 | 5.632041 | 0.022233 | 0.147471 |
| Gpsm2 | 1.439697 | 5.096703 | 0.022408 | 0.148204 |
| Krtap19-9b | 2.510602 | 5.08176 | 0.022449 | 0.148275 |
| Gm12088 | 1.647492 | 6.8457 | 0.022465 | 0.148275 |
| Mir3105 | 1.43378 | 5.01792 | 0.022516 | 0.148275 |
| Ppan | 1.953999 | 6.079873 | 0.022602 | 0.148664 |
| Ccdc28b | 1.52976 | 5.793622 | 0.02263 | 0.1487 |
| Awat2 | 1.502476 | 5.112367 | 0.022663 | 0.148713 |
| AABR07021586.1 | 1.690758 | 0.786918 | 0.034302 | 0.149297 |
| Hist1h2ail1 | 1.572571 | 3.85335 | 0.034308 | 0.149297 |
| Slc16a5 | 1.482875 | 4.898008 | 0.022861 | 0.149297 |
| AABR07012318.1 | 1.509892 | 1.453445 | 0.034368 | 0.149303 |
| Ybx3 | 2.04642 | 8.049881 | 0.02299 | 0.149567 |
| Zfp385c | 1.580525 | 6.797216 | 0.022967 | 0.149567 |
| Gm49152 | 1.37675 | 5.293526 | 0.022974 | 0.149567 |
| Snrnp70 | 1.404517 | 5.536612 | 0.023026 | 0.149635 |
| Pus3 | 1.772818 | 5.300186 | 0.023209 | 0.15027 |
| F11r | 1.516092 | 6.25089 | 0.023262 | 0.15027 |
| Col13a1 | 1.482707 | 7.917491 | 0.023319 | 0.150335 |
| H2bc12 | 1.42514 | 4.566125 | 0.034663 | 0.150408 |
| Gpx6 | 2.294414 | 6.432644 | 0.023444 | 0.150796 |
| Il17b | 2.340817 | 0.990587 | 0.03482 | 0.150951 |
| Olfr286 | 1.955548 | 9.638227 | 0.023544 | 0.151196 |
| Gm42568 | 2.302248 | 6.0292 | 0.023648 | 0.151402 |
| Gm43310 | 1.710935 | 6.890728 | 0.023653 | 0.151402 |
| Tmtc1 | 1.591811 | 6.362085 | 0.023625 | 0.151402 |
| Ror2 | 1.518218 | 3.69568 | 0.035124 | 0.151948 |
| Nckipsd | 1.547124 | 6.057708 | 0.023758 | 0.151995 |
| Plet1 | 1.538586 | 2.929745 | 0.035192 | 0.152199 |
| Fam189b | 1.375709 | 5.468111 | 0.023859 | 0.152562 |
| Gm6518 | 1.881368 | 6.402838 | 0.02397 | 0.15294 |
| Alx4 | 2.210975 | 1.105289 | 0.035892 | 0.154213 |
| Wnt7b | 1.75434 | 2.53574 | 0.035909 | 0.154241 |
| Zup1 | 1.562828 | 4.870239 | 0.024387 | 0.154341 |
| Corin | 1.382548 | 5.575637 | 0.02436 | 0.154341 |
| Gm5874 | 1.817375 | 4.898643 | 0.024454 | 0.154464 |
| Emilin1 | 1.620541 | 6.111656 | 0.024529 | 0.154472 |
| Gm43275 | 1.520365 | 5.891788 | 0.024511 | 0.154472 |
| Gm52970 | 1.391525 | 5.002088 | 0.024561 | 0.154481 |
| Gm26891 | 1.813677 | 7.409185 | 0.024652 | 0.154548 |
| F730016J06Rik | 2.350928 | 8.587115 | 0.02475 | 0.154938 |
| Chtop | 1.474638 | 5.627406 | 0.024863 | 0.155078 |
| Rdh9 | 1.418643 | 5.267743 | 0.02485 | 0.155078 |
| AC157780.1 | 1.41856 | 5.319167 | 0.024836 | 0.155078 |
| Vcp | 1.561543 | 10.10106 | 0.024911 | 0.155178 |
| Adamts16 | 1.447939 | 1.347813 | 0.036274 | 0.155256 |
| Wnt2 | 1.525714 | 1.278082 | 0.036289 | 0.155274 |
| Plxna1 | 1.658823 | 6.91811 | 0.025024 | 0.155596 |
| Tada3 | 1.406373 | 4.837195 | 0.025048 | 0.155662 |
| Cacnb4 | 2.165036 | 8.401042 | 0.025103 | 0.155679 |
| Dixdc1 | 1.731246 | 7.511954 | 0.02513 | 0.155684 |
| 4930589O11Rik | 1.527697 | 5.788129 | 0.025236 | 0.15592 |
| Heatr6 | 1.49016 | 4.84136 | 0.025293 | 0.15592 |
| Asns | 1.458938 | 5.520248 | 0.025716 | 0.157396 |
| Prune2 | 1.509171 | 4.962973 | 0.025861 | 0.157929 |
| AL627238.2 | 1.457013 | 5.220376 | 0.02589 | 0.157929 |
| Rims2 | 1.388506 | 5.150625 | 0.025963 | 0.158216 |
| Tle2 | 1.644677 | 7.043675 | 0.026028 | 0.158222 |
| Rps11-ps2 | 1.58819 | 6.22385 | 0.026063 | 0.158222 |
| Tmem43 | 1.431054 | 5.85718 | 0.026023 | 0.158222 |
| Osgin1 | 1.39899 | 4.967344 | 0.026083 | 0.158222 |
| Ache | 2.037405 | 7.139722 | 0.026441 | 0.159132 |
| Gm5173 | 1.838841 | 7.91447 | 0.026446 | 0.159132 |
| Gmcl1 | 1.679091 | 6.741695 | 0.026501 | 0.159206 |
| Zfp229 | 1.450714 | 6.021124 | 0.0265 | 0.159206 |
| Gm12320 | 1.379374 | 5.751954 | 0.026527 | 0.159213 |
| Rps15a-ps8 | 2.139097 | 5.871876 | 0.026651 | 0.159408 |
| Elmo1 | 2.027836 | 6.976288 | 0.026638 | 0.159408 |
| Slc35e2 | 1.519396 | 6.356491 | 0.026785 | 0.15988 |
| Nkiras2 | 1.572365 | 6.432264 | 0.026927 | 0.160084 |
| Klhdc8a | 1.679528 | 0.904535 | 0.038116 | 0.160184 |
| AABR07027575.1 | 1.397411 | 2.709277 | 0.038135 | 0.160184 |
| Pcdhga12 | 1.676175 | 7.111555 | 0.027198 | 0.161135 |
| Fam177a | 1.751821 | 5.085101 | 0.027263 | 0.16119 |
| Fbxo24 | 1.460407 | 5.555933 | 0.027263 | 0.16119 |
| Gm38320 | 1.45562 | 4.941343 | 0.027279 | 0.16119 |
| Gm41506 | 1.421537 | 5.635616 | 0.027329 | 0.16119 |
| Gm5821 | 1.389573 | 5.456433 | 0.027283 | 0.16119 |
| Pank3 | 1.711149 | 6.072464 | 0.027438 | 0.161357 |
| Lrrc3b | 1.540755 | 1.017847 | 0.038599 | 0.161671 |
| Mdc1 | 1.688861 | 4.938239 | 0.027655 | 0.162236 |
| Pfn4 | 1.714908 | 7.25732 | 0.027752 | 0.162388 |
| 4933411K16Rik | 1.645488 | 4.992153 | 0.027779 | 0.162405 |
| Tmod1 | 2.159229 | 7.681086 | 0.027846 | 0.162418 |
| Mmp24 | 1.572953 | 7.922398 | 0.027863 | 0.162418 |
| Krt9 | 2.519295 | 5.088683 | 0.028029 | 0.162626 |
| Shank2 | 1.415064 | 5.479202 | 0.02804 | 0.162626 |
| Xpo7 | 1.388206 | 4.641252 | 0.027959 | 0.162626 |
| Fbll1 | 1.721555 | 5.589997 | 0.028099 | 0.162663 |
| 1700003D09Rik | 1.384734 | 5.325848 | 0.028097 | 0.162663 |
| LOC102548682 | 1.411248 | 4.753057 | 0.039033 | 0.162785 |
| Chsy3 | 1.64345 | 1.171876 | 0.039082 | 0.162916 |
| Ascc3 | 1.544147 | 6.827297 | 0.028319 | 0.163094 |
| Gm40123 | 1.492187 | 5.301685 | 0.028483 | 0.163636 |
| Thoc3 | 1.646671 | 6.91358 | 0.028627 | 0.163752 |
| Cd3eap | 1.874686 | 7.726783 | 0.028775 | 0.164204 |
| Gm9639 | 1.615247 | 6.017395 | 0.028929 | 0.164507 |
| Bend5 | 1.825857 | 5.01339 | 0.029087 | 0.164865 |
| Scpep1 | 1.777136 | 6.69676 | 0.029125 | 0.164865 |
| Frmd6 | 1.573271 | 6.698178 | 0.029039 | 0.164865 |
| Gm7390 | 1.778901 | 5.329007 | 0.029245 | 0.165306 |
| Prss32 | 1.450259 | 6.135873 | 0.029368 | 0.165608 |
| Greb1 | 1.62264 | 4.866844 | 0.02943 | 0.165724 |
| Klhl34 | 1.369092 | 2.677338 | 0.040116 | 0.165909 |
| Olfr532 | 1.558697 | 6.301613 | 0.029559 | 0.166059 |
| Parp6 | 1.426815 | 5.462129 | 0.029719 | 0.1665 |
| Atp7b | 1.466671 | 1.185272 | 0.040404 | 0.166584 |
| Gm11847 | 1.815295 | 5.199898 | 0.030095 | 0.167106 |
| Adamts12 | 1.751707 | 6.83888 | 0.02997 | 0.167106 |
| Plin4 | 1.435712 | 5.548085 | 0.030032 | 0.167106 |
| Nlrx1 | 1.385535 | 5.865199 | 0.030057 | 0.167106 |
| Slc12a2 | 1.713214 | 4.945699 | 0.030121 | 0.16711 |
| Chrnb2 | 1.62677 | 5.220922 | 0.030124 | 0.16711 |
| Ccm2l | 2.301967 | 6.552961 | 0.030181 | 0.167191 |
| Arhgap33 | 1.525988 | 6.288433 | 0.03017 | 0.167191 |
| Smad9 | 1.592495 | 6.917064 | 0.030275 | 0.16756 |
| Asphd1 | 1.666764 | 7.078947 | 0.030326 | 0.167687 |
| Mettl4-ps1 | 1.655874 | 5.38036 | 0.030472 | 0.168023 |
| Rtraf-ps | 2.011511 | 7.50712 | 0.030601 | 0.168053 |
| Ppp1r13l | 1.713465 | 5.94745 | 0.030634 | 0.168053 |
| Kncn | 1.674601 | 6.437727 | 0.030579 | 0.168053 |
| Scgb1b1-ps | 1.551765 | 6.618447 | 0.030646 | 0.168053 |
| 4833422C13Rik | 2.112941 | 6.242425 | 0.030674 | 0.168128 |
| Gm2606 | 1.832784 | 5.488695 | 0.030772 | 0.168323 |
| Scd2 | 1.620102 | 6.39887 | 0.030756 | 0.168323 |
| AABR07027753.2 | 1.455072 | 4.378055 | 0.04096 | 0.168397 |
| Gpnmb | 1.819677 | 5.273239 | 0.030834 | 0.168401 |
| Iqcg | 1.518524 | 6.111419 | 0.030915 | 0.168401 |
| Inhbe | 1.463322 | 5.71097 | 0.030935 | 0.168401 |
| Stx5a | 1.454705 | 6.093964 | 0.03101 | 0.16873 |
| Spdl1 | 1.811482 | 5.880023 | 0.031045 | 0.168766 |
| Oca2 | 1.396908 | 6.106507 | 0.031077 | 0.168865 |
| Ercc1 | 1.898096 | 8.756641 | 0.031094 | 0.168881 |
| Rab2a | 1.417367 | 8.89124 | 0.031126 | 0.168977 |
| NEWGENE_6497122 | 1.651625 | 0.719047 | 0.041411 | 0.169578 |
| Hist1h2ak | 1.503327 | 2.751383 | 0.041506 | 0.169776 |
| Dmp1 | 1.579015 | 1.507339 | 0.04156 | 0.169898 |
| AC096301.1 | 1.662379 | 1.365734 | 0.04162 | 0.169994 |
| Gm20745 | 1.580741 | 6.089751 | 0.031649 | 0.170191 |
| Copb1 | 1.371088 | 5.442448 | 0.031635 | 0.170191 |
| Gm45090 | 1.639652 | 6.484738 | 0.031835 | 0.17073 |
| Gm47360 | 1.449813 | 5.333327 | 0.031877 | 0.170802 |
| Tmem52 | 1.692722 | 7.591881 | 0.032329 | 0.171758 |
| Slc24a4 | 2.27832 | 0.634219 | 0.042195 | 0.171864 |
| Rnf11 | 1.418045 | 5.924926 | 0.032481 | 0.172003 |
| Gtsf1 | 1.950052 | 6.071903 | 0.032662 | 0.172575 |
| Samd8 | 1.535711 | 8.062496 | 0.032779 | 0.172575 |
| Clic3 | 1.476945 | 5.807743 | 0.032928 | 0.17264 |
| Arrb1 | 1.781599 | 6.857494 | 0.033393 | 0.173655 |
| Slc7a8 | 1.896361 | 6.850482 | 0.03343 | 0.173745 |
| Iba57 | 1.956791 | 5.527268 | 0.033446 | 0.173751 |
| Apol7b | 1.446494 | 5.725488 | 0.033561 | 0.173957 |
| Adprhl1 | 1.419288 | 1.64671 | 0.042899 | 0.173996 |
| Itga3 | 2.290182 | 12.16349 | 0.033712 | 0.174373 |
| Col14a1 | 1.479397 | 6.376951 | 0.033864 | 0.174857 |
| Ahrr | 1.446909 | 6.1464 | 0.034296 | 0.176402 |
| Ripk2 | 1.564657 | 6.391396 | 0.034375 | 0.176504 |
| Oit1 | 1.410965 | 5.988254 | 0.034575 | 0.176769 |
| Asb16 | 1.577514 | 6.614614 | 0.03463 | 0.176934 |
| Ap1s3 | 1.59274 | 2.862493 | 0.044127 | 0.177243 |
| Tafa5 | 1.42139 | 1.665271 | 0.044212 | 0.177536 |
| Tle7 | 1.568711 | 7.140469 | 0.035328 | 0.178252 |
| Hcar1 | 1.473051 | 1.119339 | 0.044699 | 0.179047 |
| Tm4sf5 | 1.418157 | 5.556598 | 0.035566 | 0.179228 |
| Kbtbd11 | 2.061834 | 7.620401 | 0.035825 | 0.179924 |
| Gpr152 | 1.454729 | 9.035654 | 0.036386 | 0.1816 |
| Nle1 | 1.451809 | 6.347019 | 0.036507 | 0.1816 |
| Rad23b | 1.378329 | 9.721182 | 0.036484 | 0.1816 |
| Nagk | 1.652904 | 6.704662 | 0.036529 | 0.181623 |
| Cops2 | 1.892409 | 7.918415 | 0.036697 | 0.181818 |
| Bdnf | 2.135231 | 0.528373 | 0.045713 | 0.181822 |
| Kif2c | 1.460295 | 3.207321 | 0.046236 | 0.183325 |
| Lrp5 | 1.369686 | 6.196588 | 0.037359 | 0.183505 |
| Tmem255b | 1.72165 | 7.026872 | 0.037479 | 0.183974 |
| Dus3l | 1.535518 | 5.194464 | 0.037756 | 0.184691 |
| Srms | 1.648306 | 3.464465 | 0.04683 | 0.185021 |
| Gm12529 | 1.498856 | 5.172814 | 0.037989 | 0.185486 |
| Lmod2 | 2.193796 | 7.595267 | 0.038056 | 0.18551 |
| Mettl7a1 | 1.406639 | 5.693135 | 0.038105 | 0.185672 |
| Polr2g | 1.446772 | 6.565866 | 0.038136 | 0.185746 |
| H2bc9 | 1.859814 | 1.496754 | 0.047402 | 0.186488 |
| Gm38947 | 1.637197 | 7.530978 | 0.038622 | 0.187123 |
| Atg7 | 1.40873 | 6.195517 | 0.038855 | 0.18759 |
| Cdca3 | 1.386815 | 5.7892 | 0.038876 | 0.18759 |
| Dhx9 | 1.484202 | 6.887732 | 0.038897 | 0.187619 |
| Gm34280 | 1.647698 | 7.137424 | 0.03897 | 0.187673 |
| Gm44186 | 1.477986 | 5.250195 | 0.039205 | 0.188148 |
| Sorbs2 | 1.818501 | 7.960702 | 0.039596 | 0.188703 |
| Gm7368 | 1.435195 | 6.324436 | 0.039707 | 0.188726 |
| H1f10 | 1.385825 | 2.832963 | 0.048225 | 0.188883 |
| Gm14216 | 1.669012 | 4.905582 | 0.039837 | 0.188945 |
| Efna2 | 1.748193 | 6.551459 | 0.03989 | 0.189122 |
| Fbxo41 | 1.369475 | 6.347027 | 0.039965 | 0.18925 |
| AC090652.1 | 1.852379 | 5.359467 | 0.040043 | 0.189395 |
| Mcm4 | 1.813047 | 5.45266 | 0.040096 | 0.189395 |
| Gm44207 | 1.453578 | 6.448318 | 0.040058 | 0.189395 |
| 1700029H14Rik | 1.373398 | 5.144236 | 0.040195 | 0.189493 |
| Plk5 | 1.500902 | 1.4924 | 0.048651 | 0.189682 |
| Gpr21 | 1.385919 | 6.850098 | 0.040519 | 0.190289 |
| Klf4 | 1.690203 | 9.190451 | 0.040825 | 0.191124 |
| Mtbp | 1.496343 | 6.490911 | 0.041094 | 0.191857 |
| Ogdh | 1.701498 | 8.378967 | 0.041669 | 0.191867 |
| Tbc1d10b | 1.53561 | 11.02215 | 0.04156 | 0.191867 |
| Acer1 | 1.516764 | 6.884664 | 0.04132 | 0.191867 |
| Gm31812 | 1.486052 | 6.065863 | 0.041611 | 0.191867 |
| Clhc1 | 1.461671 | 5.55369 | 0.041497 | 0.191867 |
| Nemp1 | 1.438064 | 5.212429 | 0.041742 | 0.191867 |
| Mir695 | 1.413294 | 4.82802 | 0.041573 | 0.191867 |
| Grpel1 | 1.475669 | 6.767185 | 0.04194 | 0.192019 |
| Col5a2 | 1.443822 | 8.333327 | 0.041906 | 0.192019 |
| Gpr75 | 1.763091 | 7.909305 | 0.041968 | 0.192032 |
| Slc39a6 | 1.42772 | 6.635087 | 0.049752 | 0.192317 |
| AC127084.5 | 1.870815 | 0.807637 | 0.049962 | 0.193025 |
| Wnt3a | 2.204518 | 7.10222 | 0.042486 | 0.193143 |
| 2310033P09Rik | 2.14027 | 6.069326 | 0.042803 | 0.193554 |
| Emc1 | 1.447819 | 6.958169 | 0.043351 | 0.194853 |
| Cd3e | 1.482462 | 7.091341 | 0.043871 | 0.195859 |
| Srl | 1.389189 | 5.796729 | 0.044058 | 0.196253 |
| 5830468F06Rik | 1.716187 | 5.563745 | 0.044194 | 0.196569 |
| Wdr45 | 1.555592 | 6.324587 | 0.044262 | 0.19665 |
| Ikbkb | 1.48738 | 7.064032 | 0.044431 | 0.197189 |
| Gm38409 | 1.444346 | 5.94257 | 0.044446 | 0.197189 |
| Pigm | 1.947041 | 8.904324 | 0.045066 | 0.1987 |
| Kat5 | 1.72589 | 7.078143 | 0.045166 | 0.198834 |
| Fam169a | 1.505125 | 5.303651 | 0.045157 | 0.198834 |
| Myorg | 1.412857 | 6.472381 | 0.04517 | 0.198834 |
| Gm7285 | 1.42009 | 5.731183 | 0.045883 | 0.200566 |
| Fastkd3 | 1.823763 | 5.453069 | 0.046166 | 0.20069 |
| Cd163 | 1.506559 | 4.725869 | 0.046209 | 0.20069 |
| Cnot8 | 1.450041 | 5.761135 | 0.046141 | 0.20069 |
| Cenpt | 2.194958 | 6.771158 | 0.046413 | 0.201192 |
| Arl5a | 2.265642 | 7.743151 | 0.047068 | 0.20213 |
| Ppp3cc | 1.472914 | 6.385484 | 0.047031 | 0.20213 |
| Gm11684 | 1.395938 | 7.094789 | 0.046891 | 0.20213 |
| Gm3924 | 1.859622 | 7.60911 | 0.04745 | 0.203251 |
| 1810013L24Rik | 1.387621 | 5.976971 | 0.047541 | 0.203353 |
| Men1 | 2.142315 | 9.831001 | 0.048275 | 0.205166 |
| Kcnc1 | 1.462111 | 6.575915 | 0.048696 | 0.205783 |
| Gm17756 | 1.653865 | 4.938918 | 0.048837 | 0.206046 |
| Akt1 | 1.561679 | 7.410283 | 0.049022 | 0.206356 |
| Txnrd2 | 1.450981 | 7.093149 | 0.049532 | 0.207483 |
| Col1a2 | 1.99628 | 11.66273 | 0.04962 | 0.207483 |
