# Supplementary material for: Drp1 activates ROS/HIF-1α/EZH2 and triggers mitochondrial fragmentation to deteriorate hypercalcemia-associated neuronal injury in mouse model of chronic kidney disease
Source: J Neuroinflammation. 2022 Sep 1;19:213. doi: 10.1186/s12974-022-02542-7 (PMC9438241; doi:10.1186/s12974-022-02542-7)
Supplement: Supplementary file 5 — Additional file 5: Table S4. Binding sites between HIF1A and EZH2_Promoter. [file 12974_2022_2542_MOESM5_ESM.docx]

**Table S4** Binding sites between HIF1A and EZH2_Promoter

| Matrix ID | Name | Score | Relative score | Sequence ID | Start | End | Strand | Predicted sequence |
| --- | --- | --- | --- | --- | --- | --- | --- | --- |
| MA1106.1 | HIF1A | 10.9173 | 0.949177857 | EZH2_Promoter | 818 | 827 | + | acacgtgctt |
| MA1106.1 | HIF1A | 7.66111 | 0.872508113 | EZH2_Promoter | 1771 | 1780 | + | gcacgcgctg |
| MA1106.1 | HIF1A | 7.53137 | 0.869453411 | EZH2_Promoter | 816 | 825 | - | gcacgtgtca |
| MA1106.1 | HIF1A | 6.89238 | 0.854407858 | EZH2_Promoter | 1864 | 1873 | + | ggacgggcgc |
| MA1106.1 | HIF1A | 5.92822 | 0.831705847 | EZH2_Promoter | 1769 | 1778 | - | gcgcgtgcgc |
| MA1106.1 | HIF1A | 5.32777 | 0.81756767 | EZH2_Promoter | 1796 | 1805 | + | ccacgagccc |
| MA1106.1 | HIF1A | 5.0384 | 0.810754154 | EZH2_Promoter | 1971 | 1980 | - | ggacgcgacc |
| MA1106.1 | HIF1A | 4.82398 | 0.805705429 | EZH2_Promoter | 1645 | 1654 | + | ccgcgtgcct |
| MA1106.1 | HIF1A | 4.65758 | 0.801787498 | EZH2_Promoter | 1877 | 1886 | - | ggacgcgagg |
